# Acid/base-regulated reversible electron transfer disproportionation of N–N linked bicarbazole and biacridine derivatives†
†Electronic supplementary information (ESI) available: Experimental information, synthesis and characterization data, NMR spectra, solid state NMR data, X-ray data, ESR spectra, UV-Vis-NIR spectra, fluorescence spectra, kinetic experiments, theoretical calculations, Tables S1–S8, Scheme S1, Fig. S1–12, References. CCDC 1025063, 1038914, 1049677 and 1040722. For ESI and crystallographic data in CIF or other electronic format see DOI: 10.1039/c5sc00946d


**DOI:** 10.1039/c5sc00946d

**Published:** 2015-05-21

**Authors:** Palash Pandit, Koji Yamamoto, Toshikazu Nakamura, Katsuyuki Nishimura, Yuki Kurashige, Takeshi Yanai, Go Nakamura, Shigeyuki Masaoka, Ko Furukawa, Yumi Yakiyama, Masaki Kawano, Shuhei Higashibayashi

**Affiliations:** a Institute for Molecular Science , Myodaiji , Okazaki 444-8787 , Japan . Email: higashi@ims.ac.jp; b School of Physical Sciences , The Graduate University for Advanced Studies , Myodaiji , Okazaki 444-8787 , Japan; c Japan Science and Technology Agency , PRESTO, 4-1-8 Honcho , Kawaguchi , Saitama 332-0012 , Japan; d Center for Instrumental Analysis , Institute for Research Promotion , Niigata University , Nishi-ku , Niigata 950-2181 , Japan; e Division of Advanced Materials Science , Pohang University of Science and Technology , San 31, Hyojadong , Pohang 790-784 , Korea; f Japan Science and Technology Agency , ACT-C, 4-1-8 Honcho , Kawaguchi , Saitama 332-0012 , Japan

## Abstract

Regulation of electron transfer on organic substances by external stimuli is a fundamental issue in science and technology, which affects organic materials, chemical synthesis, and biological metabolism. Nevertheless, acid/base-responsive organic materials that exhibit reversible electron transfer have not been well studied and developed, owing to the difficulty in inventing a mechanism to associate acid/base stimuli and electron transfer. We discovered a new phenomenon in which N–N linked bicarbazole (BC) and tetramethylbiacridine (TBA) derivatives undergo electron transfer disproportionation by acid stimulus, forming their stable radical cations and reduced species. The reaction occurs through a biradical intermediate generated by the acid-triggered N–N bond cleavage reaction of BC or TBA, which acts as a two electron acceptor to undergo electron transfer reactions with two equivalents of BC or TBA. In addition, in the case of TBA the disproportionation reaction is highly reversible through neutralization with NEt_3_, which recovers TBA through back electron transfer and N–N bond formation reactions. This highly reversible electron transfer reaction is possible due to the association between the acid stimulus and electron transfer *via* the acid-regulated N–N bond cleavage/formation reactions which provide an efficient switching mechanism, the ability of the organic molecules to act as multi-electron donors and acceptors, the extraordinary stability of the radical species, the highly selective reactivity, and the balance of the redox potentials. This discovery provides new design concepts for acid/base-regulated organic electron transfer systems, chemical reagents, or organic materials.

## Abstract 2

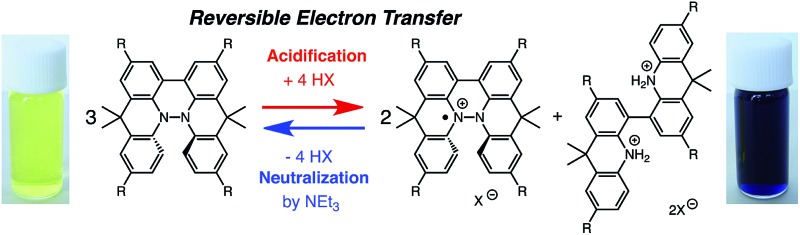
New acid/base-responsive organic compounds were discovered to undergo electron transfer disproportionation.

## Introduction

Regulation of the electron transfer redox process on organic substances by external stimuli (light, electric field, pressure, pH, chemicals, *etc.*) is a fundamental issue in both science and technology, which affects organic materials, chemical synthesis, and biological metabolism.^1^ The development of redox-active organic compounds and assembled systems that show functional responses to external stimuli leads to wide applications. Among the external stimuli, the control of the electron transfer redox reaction on organic substances by light or electric field has been extensively studied and developed for organic materials/devices and chemical syntheses.1a,^2^ Light and electric field directly induce the electron transfer redox reaction, which is followed by the functional response. In contrast, it is more difficult to design and develop redox-active organic compounds responsive to stimuli such as acid/base^3^–^5^ or other chemicals,^6^,^7^ because these stimuli do not directly induce electron transfer or redox conversion, but rather protonation, complexation, or adsorption. Thus, for regulation by these stimuli it is necessary to connect the chemical or physical changes to the electron transfer or redox transformation, which is followed by the functional response. Other important factors are the reversibility or repeatability of the reaction and the sustainability of the response of the system, which require either the redox reaction process to be reversible in the presence of the opposite stimulus (*e.g.*, neutralization) or the responsive material to be a catalyst that repeatedly undergoes a redox reaction. These requirements make the development of acid/base-responsive organic materials with multi-functional properties very difficult. Although many acid-responsive organic compounds, including pH indicators, have been developed,^8^ acids regulate non-redox processes such as isomerization, complexation, or conformational change, followed by the functional response. Only tetrathiafulvalene (TTF)^3^ and 2,2,6,6-tetramethyl-1-piperidinyloxyl (TEMPO)^4^ among synthetic organic compounds have been reported to exhibit an acid-responsive reversible electron transfer reaction through disproportionation, owing to their excellent redox properties (Scheme S1†).^5^ However, the reaction is either very low yielding (∼1%) or requires very strong acids (conc. H_2_SO_4_) due to the simple protonation and subsequent electron transfer mechanism. To realize a more efficient acid-responsive electron transfer system, a sophisticated mechanism to associate the acid/base stimuli with an electron transfer reaction is necessary. As examples of other chemical stimuli, metal ion-promoted^6^ or anion-mediated^7^ electron transfer has been reported in donor–acceptor type TTF derivatives, utilizing conformational change or supramolecular assembly induced by metal ion or anion complexation as the efficient switching mechanism.

**Fig. 1:**
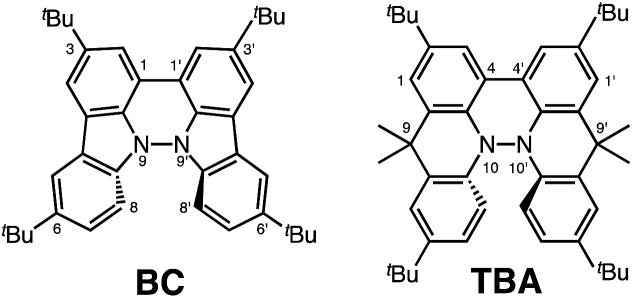
1,1′,9,9′-Bicarbazole (BC) and tetramethyl-4,4′,10,10′-biacridine (TBA) derivatives with ^*t*^Bu substituents.

**Scheme 1:**
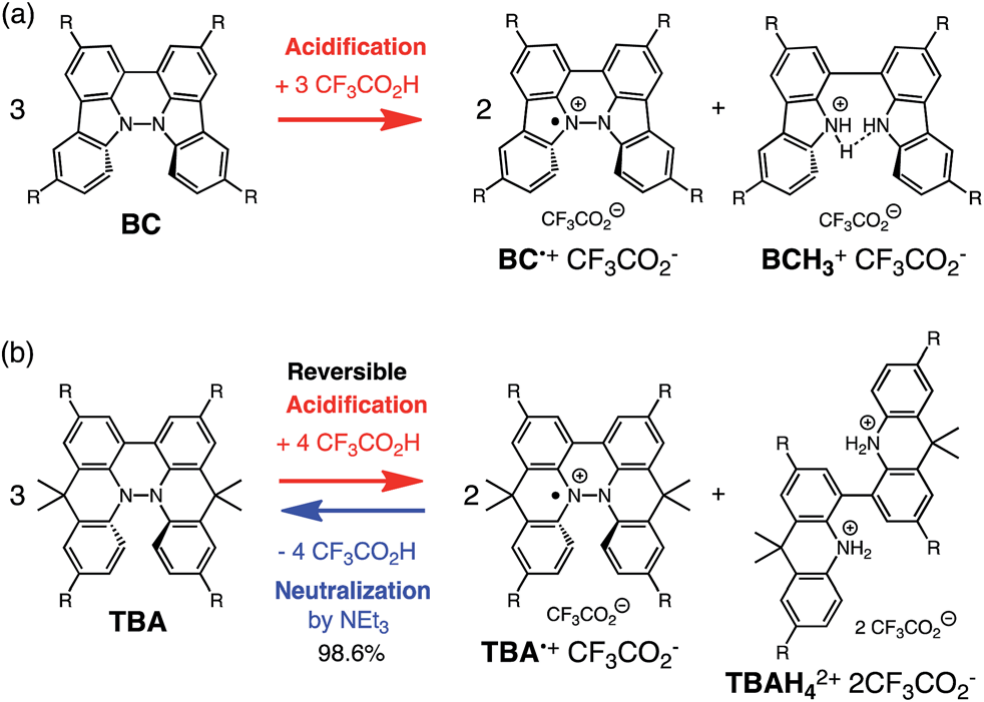
Acid/base-regulated electron transfer disproportionation of (a) BC and (b) TBA.

During the course of our study of N–N linked 1,1′,9,9′-bicarbazole (BC) and 9,9,9′,9′-tetramethyl-4,4′,10,10′-biacridine (TBA) derivatives (Fig. 1), we encountered an unexpected phenomenon in that their ^1^H NMR spectra in CDCl_3_ showed extremely broad signals, which turned out to be due to the acid-responsive generation of unknown radical species. The unknown radical species generated by addition of acids in organic solvents were highly stable in air at room temperature under acidic conditions. Furthermore, to our surprise, BC or TBA was recovered in high yields on neutralization with NEt_3_. After thorough experimental investigations and computational studies, this phenomenon was fully elucidated as being due to acid-responsive electron transfer disproportionation to give stable radical cations and reduced species (Scheme 1). These compounds exhibit contrasting photophysical and magnetic properties before and after the reaction. The reaction occurs through a biradical intermediate generated by the acid-triggered N–N bond cleavage reaction of BC or TBA, which acts as a two electron acceptor and undergoes electron transfer reactions with two equivalents of BC or TBA to produce radical cations and reduced species. This electron transfer disproportionation reaction is possible due to the association between the acid stimulus and electron transfer *via* the acid-triggered N–N bond cleavage reaction, the ability of the organic molecules to act as multi-electron donors and acceptors, the extraordinary stability of the radical species, and the highly selective reactivity. While BC and TBA exhibited similar disproportionation reactions, several differences were observed, and the most notable difference is the reversibility of the reaction. The disproportionation reaction in TBA was found to be highly reversible through neutralization with NEt_3_, recovering TBA through back electron transfer and N–N bond formation reactions. This high reversibility was realized by the acid-regulated N–N bond cleavage/formation reactions, which provided an efficient switching mechanism, and the balance of the redox potentials of the chemical species involved. Here, we report the full identification of these compounds and the phenomenon and its mechanism through thorough experimental investigations and theoretical calculations.

## Results and discussion

### Synthesis

**Scheme 2:**
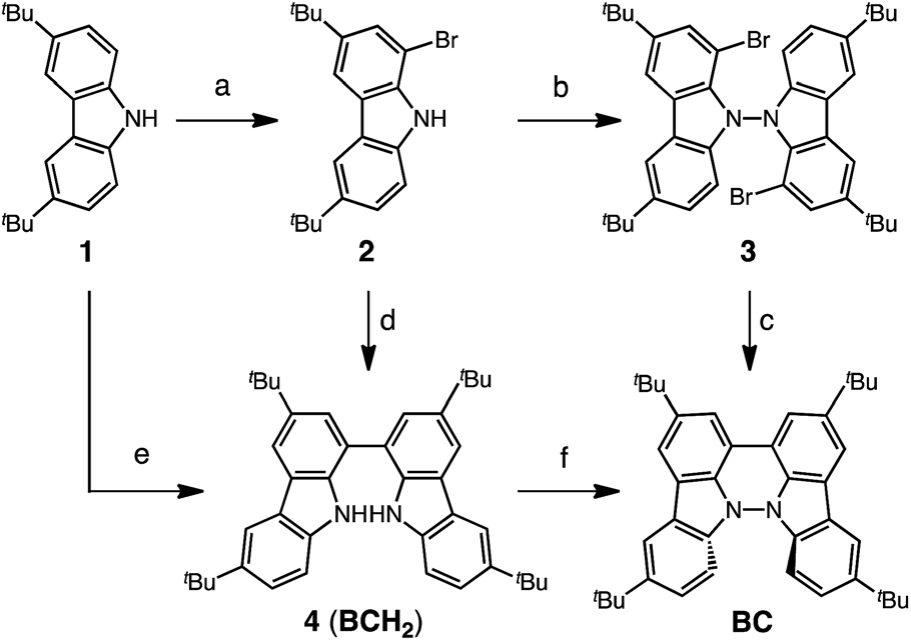
Synthesis of bicarbazole (BC). Reagents and conditions: (a) *N*-bromosuccinimide (110 mol%), SiO_2_, CH_2_Cl_2_, rt, 4 h, 86%; (b) KMnO_4_ (250 mol%), acetone, 60 °C, 4 h, 75%; (c) Ni(COD)_2_ (150 mol%), COD (150 mol%), 2,2′-bipyridyl (150 mol%), THF, 45 °C, 6 h, 69%; (d) Ni(COD)_2_ (300 mol%), COD (300 mol%), 2,2′-bipyridyl (300 mol%), THF, 80 °C, 6 h, 87%; (e) FeCl_3_ (200 mol%), CH_2_Cl_2_, rt, 15 min, 41%; (f) Bu_4_NMnO_4_ (200 mol%), pyridine, 70 °C, 24 h, 65%.

**Scheme 3:**
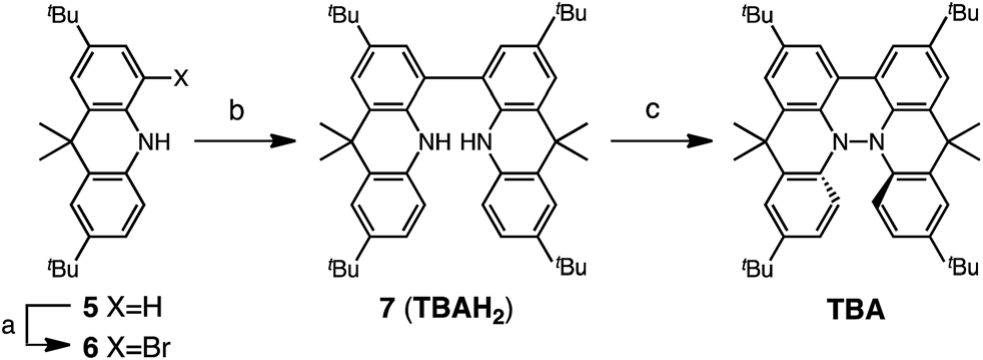
Synthesis of tetramethylbiacridine (TBA). Reagents and conditions: (a) *N*-bromosuccinimide (103 mol%), CHCl_3_, 60 °C, 1 h, 57%; (b) Ni(COD)_2_ (300 mol%), COD (300 mol%), 2,2′-bipyridyl (300 mol%), THF, 80 °C, 6 h, 95%; (c) Bu_4_NMnO_4_ (250 mol%), pyridine, rt, 12 h, 86%.

Several synthetic pathways to 1,1′,9,9′-bicarbazole (BC) with *t*-Bu groups from 3,6-di-*t*-butylcarbazole **1** were developed (Scheme 2). Bromocarbazole **2**, prepared from **1**, was converted to dimer **3** in 75% yield through the oxidative coupling of the nitrogen atoms by KMnO_4_ 9 in acetone. Ni(COD)_2_-mediated reductive coupling of **3** afforded the desired BC in 69% yield. In another pathway, dimer **4** was obtained from **2** in 87% yield by Ni(COD)_2_-mediated reductive coupling. Dimer **4** was also synthesized in 41% yield through the direct oxidative coupling of carbazole **1** using FeCl_3_. Oxidative coupling of the nitrogen atoms in **4** using Bu_4_NMnO_4_ in pyridine afforded BC in 65% yield. Tetramethyl-4,4′,10,10′-biacridine (TBA) was synthesized from 2,7-di-*tert*-butyl-9,10-dihydro-9,9-dimethylacridine **5** (Scheme 3). Bromoacridine **6**, obtained from **5** by bromination, was converted to dimer **7** in 95% yield by Ni(COD)_2_-mediated reductive coupling. Oxidative coupling of **7** using Bu_4_NMnO_4_ in pyridine afforded TBA in 86% yield.

### Disproportionation of 1,1′,9,9′-bicarbazole (BC)

**Fig. 2:**
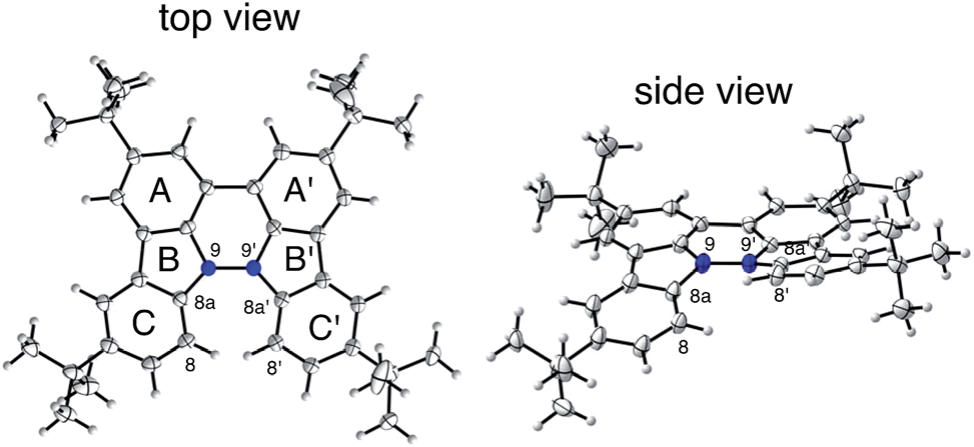
ORTEP drawings of BC at 50% probability level obtained by X-ray crystallographic analysis. The disorder of one of the *t*-butyl groups is omitted for clarity.

**Fig. 3:**
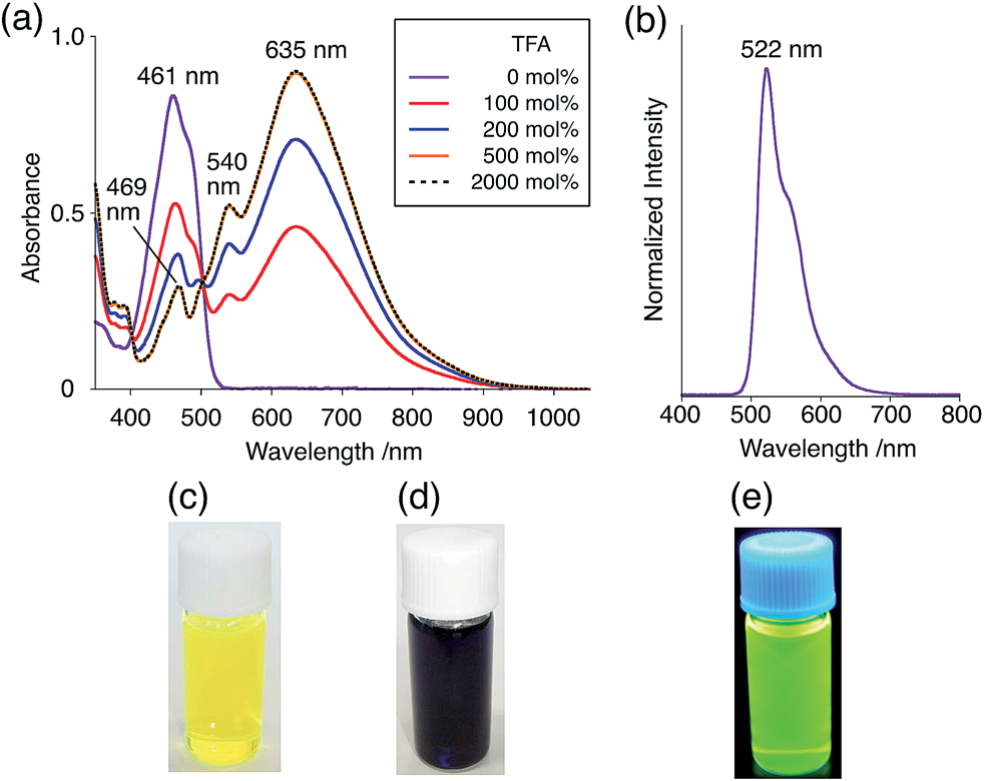
(a) UV-Vis-NIR spectral changes of BC (1.00 mM) on addition of 0, 100, 200, 500, and 2000 mol% CF_3_CO_2_H in CH_2_Cl_2_ measured with a 1 mm cell. (b) Emission spectrum of BC (0.18 mM) in CH_2_Cl_2_ (excited at 460 nm). (c) Photo of the solution of BC (1.0 mM) in CH_2_Cl_2_. (d) Photo of the solution of BC (1.0 mM) with 2000 mol% CF_3_CO_2_H in CH_2_Cl_2_. (e) Photo of the solution of BC (1.0 mM) in CH_2_Cl_2_ under UV light.

**Fig. 4:**
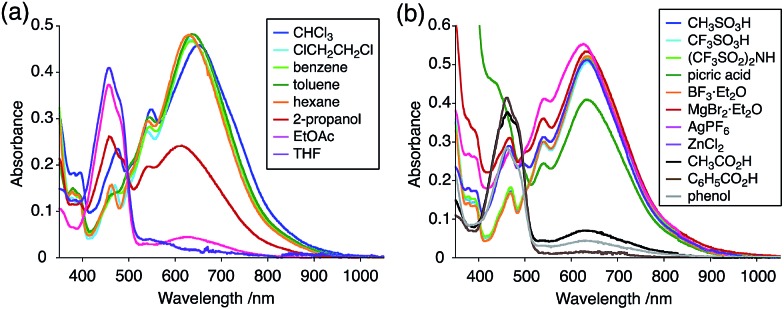
(a) UV-Vis-NIR spectral changes of BC (5.00 × 10^–2^ mM) on addition of 2000 mol% CF_3_CO_2_H in organic solvents. (b) UV-Vis-NIR spectral changes of BC (5.00 × 10^–2^ mM) on addition of 2000 mol% acids (10 000 mol% picric acid and CH_3_CO_2_H) in CH_2_Cl_2_.

**Fig. 5:**
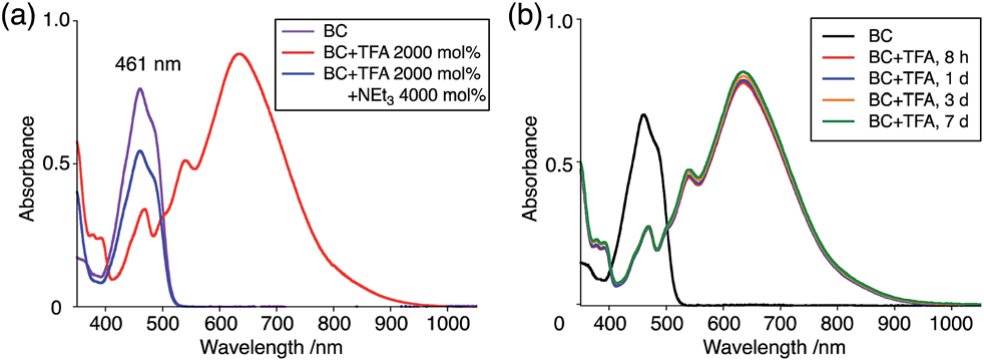
(a) UV-Vis-NIR spectra of BC (1.00 mM), BC with 2000 mol% CF_3_CO_2_H, and BC with 2000 mol% CF_3_CO_2_H followed by addition of 4000 mol% NEt_3_ in CH_2_Cl_2_ measured with a 1 mm cell. (b) UV-Vis-NIR spectra of BC (1.00 mM) with 2000 mol% CF_3_CO_2_H in CH_2_Cl_2_ after 8 h, 1 day, 3 days, and 7 days in the dark at 20 °C under air measured with a 1 mm cell.

**Fig. 6:**
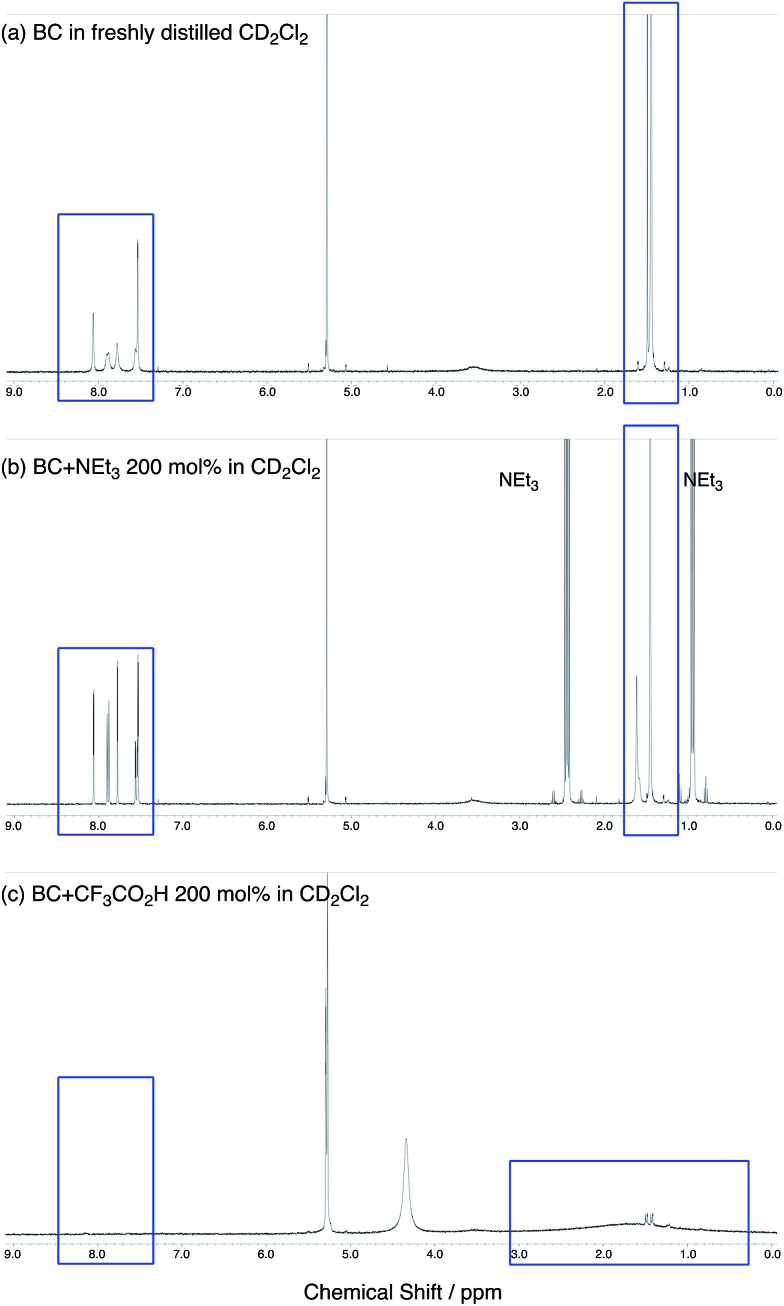
(a) ^1^H NMR spectrum of BC in freshly distilled CD_2_Cl_2_. (b) ^1^H NMR spectrum of BC with 200 mol% NEt_3_ in CD_2_Cl_2_. (c) ^1^H NMR spectrum of BC with 200 mol% CF_3_CO_2_H in CD_2_Cl_2_.

**Fig. 7:**
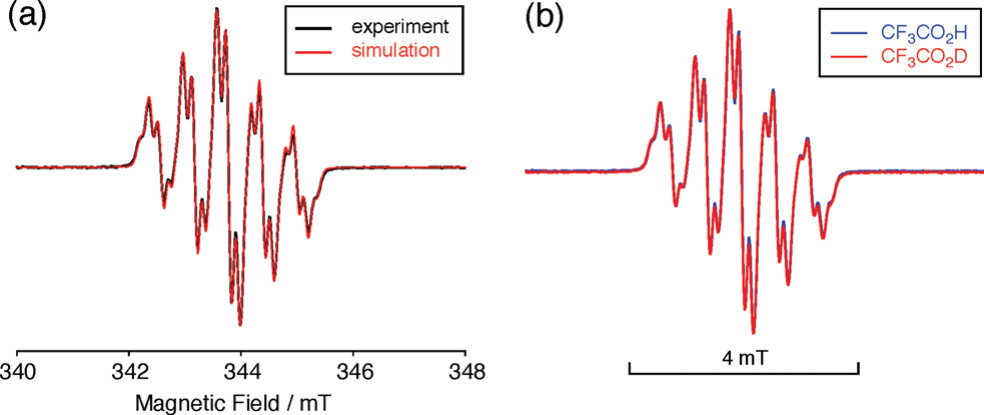
(a) ESR spectrum of BC (1.02 mM) with 2000 mol% CF_3_CO_2_H in CH_2_Cl_2_ at room temperature (X-band, *ν* = 9.637256 GHz, *g* = 2.0030) and the simulated spectrum (*S* = 1/2, hfc *a* = 6.0 G with two nitrogens, and 1.8, 1.4, 0.2, 0.2, and 0.2 G with ten hydrogens, Gaussian linewidth = 0.119 mT, Lorentzian linewidth = 0.019 mT). (b) Comparison of the ESR spectra of BC with 2000 mol% CF_3_CO_2_H and CF_3_CO_2_D in CH_2_Cl_2_ at room temperature.

The X-ray crystallographic analysis showed a characteristic helical molecular shape of BC, with the dihedral angle ∠C_8a_N_9_N_9′_C_8a′_ = 48° due to the steric repulsion between the C and C′ rings (C_8_–C_8′_ distance = 3.33 Å) (Fig. 2). The color of the solution of BC in CH_2_Cl_2_ was yellow, and the UV-Vis-NIR absorption spectrum showed absorption at 461 nm (Fig. 3a and c). BC exhibited strong green fluorescence with an emission maximum at 522 nm in CH_2_Cl_2_ (Fig. 3b and e), and the quantum yield was determined to be 69%. While the structure of BC was unambiguously determined by X-ray crystallographic analysis, the ^1^H NMR spectrum in CDCl_3_ showed extremely broad signals (ESI†). In contrast, the solid state ^13^C, ^1^H, and ^15^N NMR spectra of BC showed the expected signals associated with the structure (Table S1 and Fig. S1–S4†). In order to explain this unexpected phenomenon, we investigated the effects of potential factors such as light, air, and solvent, and the origin of the broadening turned out to be a hydrochloric acid contaminant in the CDCl_3_. Thus, we examined the effects of acids on the physical properties of BC. When the solution of BC in CH_2_Cl_2_ was treated with CF_3_CO_2_H (TFA) at room temperature under either aerobic or anaerobic conditions, the color of the solution drastically changed from yellow to deep indigo-blue (Fig. 3d). In the UV-Vis-NIR spectrum recorded in CH_2_Cl_2_, the absorption of BC at 461 nm decreased on addition of CF_3_CO_2_H and new broad absorptions at 540 and 635 nm appeared in the visible to near-infrared light region (Fig. 3a). The intensities of the new absorption bands increased on addition of more CF_3_CO_2_H, and they were nearly saturated on addition of 500 mol% CF_3_CO_2_H. In accordance with the absorption spectral change, the emission of BC also disappeared after the addition of CF_3_CO_2_H. Similar changes in the appearance and absorption spectra were also observed in other organic solvents (CHCl_3_, 1,2-dichloroethane, benzene, toluene, hexane, 2-propanol) or with other Brønsted acids [CH_3_SO_3_H, CF_3_SO_3_H, (CF_3_SO_2_)_2_NH, picric acid], as well as with Lewis acids [BF_3_·OEt_2_, MgBr_2_·OEt_2_, AgPF_6_, ZnCl_2_], but almost no change or slight change was observed with ethyl acetate, THF, CH_3_CO_2_H, C_6_H_5_CO_2_H, or phenol (Fig. 4). The dependence of the spectral change on the amount of acid indicated that the reaction is at equilibrium under acidic conditions (Fig. 3a and S7a†). The yellow color of BC and its absorption in the UV-Vis-NIR spectrum were in turn recovered by addition of NEt_3_ to neutralize the CF_3_CO_2_H (Fig. 5a). The recovery yield of BC was determined to be 72% based on the absorption intensity at 461 nm. The ^1^H NMR spectrum of BC in freshly distilled CD_2_Cl_2_ showed slightly broad signals associated with BC, and the signals became sharper on addition of 200 mol% NEt_3_ to neutralize the trace amount of contaminant acid (Fig. 6). In contrast, the addition of 200 mol% CF_3_CO_2_H resulted in disappearance of the signals due to significant broadening, which suggests the generation of a paramagnetic radical species. This result prompted us to measure the ESR spectrum, in which a signal due to the radical species formed by BC in the presence of CF_3_CO_2_H in CH_2_Cl_2_ was observed (Fig. 7a). These results clearly demonstrate that the acid-responsiveness of BC is not caused by simple protonation/deprotonation or tautomerization, but is the result of an acid-responsive generation of a radical species involving the homolytic cleavage of a bond or electron transfer of BC under equilibrium. In addition, a surprising observation is the remarkably high stability of the radical species. These experiments can be conducted under air at room temperature without special handling, and no decomposition occurs. Indeed, the UV-Vis-NIR spectra of BC in the presence of CF_3_CO_2_H in CH_2_Cl_2_ scarcely changed even after 7 days in the dark at room temperature under air (Fig. 5b), indicating the extremely high stability of the radical species. BC is also recovered from the generated radical species in 72% yield by neutralization with NEt_3_. To elucidate this phenomenon, we further investigated the generated species and the reaction.

**Fig. 8:**
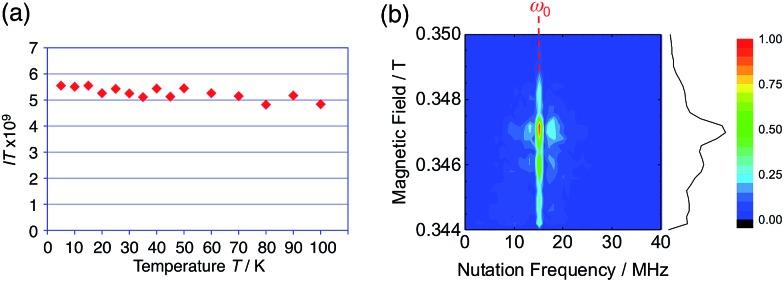
(a) Temperature dependence of the *IT* value (*I* = double integral of the ESR signal, *T* = temperature) of BC (1.02 mM) with 2000 mol% CF_3_CO_2_H in frozen CH_2_Cl_2_ at 5–100 K. (b) Electron spin transient nutation (ESTN) spectrum of BC (1.0 mM) with 200 mol% CF_3_CO_2_H in frozen toluene at 5 K.

**Fig. 9:**
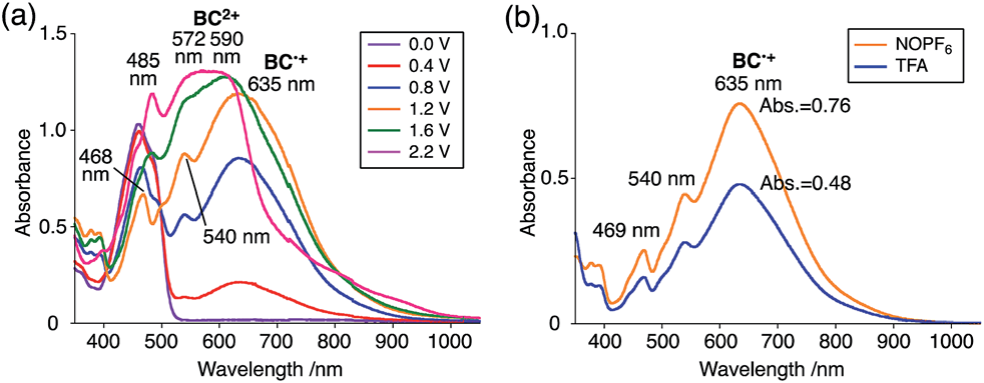
(a) UV-Vis-NIR spectra of BC˙^+^ and BC^2+^ obtained by electrochemical oxidation (*vs.* Ag/AgCl) with a Pt electrode in 1,2-dichloroethane containing 0.1 M Bu_4_NClO_4_. (b) UV-Vis-NIR spectra of BC˙^+^ (5.00 × 10^–2^ mM) obtained by chemical oxidation using 150 mol% NOPF_6_, and of BC (5.00 × 10^–2^ mM) with 2000 mol% CF_3_CO_2_H in CH_2_Cl_2_.

**Fig. 10:**
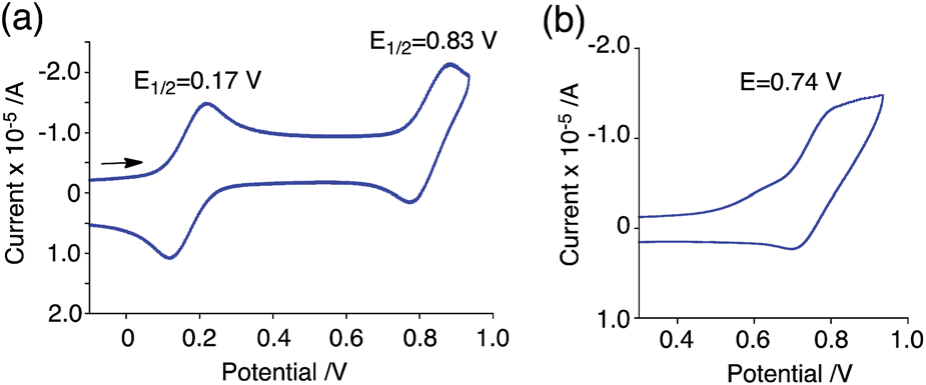
CV of (a) BC and (b) BCH_2_ in CH_2_Cl_2_ containing 0.1 M Bu_4_NClO_4_ with a glassy carbon electrode (*vs.* Fc/Fc^+^).

**Fig. 11:**
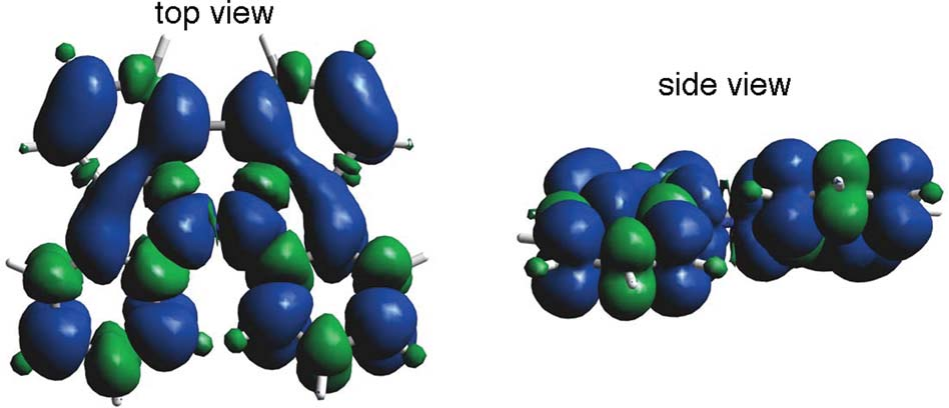
Calculated spin density distribution of BC˙^+^ [UωB97XD/6-31G(d)]. Blue and green colors indicate positive and negative spin density, respectively.

**Fig. 12:**
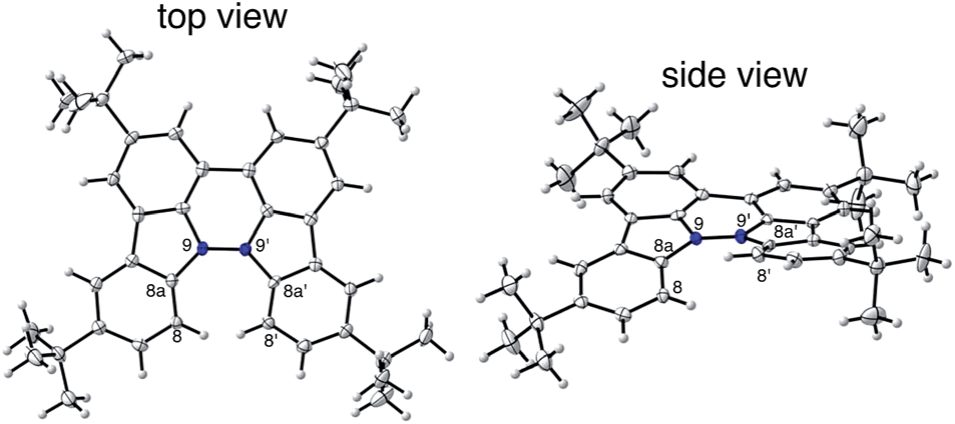
ORTEP drawings of the cation radical complex BC˙^+^I_5_^–^ at 50% probability level obtained from X-ray crystallographic analysis. Disordered I_5_^–^ and iodobenzene are omitted for clarity.

The generated radical species was characterized and assigned as the mono-radical cation BC˙^+^ (Scheme 1a) from the following results. The nearly quintet ESR signal (Fig. 7a) indicates delocalization over the bicarbazole structure with hyperfine splitting due to two nitrogen atoms. The ESR signal of BC with CF_3_CO_2_D is almost identical to that with CF_3_CO_2_H (Fig. 7b), showing that the nitrogen atom is not protonated, since no hyperfine splitting due to the proton is observed. No zero-field splitting of the signal obtained at 5 K in frozen CH_2_Cl_2_ (Fig. S5a†) and no forbidden Δ*m*_s_ = ±2 half-field transitions were observed, indicating that the spin state is a doublet (*S* = 1/2). The lack of temperature dependence of the *IT* value (*I* = double integral of the ESR signal, *T* = temperature) at 5–100 K in frozen CH_2_Cl_2_ supports the doublet spin state (Fig. 8a). For further confirmation, the electron spin transient nutation (ESTN) spectrum based on the pulsed-ESR technique was measured at 5 K. (Fig. 8b). In the ESTN spectrum, a signal was observed at the nutation frequency *ω*_0_ = 15.2 MHz (*S* = 1/2), but not at *ω*_1_ = √2*ω*_0_ = 21.5 MHz (*S* = 1), confirming the doublet spin. Finally, the identity of the doublet spin radical was determined to be the cation radical BC˙^+^, due to the fact that its UV-Vis-NIR spectrum agrees with the UV-Vis-NIR spectrum of BC˙^+^ generated by the electrochemical or chemical oxidation of BC (Fig. 9). The cyclic voltammogram (CV) of BC shows two reversible oxidation waves at 0.17 V and 0.83 V (*vs.* Fc/Fc^+^), in which BC is oxidized to BC˙^+^ and BC^2+^, respectively (Fig. 10a). Based on the CV results, BC˙^+^ and BC^2+^ were generated by the electrolysis of BC, and their UV-Vis-NIR spectra were measured. The UV-Vis-NIR spectrum of electrochemically generated BC˙^+^ with an absorption maximum at 635 nm is nearly identical to that of BC with CF_3_CO_2_H in 1,2-dichloroethane (Fig. 4a). Chemical oxidation of BC by NOPF_6_ (Fig. 9b) and I_2_ (Fig. S7b†) also generated BC˙^+^, which shows absorption at 635 nm, although the absorption overlaps with that of I_2_ in the case of I_2_ oxidation. Simulation (EasySpin)^10^ of the ESR signal of BC˙^+^ afforded the hyperfine coupling (hfc) constants *a* = 6.0 G due to coupling to two nitrogen nuclear spins, and 1.8, 1.4, 0.2, 0.2, and 0.2 G due to coupling to ten hydrogens (Fig. 7a). DFT calculations on BC˙^+^ [UωB97XD/6-31G(d)] showed that the spin density is delocalized over the whole bicarbazole skeleton (Fig. 11). The structure of BC˙^+^ was confirmed by X-ray crystallographic analysis of a single crystal of the cation radical complex BC˙^+^I_5_^–^·IC_6_H_5_ obtained through oxidation of BC with I_2_ in iodobenzene–MeOH (Fig. 12, S5b, c and S7c†). As a characteristic feature of BC˙^+^, the N–N bond length (1.35 Å) and the ∠C_8a_N_9_N_9′_C_8a′_ dihedral angle (21°) are shorter and narrower than the 1.41 Å and 48° of BC, which agrees with the trend (BC˙^+^: 1.35 Å and 18°, BC: 1.39 Å and 42°) predicted by DFT calculations.

**Fig. 13:**
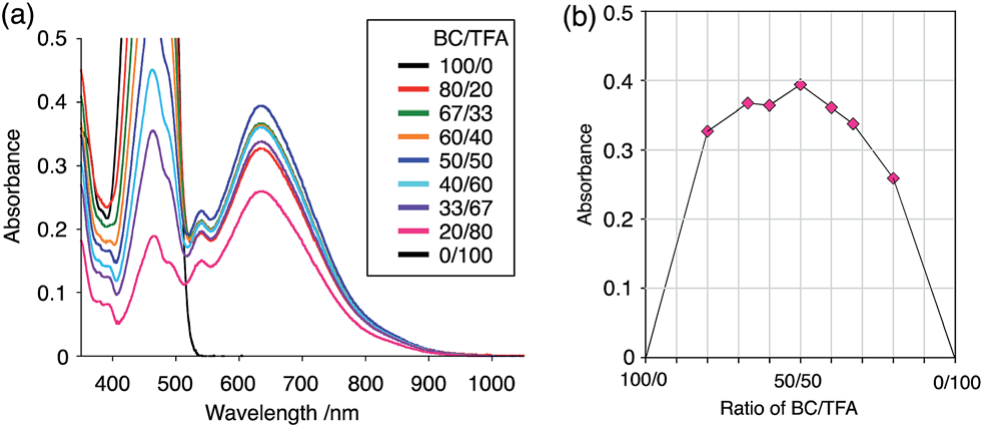
(a) Continuous variation of the UV-Vis-NIR spectra in CH_2_Cl_2_ by changing the BC/CF_3_CO_2_H ratio. The concentration of BC + CF_3_CO_2_H was 2.00 mM, and a 1 mm cell was used. (b) Continuous variation plot of the absorbance at 635 nm *versus* the BC/CF_3_CO_2_H ratios from (a).

**Scheme 4:**
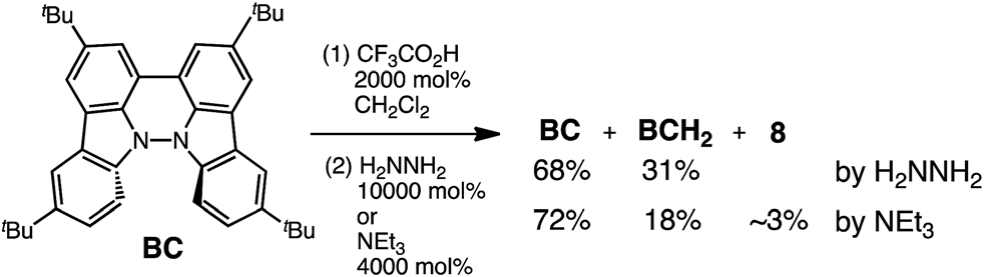
Quenching of the disproportionation of BC.

**Fig. 14:**
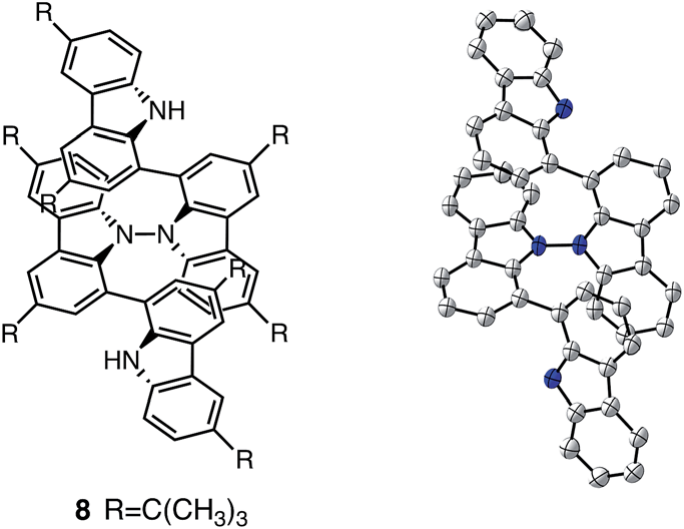
Structure of **8** and ORTEP drawing of **8** at 50% probability level obtained from X-ray crystallographic analysis. Hydrogens and *t*-butyl groups are omitted for clarity.

While BC˙^+^ is generated through the one-electron oxidation of BC under acidic conditions, we wondered what the counter oxidant (electron acceptor) was, since there was no added oxidant. The possibility of acid, solvent, or air being the oxidant was discounted because the reaction takes place under various conditions using different kinds of acids or solvents, even in an anaerobic atmosphere. By combining the experimental and computational data, we finally determined that the reaction is the disproportionation of BC, in which BC acts as both a one-electron reductant (donor) and a two-electron oxidant (acceptor) (Scheme 1a). In this equation, one equivalent of BC is reduced to BCH_3_^+^ and two equivalents of BC are oxidized to BC˙^+^ in the presence of three equivalents of CF_3_CO_2_H, with the result that three equivalents of BC react with three equivalents of CF_3_CO_2_H. This reaction stoichiometry (BC : CF_3_CO_2_H = 1 : 1) was determined by a Job's continuous variation plot (Fig. 13).^11^ UV-Vis-NIR absorption spectra were measured at different ratios of BC to CF_3_CO_2_H with a constant total concentration of BC + CF_3_CO_2_H = 2.00 mM (Fig. 13a). The absorbance of BC˙^+^ at 635 nm was chosen for the Job's continuous variation plot because only BC˙^+^ shows absorption at 635 nm, whereas BC, CF_3_CO_2_H, and BCH_3_^+^ do not (Fig. 3a and S7e†). The plot showed maximum absorbance at a ratio of BC : CF_3_CO_2_H = 50 : 50 (Fig. 13b), indicating that the ratio of BC and CF_3_CO_2_H is 1 : 1. According to the equation in Scheme 1a, 2/3 of the BC would be converted to BC˙^+^, giving a 67% yield. This yield was determined by a comparison between the absorbance intensity of BC˙^+^ obtained under acidic conditions (2000 mol% CF_3_CO_2_H) and the absorbance intensity of BC˙^+^ obtained under chemical oxidation conditions (NOPF_6_), using the same concentration of BC (Fig. 9b). For the chemical oxidation, in which all BC is quantitatively oxidized to BC˙^+^ by addition of NOPF_6_, the absorbance of BC˙^+^ at 635 nm was saturated at 0.76, while the absorbance observed under acidic conditions was saturated at 0.48. Thus, 0.48/0.76 = 63% BC˙^+^ generated under acidic conditions. The formation of BCH_3_^+^ was also indicated by UV-Vis-NIR spectral measurements. The spectrum of BC˙^+^ was subtracted from the spectrum of the mixture of BC˙^+^ and BCH_3_^+^ obtained from BC and 2000 mol% CF_3_CO_2_H, giving a nearly identical spectrum to that of BCH_3_^+^ (Fig. S7d–f†). The formation of BCH_3_^+^ was also confirmed by quenching the generated BC˙^+^ and BCH_3_^+^ with hydrazine or NEt_3_ (Scheme 4 and Fig. 5a). After the formation of BC˙^+^ and BCH_3_^+^ from BC and 2000 mol% CF_3_CO_2_H, quenching with 10 000 mol% hydrazine afforded BC in 68% isolated yield and BCH_2_ in 31% isolated yield. Quenching with 4000 mol% NEt_3_ afforded BC in 72% isolated yield and BCH_2_ in 18% isolated yield, with tetracarbazole **8** (Fig. 14) obtained as a by-product in ∼3% yield. These experiments clearly confirmed the disproportionation reaction in Scheme 1a. The equilibrium constant and Gibbs free energy in CH_2_Cl_2_ were determined as *K* = 1.0 × 10^9^ M^–3^ and Δ*G* = 12 kcal mol^–1^ (298 K) from the absorption spectrum of BC with 200 mol% CF_3_CO_2_H (Fig. 3a, absorbance = 0.71 at 635 nm), and *ε* = 1.5 × 10^4^ L mol^–1^ cm^–1^ for BC˙^+^ at 635 nm (Fig. 9b, NOPF_6_).

### Reversible disproportionation of tetramethyl-4,4′,10,10′-biacridine (TBA)

**Fig. 15:**
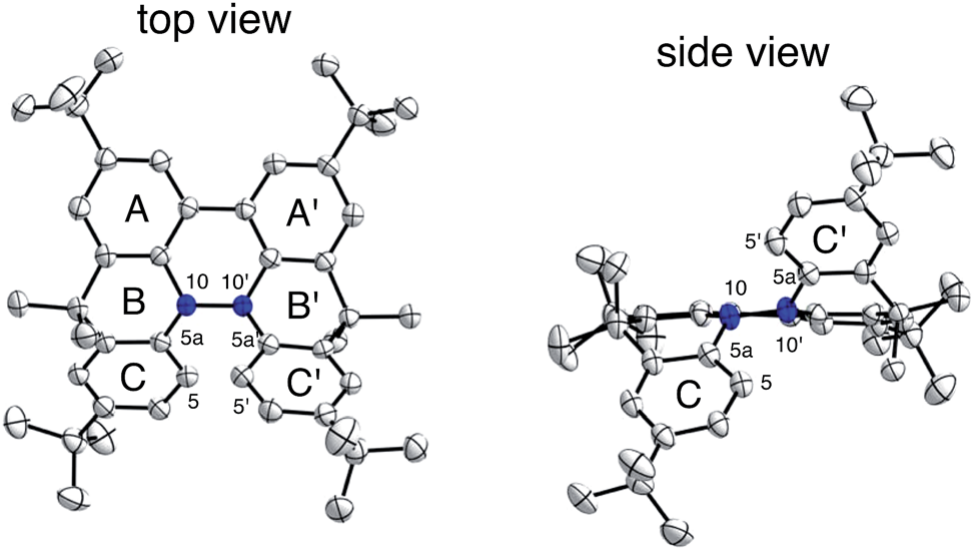
ORTEP drawings of TBA at 50% probability level obtained from X-ray crystallographic analysis. Hydrogens are omitted for clarity.

**Fig. 16:**
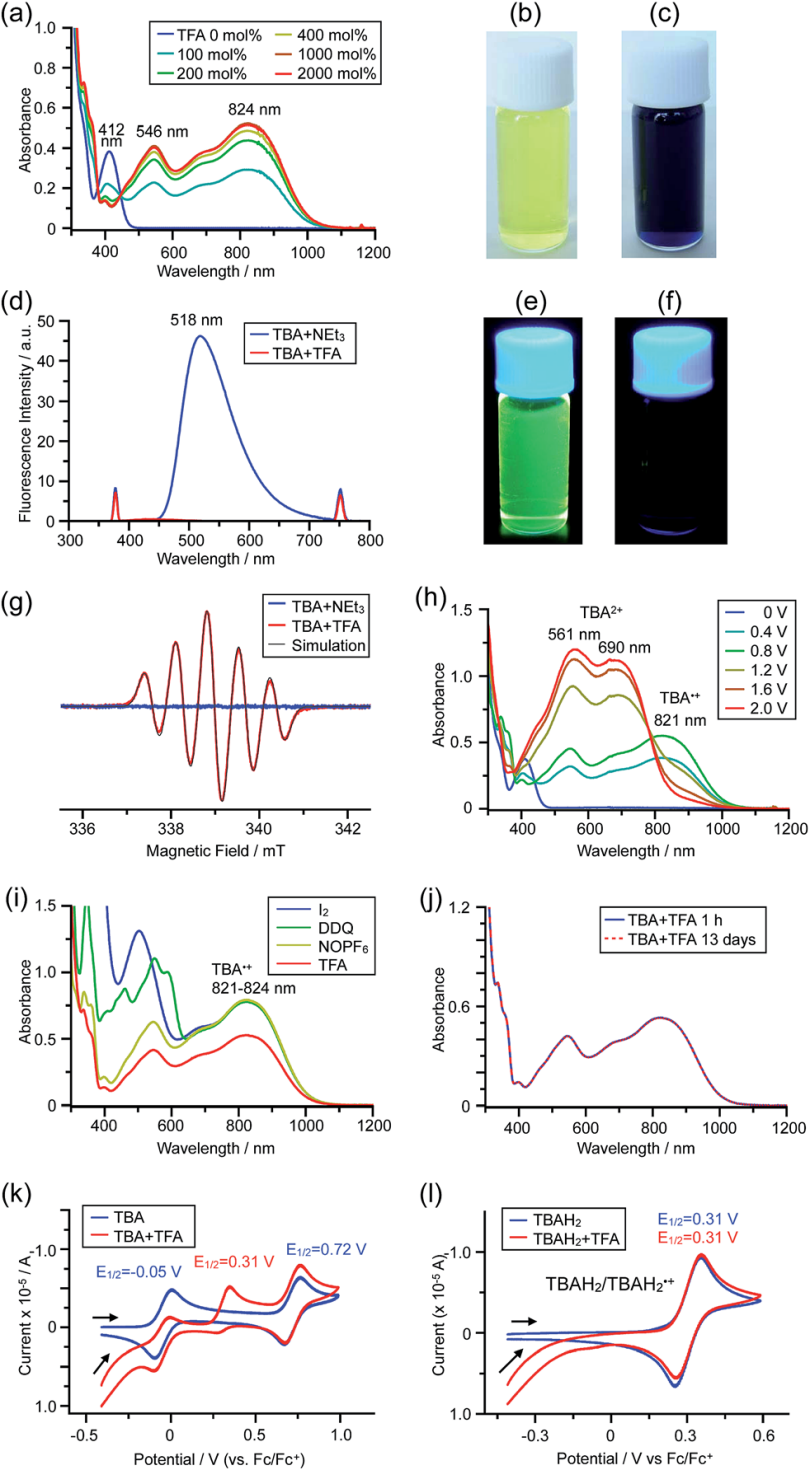
(a) Changes in the UV-Vis-NIR spectrum of TBA (0.100 mM) on addition of 0–2000 mol% CF_3_CO_2_H in CH_2_Cl_2_. (b) Photo of the solution of TBA (5.00 mM) in CH_2_Cl_2_. (c) Photo of the solution of TBA (1.00 mM) with 2000 mol% CF_3_CO_2_H in CH_2_Cl_2_. (d) Emission spectra of TBA (0.030 mM) with 500 mol% NEt_3_, and TBA with 2000 mol% CF_3_CO_2_H in CH_2_Cl_2_ (excited at 378 nm). (e) Photo of the solution of TBA (5.00 mM) in CH_2_Cl_2_ under UV light. (f) Photo of the solution of TBA (1.00 mM) with 2000 mol% CF_3_CO_2_H in CH_2_Cl_2_ under UV light. (g) ESR spectra of TBA (0.100 mM) with 1000 mol% NEt_3_, and TBA with 2000 mol% CF_3_CO_2_H in CH_2_Cl_2_ at room temperature (X-band, *ν* = 9.506032 GHz, *g* = 2.0036) and the simulated spectrum (*S* = 1/2, hfc *a* = 7.1 G with two nitrogens and 1.0, 0.5, 0.3, 0.3, and 0.2 G with 10 hydrogens). (h) UV-Vis-NIR spectra of TBA˙^+^ and TBA^2+^ generated by electrochemical oxidation (*vs.* Ag/AgCl) with a Pt electrode in 1,2-dichloroethane containing 0.1 M Bu_4_NClO_4_. (i) UV-Vis-NIR spectra of TBA˙^+^ (0.100 mM) obtained by chemical oxidation using 1000 mol% I_2_, 500 mol% DDQ in 1,2-dichloroethane, and 100 mol% NOPF_6_ in CH_2_Cl_2_, and the spectrum of TBA (0.100 mM) with 2000 mol% CF_3_CO_2_H in CH_2_Cl_2_. (j) UV-Vis-NIR spectra of TBA (0.100 mM) with 2000 mol% CF_3_CO_2_H in CH_2_Cl_2_ after 1 h and 13 days in the dark at 20 °C under air. (k) CVs of TBA and TBA with 2000 mol% CF_3_CO_2_H, and (l) CVs of TBAH_2_ and TBAH_2_ with 2000 mol% CF_3_CO_2_H in CH_2_Cl_2_ containing 0.1 M Bu_4_NClO_4_ with a Pt electrode (*vs.* Fc/Fc^+^).

**Scheme 5:**
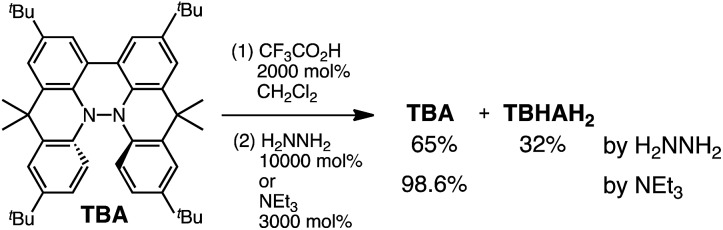
Quenching of the disproportionation of TBA.

The X-ray crystallographic analysis of TBA showed a helical molecular shape with the dihedral angle ∠C_5a_N_10_N_10′_C_5a′_ = 83° and the C_5_–C_5′_ distance = 3.61 Å (Fig. 15), which is larger than those observed in BC. TBA was found to undergo acid-responsive electron transfer disproportionation (Scheme 1b) in a similar manner to BC, and this was fully investigated by experiments and calculations. While the ^1^H NMR spectrum of TBA in freshly distilled CD_2_Cl_2_ or with 1000 mol% NEt_3_ clearly showed the signals associated with TBA, the ^1^H NMR spectrum obtained in the presence of CF_3_CO_2_H showed almost no signals due to the generation of paramagnetic radical species (Fig. S9†). The solution of TBA in CH_2_Cl_2_ showed a yellow color and green emission (Fig. 16b and e). The UV-Vis-NIR spectrum of TBA indicated that the absorption maximum in the visible region is at *λ*_max_ = 412 nm, and the emission spectrum indicated that the emission maximum is at *λ*_max_ = 518 nm (Fig. 16a and d), with a quantum yield of 17% in benzene. On treatment with CF_3_CO_2_H, the color of the solution changed to deep violet under both aerobic and anaerobic conditions (Fig. 16c). In the UV-Vis-NIR spectrum, the absorption of TBA at *λ*_max_ = 412 nm decreased, and a new broad absorption at *λ*_max_ = 824 nm corresponding to TBA˙^+^ appeared in the visible to near infrared light region (Fig. 16a and S8a†). The fluorescence also disappeared (Fig. 16d and f). The increase in the intensity of the band at 824 nm was nearly saturated on addition of 400–2000 mol% CF_3_CO_2_H. Similar changes were also observed in other organic solvents (CHCl_3_, CCl_4_, 1,2-dichloroethane, hexane, benzene, toluene, anisole) and with other weak Brønsted acids (CCl_3_CO_2_H, picric acid), as well as Lewis acids [MgBr_2_·OEt_2_, ZnCl_2_·OEt_2_], but almost no change or slight change was observed with ethyl acetate, THF, CH_3_CO_2_H, phenol, C_6_H_5_CO_2_H, LiClO_4_, and LiCl (Fig. S8b and c†). The reaction is at equilibrium under acidic conditions, as indicated by the dependence of the spectral change on the amount of acid and the concentration (Fig. 16a and S8d†). In the ESR spectral measurements, a signal corresponding to TBA˙^+^ obtained from TBA and CF_3_CO_2_H in CH_2_Cl_2_ was observed (Fig. 16g), while no signal was observed for TBA in the presence of NEt_3_. The ESR spectrum was fitted using a simulation with hfc constants *a* = 7.1 G due to coupling to two nitrogen nuclear spins, and 1.0, 0.5, 0.3, 0.3, and 0.2 G due to coupling to ten hydrogens (Fig. 16g), which agrees with TBA˙^+^ having an unpaired electron that is delocalized over the entire biacridine skeleton (Fig. S12a†). The fact that the ESR spectrum with CF_3_CO_2_D is identical to that with CF_3_CO_2_H, the lack of observation of zero-field splitting and forbidden Δ*m*_s_ = ±2 half-field transitions, and the lack of temperature dependence of the *IT* value at 5–80 K also support the doublet spin state of TBA˙^+^ (Fig. S6a and b†). The UV-Vis-NIR spectrum of TBA˙^+^ generated from TBA with CF_3_CO_2_H agreed with those of TBA˙^+^ formed by the electrochemical or chemical (I_2_, DDQ, NOPF_6_) oxidation of TBA (Fig. 16h and i), confirming that the radical species is TBA˙^+^. TBA˙^+^ also exhibited high stability under acidic conditions. The UV-Vis-NIR spectrum scarcely changed, even after 13 days in the dark at room temperature under air (Fig. 16j). A comparison between the absorbance intensity (0.53) of TBA˙^+^ obtained under acidic conditions and the absorbance intensity (0.78) of TBA˙^+^ obtained by oxidation with DDQ or I_2_ provided a yield of 68% (0.53/0.78) TBA˙^+^, which was formed by 2/3 of the TBA (Fig. 16i). The formation of TBAH_4_^2+^ was indicated by the UV-Vis-NIR spectra. Subtraction of the spectrum of TBA˙^+^ from the spectrum of the mixture of TBA˙^+^ and TBAH_4_^2+^ gave a nearly identical spectrum to that of TBAH_4_^2+^ (Fig. S8e–g†). The presence of TBAH_4_^2+^ was also observed by electrochemical analysis (CV) (Fig. 16k and l). While only the oxidation of TBA to TBA˙^+^ at *E*_1/2_ = –0.05 V and further oxidation to TBA^2+^ at 0.72 V (*vs.* Fc/Fc^+^) were observed as two reversible waves under neutral conditions, a new oxidation wave at *E*_1/2_ = 0.31 V appeared for TBA with CF_3_CO_2_H, which is consistent with the oxidation potential of TBAH_2_ at *E*_1/2_ = 0.31 V. The formation of TBAH_4_^2+^ was finally confirmed by quenching the generated TBA˙^+^ and TBAH_4_^2+^ with hydrazine, which gave TBA in 65% isolated yield and TBAH_2_ in 32% isolated yield (Scheme 5).

**Fig. 17:**
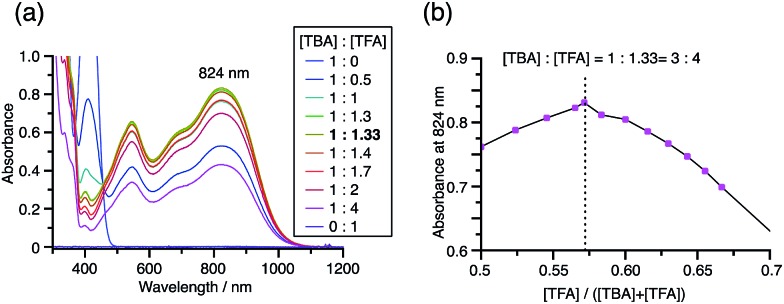
(a) Continuous variation of the UV-Vis-NIR spectra in CH_2_Cl_2_ on changing the TBA/CF_3_CO_2_H ratio. The concentration of TBA + CF_3_CO_2_H is 4.00 mM and a 1 mm cell was used. (b) Continuous variation plot of the absorbance at 824 nm *versus* the TBA/CF_3_CO_2_H ratios from (a).

**Fig. 18:**
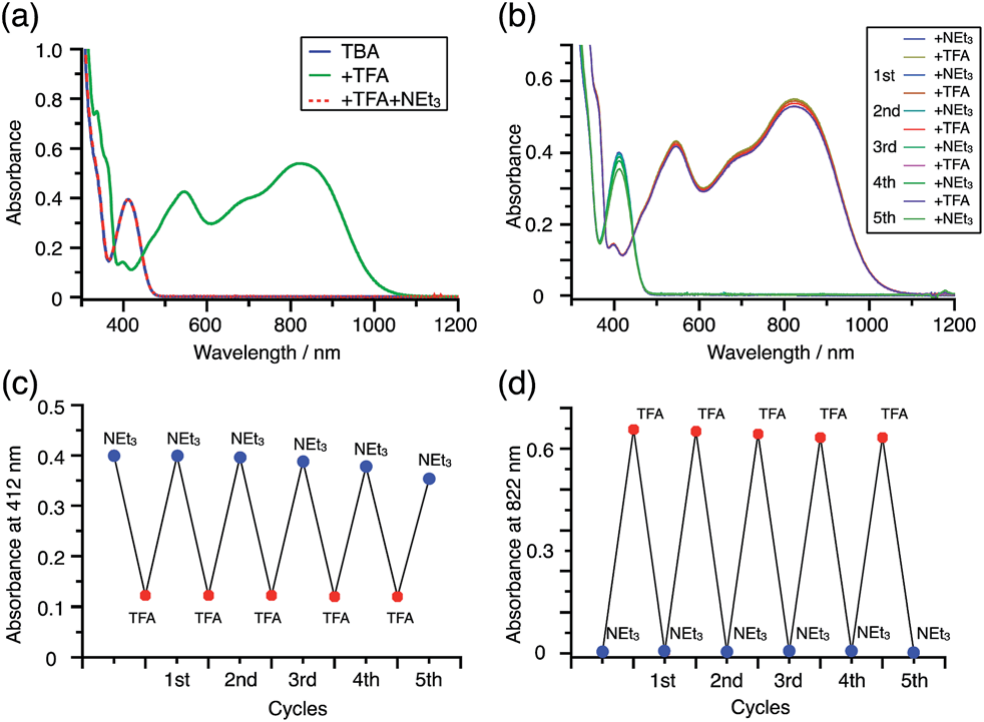
(a) UV-Vis-NIR spectra of TBA (0.100 mM), TBA with 2000 mol% CF_3_CO_2_H, and TBA with 2000 mol% CF_3_CO_2_H followed by addition of 3000 mol% NEt_3_ in CH_2_Cl_2_. (b) UV-Vis-NIR spectra during repeated acidification/neutralization by addition of CF_3_CO_2_H and NEt_3_ to TBA (0.100 mM) in CH_2_Cl_2_ for up to five cycles. (c) Absorbance at 412 nm and (d) absorbance at 822 nm for the UV-Vis-NIR spectra in (b) during repeated acidification/neutralization by addition of CF_3_CO_2_H and NEt_3_ to TBA in CH_2_Cl_2_ for up to five cycles.

While the reaction of TBA is similar to that of BC, three informative differences were observed. One difference is the reaction stoichiometry of TBA and CF_3_CO_2_H (Scheme 1). The Job's continuous variation plot (Fig. 17 and S8h†) provided a ratio of TBA : CF_3_CO_2_H of 3 : 4, while the ratio of BC : CF_3_CO_2_H is 3 : 3 (Fig. 13). Thus, the reduced product TBAH_2_ is concluded to be di-protonated, whereas BCH_2_ is mono-protonated (Scheme 1). This difference is attributed to the greater basicity of dimethylacridine than carbazole. The equilibrium constant and Gibbs free energy in CH_2_Cl_2_ were determined as *K* = 6.5 × 10^17^ M^–4^ and Δ*G* = 24 kcal mol^–1^ (298 K) from the absorption spectrum of TBA with 100 mol% CF_3_CO_2_H (Fig. 16a, absorbance = 0.29 at 824 nm), and *ε* = 7.9 × 10^3^ L mol^–1^ cm^–1^ for TBA˙^+^ at 824 nm (Fig. 16i, NOPF_6_). The second difference is the remarkable reversibility of the reaction of TBA on neutralization with NEt_3_. Upon quenching with 4000 mol% NEt_3_ after the conversion of BC to BC˙^+^CF_3_CO_2_^–^ and BCH_3_^+^2CF_3_CO_2_^–^ on addition of 2000 mol% CF_3_CO_2_H in CH_2_Cl_2_, BC was recovered in 72% yield, concomitant with BCH_2_ in 18% yield (Fig. 5a and Scheme 4). In contrast, addition of NEt_3_ recovered TBA in 98.6% yield based on the absorption intensity at 412 nm (Scheme 5, Fig. 18a and Table S2†). ^1^H NMR experiments also demonstrated the high recovery of TBA (Fig. S9†). This remarkably high reversibility was confirmed for up to five cycles by UV-Vis-NIR spectral measurements during repeated acidification/neutralization on addition of CF_3_CO_2_H (∼94 000 mol%) and NEt_3_ (∼120 000 mol%) to TBA, giving an average of 97.6% recovery yield per cycle based on the absorbance at 412 nm (Fig. 18b–d). The difference in the reversibility was attributed to the difference in the redox potentials of BC and TBA. The third difference is in the reaction mechanism and relates to the reaction order during the N–N bond cleavage step. The differences in the reversibility of the reaction and the reaction order are discussed in the following sections.

### Reaction mechanism of disproportionation

**Scheme 6:**
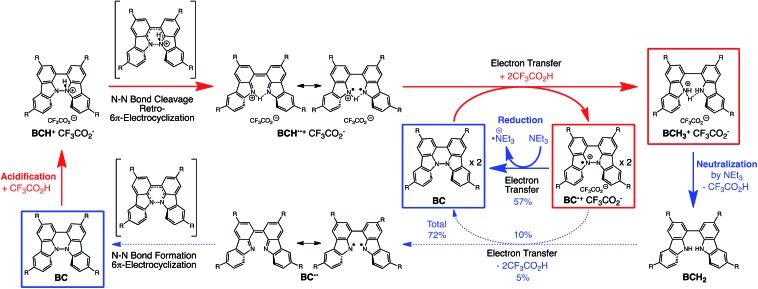
Proposed reaction mechanism for the disproportionation of BC.^12^

**Scheme 7:**
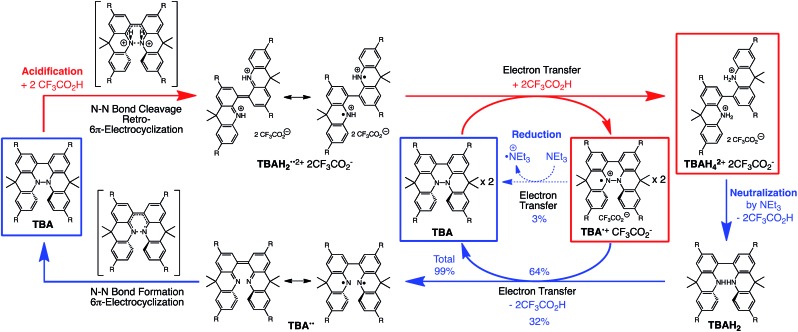
Proposed reaction mechanism for the disproportionation of TBA.^12^

**Fig. 19:**
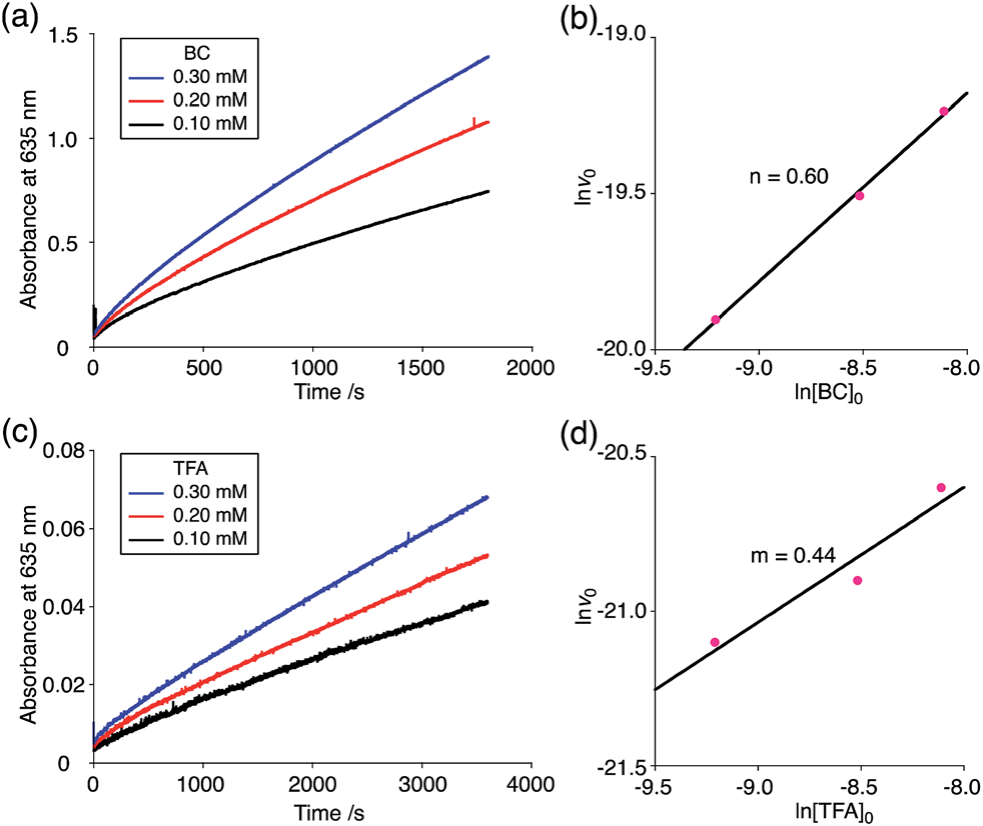
(a) Time-dependent change in the absorbance of BC˙^+^ at 635 nm on mixing BC (0.10, 0.20, and 0.30 mM) and TFA (5.00 mM) in CH_2_Cl_2_ at 20 °C. (b) The reaction order with respect to BC obtained from a plot of ln[BC]_0_*versus* ln *v*_0_ using the data in (a). (c) Time-dependent change in the absorbance of BC˙^+^ at 635 nm on mixing BC (5.00 mM) and TFA (0.10, 0.20, and 0.30 mM) in CH_2_Cl_2_ at 20 °C. (d) The reaction order with respect to TFA obtained from a plot of ln[TFA]_0_*versus* ln *v*_0_ using the data in (c).

**Scheme 8:**
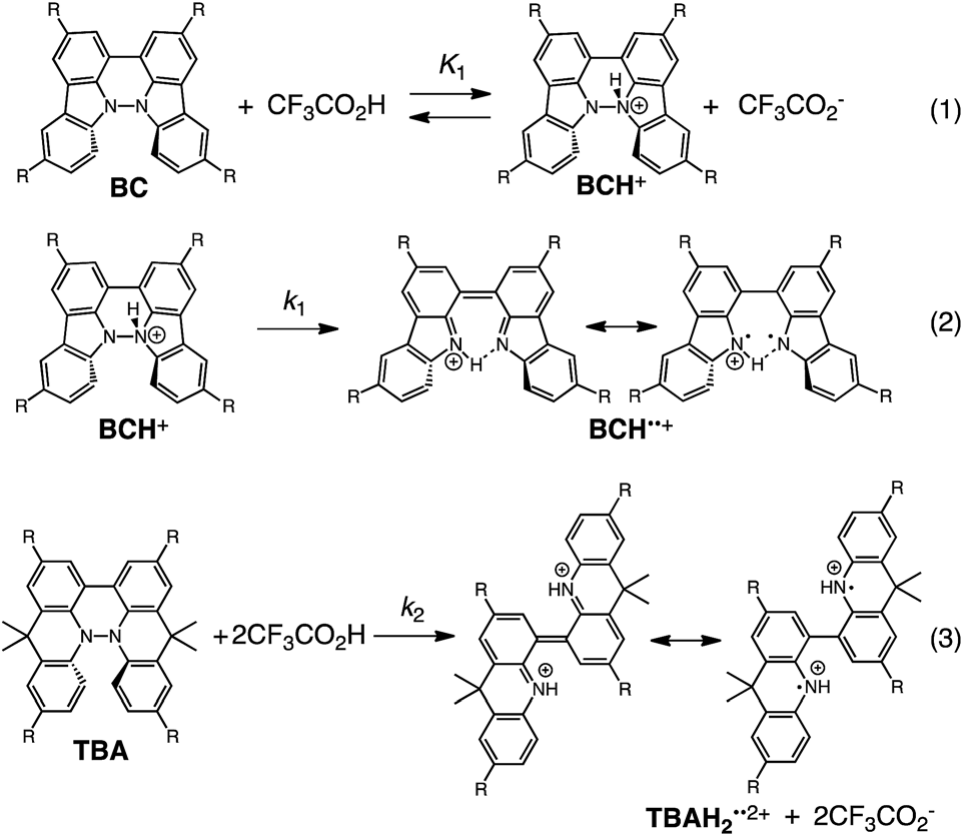
Proposed reaction mechanisms of the N–N bond cleavage steps for BC and TBA.^12^

The proposed mechanisms of the electron transfer disproportionation reactions of BC and TBA are shown in Schemes 6 and 7,^12^ and involve acid-triggered N–N bond cleavage reactions to generate electrophilic open-shell singlet biradical species followed by electron transfer. Upon acidification, BC and TBA undergo protonation by CF_3_CO_2_H, followed by a thermal retro-6π-electrocyclization^13^ to cleave the N–N bond, giving the open-shell singlet biradical intermediate BCH˙˙^+^ or TBAH_2_˙˙^2+^. Electron transfer from the electron-rich BC or TBA to the electron-deficient BCH˙˙^+^ or TBAH_2_˙˙^2+^ and protonation afford one equivalent of BCH_3_^+^ or TBAH_4_^2+^ and two equivalents of BC˙^+^ or TBA˙^+^, respectively. The differences in the N–N bond cleavage steps, *i.e.* the mono-protonation mechanism of BC and the di-protonation mechanism of TBA, are proposed based on the results of kinetic experiments. Kinetic studies of the reaction of BC with different concentrations of CF_3_CO_2_H using the initial rates method (Fig. 19 and S10 and Tables S4 and 5†) showed that the reaction orders with respect to BC and CF_3_CO_2_H are 0.6 and 0.4, respectively, in both CH_2_Cl_2_ and benzene. These reaction orders can be explained by the reaction model in Scheme 8: (1) BC and CF_3_CO_2_H are in equilibrium with BCH^+^ and CF_3_CO_2_^–^ with an equilibrium constant *K*_1_ = [BCH^+^][CF_3_CO_2_^–^]/[BC][CF_3_CO_2_H], and (2) the intermediate BCH^+^ undergoes an N–N bond cleavage reaction. With this model, the reaction rate *v* is 1st order with respect to BCH^+^.*v* = *k*_1_[BCH^+^]

Under the condition of [BCH^+^] = [CF_3_CO_2_^–^], [BCH^+^] is given as:[BCH^+^] = *K*_1_^1/2^[BC]^1/2^[CF_3_CO_2_H]^1/2^

Thus, the reaction rate is 0.5 order with respect to both BC and CF_3_CO_2_H, which is consistent with the observed reaction orders.*v* = *k*_1_*K*_1_^1/2^[BC]^1/2^[CF_3_CO_2_H]^1/2^

**Fig. 20:**
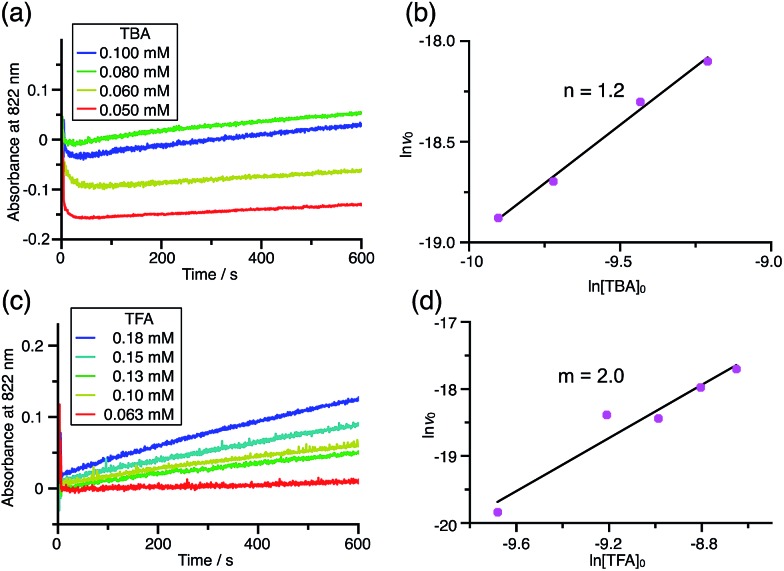
(a) Time-dependent change in the absorbance of TBA˙^+^ at 822 nm on mixing TBA (0.100, 0.080, 0.060, and 0.050 mM) and TFA (2.00 mM) in CH_2_Cl_2_ at –70 °C. (b) The reaction order with respect to TBA obtained from a plot of ln[TBA]_0_*versus* ln *v*_0_ using the data in (a). (c) Time-dependent change in the absorbance of TBA˙^+^ at 822 nm on mixing TBA (5.00 mM) and TFA (0.18, 0.15, 0.13, 0.10, and 0.063 mM) in CH_2_Cl_2_ at –60 °C. (d) The reaction order with respect to TFA obtained from a plot of ln[TFA]_0_*versus* ln *v*_0_ using the data in (c).

Using the reaction model, the activation energy barrier of BC in CH_2_Cl_2_ was determined to be Δ*G*^‡^ = 28 kcal mol^–1^ (293 K) (Tables S4 and 5†). On the other hand, the kinetic experiments for TBA showed that the reaction is 1st order with respect to TBA and 2nd order with respect to CF_3_CO_2_H (Fig. 20 and Tables S6 and 7†), indicating that di-protonation of TBA precedes the N–N bond cleavage reaction [Scheme 8(3)]. The fact that the N–N bond cleavage reaction of TBA occurs after di-protonation rather than mono-protonation is consistent with the greater basicity of TBA than that of BC. The activation energy barrier of TBA in CH_2_Cl_2_ was determined to be Δ*G*^‡^ = 11 kcal mol^–1^ (293 K) (Fig. S11 and Table S8†). This value is much lower than the 28 kcal mol^–1^ of BC, showing that the reaction of TBA with CF_3_CO_2_H is much faster than that of BC. In the case of BC, the di-protonation pathway would be disfavored compared with mono-protonation, due to the lower basicity. The results of the kinetic experiments also confirm that the N–N bond cleavage of BCH^+^ to form BCH˙˙^+^, or of TBAH_2_^2+^ to form TBAH_2_˙˙^2+^, is the rate-determining step, and that the bimolecular electron transfer reaction from BC to BCH^+^ or from TBA to TBAH_2_^2+^ is not the rate-determining step, because the reaction would be 2nd order with respect to BC or TBA in the implausible latter mechanism.

**Fig. 21:**
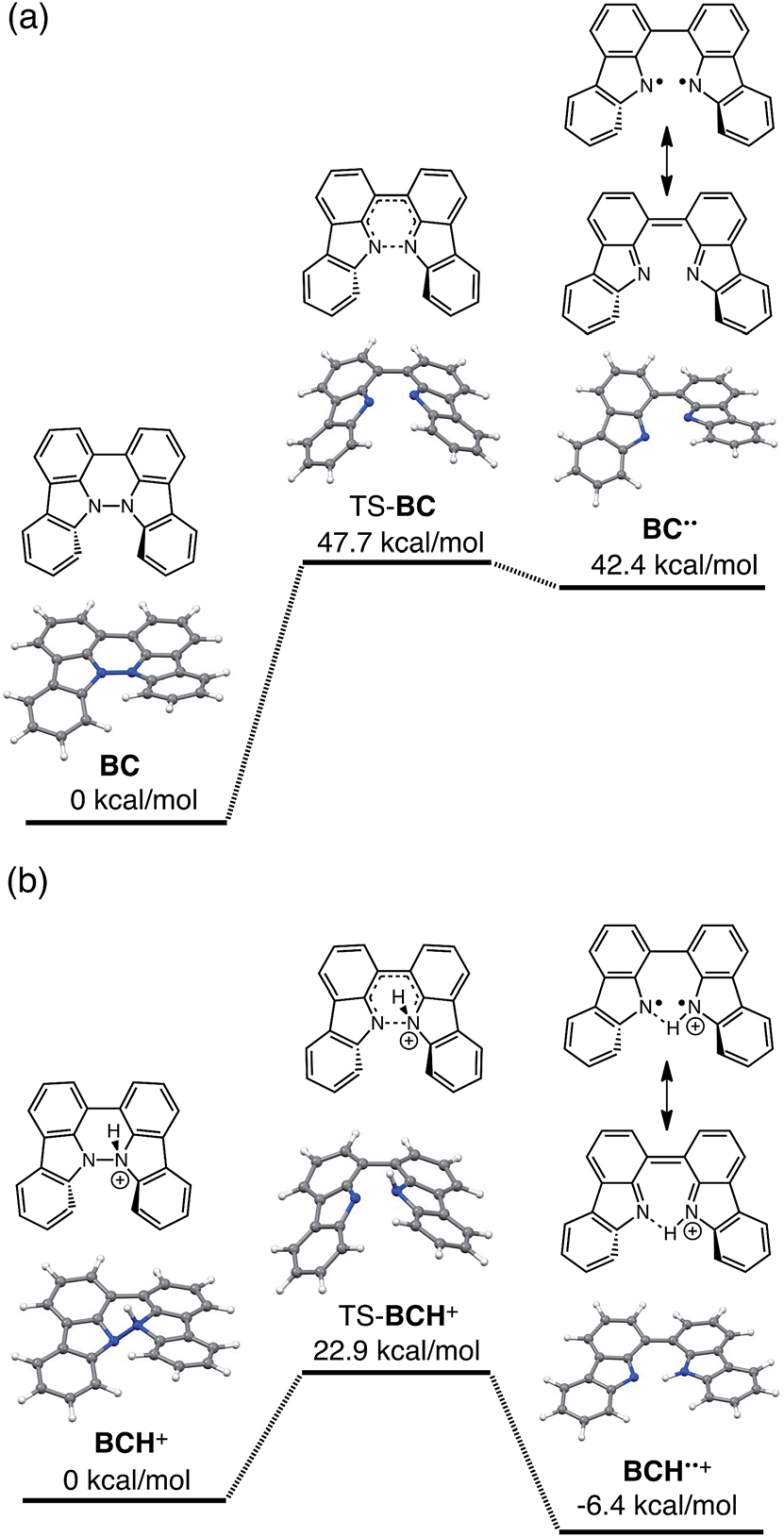
DFT calculations of the N–N bond cleavage/formation reactions of BC [UωB97XD/6-31G(d)]. (a) Neutral conditions. (b) Mono-protonated conditions. *t*-Butyl groups are omitted for the calculations.

**Fig. 22:**
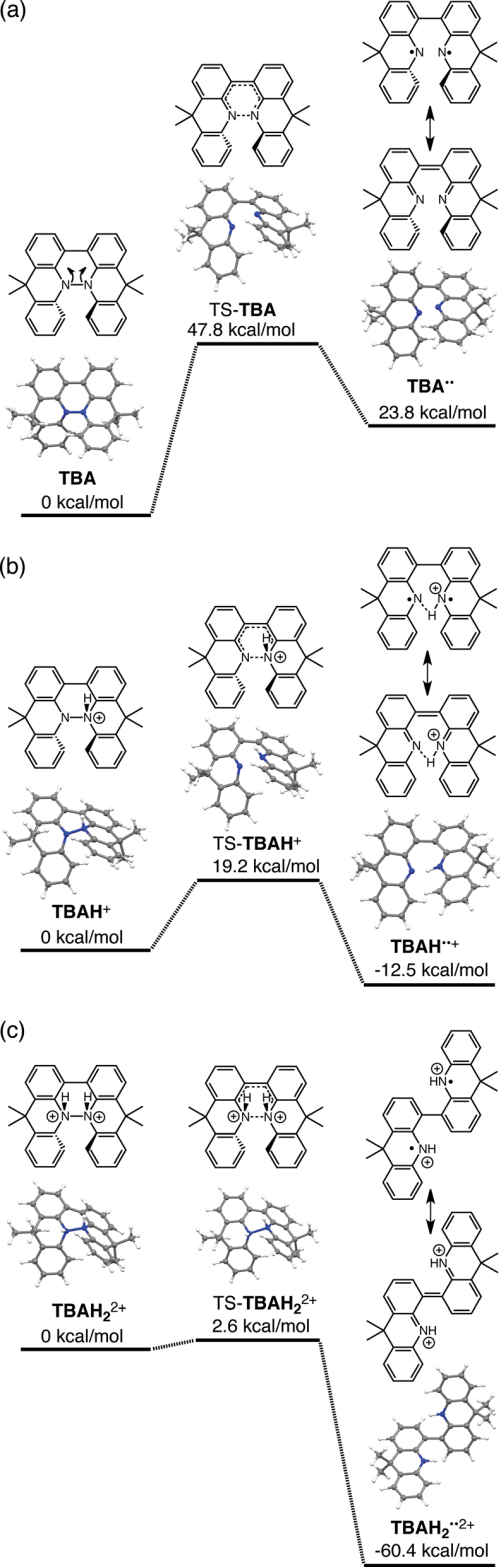
DFT calculations of the N–N bond cleavage/formation reactions of TBA [UωB97XD/6-31G(d)]. (a) Neutral conditions. (b) Mono-protonated conditions. (c) Di-protonated conditions. *t*-Butyl groups are omitted for the calculations.

The reaction mode of the N–N bond cleavage reactions must be the thermally allowed disrotatory retro-6π-electrocyclization.^13^ The N–N bond cleavage reaction processes under neutral and acidic conditions were further examined by density functional theory (DFT) calculations [UωB97XD/6-31G(d)] in order to confirm this (Fig. 21 and 22).^12^ Under neutral conditions (Fig. 21a and 22a), the activation energy for the disrotatory retro-6π-electrocyclization is very high (TS-BC: 47.7 kcal mol^–1^, TS-TBA: 47.8 kcal mol^–1^), and BC and TBA are thermodynamically much more stable than the open-shell singlet biradical states^14^ (BC˙˙: 42.4 kcal mol^–1^, TBA˙˙: 23.8 kcal mol^–1^) formed after N–N bond cleavage. Thus, the N–N bond cleavage reactions do not take place under neutral conditions. In contrast, after mono-protonation of BC under acidic conditions (Fig. 21b), the calculated energy barrier between the mono-protonated BCH^+^ and the transition state TS-BCH^+^ becomes lower (+22.9 kcal mol^–1^), and the open-shell singlet state^14^ BCH˙˙^+^ becomes more stable (–6.4 kcal mol^–1^) than BCH^+^. This calculated energy barrier is consistent with the experimental value (Δ*G*^‡^ = 28 kcal mol^–1^) after accounting for the energy required to protonate BC to form BCH^+^, which demonstrates the high feasibility of the N–N bond cleavage reaction under acidic conditions. The value of the energy barrier between BCH^+^ and TS-BCH^+^ also supports the proposed reaction model, in which BCH^+^ is the intermediate [Schemes 6 and 8(1) and (2)]. Both mono-protonation and di-protonation mechanisms were calculated for TBA under acidic conditions (Fig. 22b and c). In the case of di-protonation, *cis*-di-protonation of TBA is necessary for the thermal disrotatory retro-6π-electrocyclization. The calculated energy barriers between mono-protonated TBAH^+^ and TS-TBAH^+^, and between *cis*-di-protonated TBAH_2_^2+^ and TS-TBAH_2_^2+^ also become lower (TS-TBAH^+^: +19.2 kcal mol^–1^, TS-TBAH_2_^2+^: +2.6 kcal mol^–1^), and the open-shell singlet states^14^ were more stable (TBAH˙˙^+^: –12.5 kcal mol^–1^, TBAH_2_˙˙^2+^: –60.4 kcal mol^–1^), supporting the N–N bond cleavage reaction under acidic conditions. After accounting for the protonation energy, the di-protonation mechanism is consistent with the experimental energy barrier (Δ*G*^‡^ = 11 kcal mol^–1^). Although the energy barrier of the mono-protonation mechanism is also low enough for the reaction of TBA to proceed, the calculated values indicate that the di-protonation mechanism is faster than the mono-protonation mechanism. The experimental reaction orders and the relatively small calculated barrier (+2.6 kcal mol^–1^) between TBAH_2_^2+^ and TS-TBAH_2_^2+^ suggest that TBAH_2_^2+^ is not an intermediate, and that the *cis*-di-protonation of TBA is followed by the N–N bond cleavage reaction without the formation of an intermediate [Schemes 7 and 8(3)]. After the formation of the electron-deficient BCH˙˙^+^ or TBAH_2_˙˙^2+^, electron transfer from the electron-rich BC or TBA takes place (Schemes 6 and 7). The results of the electrochemical analysis (Fig. 10a and b and 16k and l and Table 1) indicate that the lower oxidation potentials of BC (0.17 V) and TBA (–0.05 V) compared with those of BCH (0.74 V) and TBAH_2_ (0.31 V) promote electron transfer from BC to BCH˙˙^+^ and from TBA to TBAH_2_˙˙^2+^. Protonation to form BCH_3_^+^ or TBAH_4_^2+^ also assists the electron transfer process under equilibrium under acidic conditions (Schemes 6 and 7). The observation of no NMR signals corresponding to BC and BCH_3_^+^ or TBA and TBAH_4_^2+^, even in the presence of small amounts of acid, indicates that the electron transfer is under fast equilibrium.

**Table 1:** Electrochemical oxidation potentials^*a*^

BC	BCH	TBA	TBAH_2_	NEt_3_
0.17 V	0.74^*b*^ V	–0.05 V	0.31 V	0.45^*b*^ V

^*a*^In CH_2_Cl_2_ containing 0.1 M Bu_4_NClO_4_ (*vs.* Fc/Fc^+^).

^*b*^Differential pulse voltammetry (DPV).

### Reaction mechanism of the reversible disproportionation

**Fig. 23:**
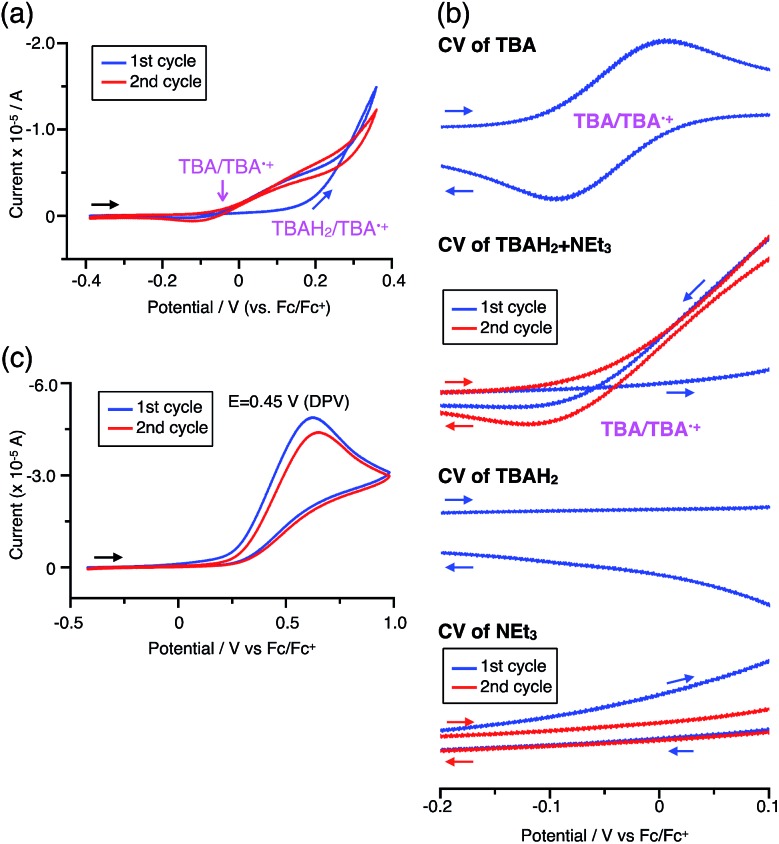
(a) CV of TBAH_2_ with 1000 mol% NEt_3_ in CH_2_Cl_2_ containing 0.1 M Bu_4_NClO_4_ (*vs.* Fc/Fc^+^). (b) Expanded views of the CVs of TBA (Fig. 16k), TBAH_2_ + NEt_3_ (a), TBAH_2_ (Fig. 16l), and NEt_3_ (c) (*vs.* Fc/Fc^+^). (c) CV of NEt_3_ in CH_2_Cl_2_ containing 0.1 M Bu_4_NClO_4_ (*vs.* Fc/Fc^+^).

The proposed mechanisms for the recovery of BC and TBA on treatment with NEt_3_ after the disproportionation are shown in Schemes 6 and 7. The reversible reaction of TBA features back electron transfer and N–N bond formation enabled by the acid-regulated N–N bond cleavage/formation reactions, which provide an efficient switching mechanism, and the balance of the redox potentials. The difference in the reversibility of the reactions of BC and TBA can be explained by the difference in the redox potentials *versus* that of NEt_3_ (Table 1). Theoretically, 67% BC^+^ and 33% BCH_3_^+^, or 67% TBA^+^ and 33% TBAH_4_^2+^, are formed through disproportionation on acidification of BC or TBA. When the mixture was quenched with hydrazine, BC˙^+^ and TBA˙^+^ were reduced by electron transfer from hydrazine, and BCH_3_^+^ and TBAH_4_^2+^ were neutralized by hydrazine almost simultaneously. Thus, the recovered products (68% BC and 31% BCH_2_, or 65% TBA and 32% TBAH_2_) reflected the ratio of the disproportionation products formed in the reaction (Schemes 4 and 5). On the other hand, when the mixture obtained from TBA was quenched with NEt_3_, TBA was recovered in 99% yield (Fig. 18a and Scheme 5). This result indicates that NEt_3_ acts only as a base to neutralize TBAH_4_^2+^ to TBAH_2_, and that TBAH_2_ is converted back to TBA through back electron transfer between TBA˙^+^, deprotonation by NEt_3_, and the N–N bond forming 6π-electrocyclization (Scheme 7). The formation of TBA from TBAH_2_ was reproduced by electrochemical oxidation in the presence of NEt_3_. Whereas the electrochemical oxidation (CV) of TBAH_2_ with sweeping up to 0.59 V in the absence of NEt_3_ showed only a reversible oxidation wave corresponding to TBAH_2_/TBAH_2_˙^+^ (Fig. 16l), the oxidation in the presence of NEt_3_ with sweeping up to 0.36 V converted TBAH_2_ to TBA˙^+^ through the electrochemical oxidation and deprotonation of TBAH_2_ by NEt_3_ to form TBA˙˙, the thermal disrotatory 6π-electrocyclization of TBA˙˙ to form TBA, and the electrochemical oxidation of TBA to form TBA˙^+^, as indicated by the observation of the TBA˙^+^/TBA redox wave (Fig. 23a and b). The oxidation potential of TBAH_2_ of 0.31 V is lower than the 0.45 V of NEt_3_ (Table 1 and Fig. 16l and 23c), which indicates that TBAH_2_ is a stronger electron donor than NEt_3_, and is better able to reduce TBA˙^+^ to TBA. Based on the total recovery yield (99%) of TBA, 64% of TBA˙^+^ out of the theoretically formed 67% TBA˙^+^ would be reduced by 32% TBAH_2_ out of the 33% TBAH_2_ formed, while 3% of TBA˙^+^ would be reduced by NEt_3_ (Scheme 7). Judging by the oxidation potentials of TBA (–0.05 V) and TBAH_2_ (0.31 V), the back electron transfer process between TBA˙^+^ and TBAH_2_ is unfavorable. Nevertheless, these redox potentials are close enough for the back electron transfer to proceed under equilibrium to form TBA˙˙, aided by deprotonation by NEt_3_. To complete this unfavorable equilibrium, the N–N bond forming 6π-electrocyclization plays an important role. Based on the DFT calculations (Fig. 22a), the N–N bond formation reaction of TBA˙˙ to form TBA *via* TS-TBA under neutral conditions is likely to proceed irreversibly, judging by the low activation energy (24.0 kcal mol^–1^ for the transformation of TBA˙˙ into TS-TBA) and the thermodynamic stability of TBA (–23.8 kcal mol^–1^*vs.* TBA˙˙). Thus, the irreversible formation of stable TBA from TBA˙˙ through the N–N bond formation reaction would shift the equilibrium and complete the reverse reaction (Scheme 7). In contrast, when the disproportionation mixture obtained from BC was quenched with NEt_3_, only a slightly higher yield (72%) of BC and a lower yield (18%) of BCH_2_, compared with the yields obtained with hydrazine, were recovered (Fig. 5a and Scheme 4). Based on the recovery yield of BC (72%), only 10% of BC˙^+^ out of the theoretically formed 67% BC˙^+^ would be reduced by only 5% BCH_2_ out of the 33% BCH_2_ formed, while most (57%) of the BC˙^+^ would be reduced by NEt_3_ (Scheme 6). The oxidation potential (0.74 V) of BCH_2_ is far higher than the 0.17 V of BC and is also higher than the 0.45 V of NEt_3_. Thus, NEt_3_ acts as the main electron donor to reduce BC˙^+^, and only some of the BCH_2_ reduces BC˙^+^ (Scheme 6). These mechanisms indicate that the disproportionation of TBA is really reversible by neutralization, but that of BC is not, although a moderate yield (72%) of BC is recovered. The recovery of BC is mostly attributed to another reaction that reproduces BC, *i.e.* reduction by NEt_3_.

### Related N–N linked polyheterocyclic compounds

**Fig. 24:**
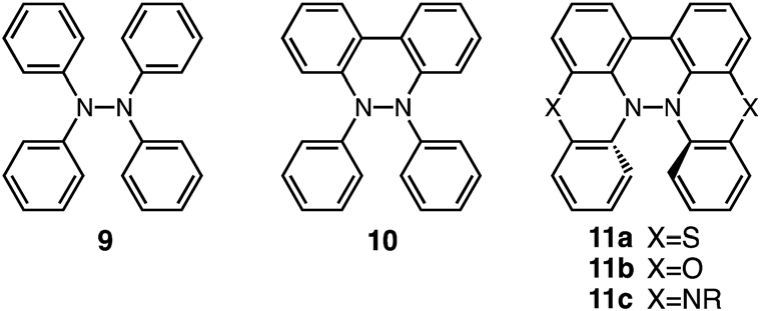
Tetraphenylhydrazine **9**, 9,10-dihydro-9,10-diphenylphenanthroline **10**, biphenothiazine **11a**, biphenoxazine **11b**, and biphenazine **11c**.

Tetraphenylhydrazine **9** (Fig. 24) was reported to undergo acid-promoted homolytic N–N bond cleavage under strong acidic conditions (HCl, H_2_SO_4_) in the early 1900s.^15^ However, the generated aminium radical is unstable and forms decomposition products in this case. While the syntheses and some properties of 9,10-dihydro-9,10-diphenylphenanthroline **10** (ref. 16) and biphenothiazine **11a** (ref. 17) (Fig. 24) were reported, their reactivities toward acids were not investigated. Judging by the similarity of their structures to those of BC and TBA, compounds **11**, which share “a hydrazinohelicene structure” with BC and TBA (Fig. 24), would be promising substrates to undergo acid-triggered electron transfer disproportionation. Furthermore, “the diarylphenanthroline structure” of compound **10**, which is also present in BC, TBA, and **11**, could be the required minimum structure for the reaction. In order to reveal the scope and limitation of acid/base-regulated electron transfer disproportionation, the reactivity of related N–N linked polyheterocyclic compounds should be investigated, and this is now in progress.

## Conclusions

After the elucidation of the overall reactions of BC and TBA, including the detailed mechanisms, it was recognized that the characteristic chemical and physical properties of the components involved in these reactions and the external acid/base stimuli are flawlessly associated with each other in order to establish these electron transfer reactions. Specifically, the acid-regulated N–N bond cleavage/formation reactions of TBA act as an efficient switch, even with weak acids, and the balanced redox potentials of the components establish the highly reversible electron transfer reaction. Importantly, this discovery is not limited to just the electron transfer disproportionation of two organic molecules, but it also provides new design concepts for acid/base-regulated organic electron transfer systems, reducing/oxidizing chemical reagents, and functional organic materials. The notable chemical and physical properties of BC and TBA, such as their excellent multi-electron redox properties and the acid-induced radical formation, are promising for wide applications.

## Supplementary Material

Supplementary information

Crystal structure data
