# Supplementary material for: Acid/base-regulated reversible electron transfer disproportionation of N–N linked bicarbazole and biacridine derivatives
Source: Chem Sci. 2015 May 21;6(7):4160–73. doi: 10.1039/c5sc00946d (PMC5707497; doi:10.1039/c5sc00946d)
Supplement: Supplementary file 1 [file SC-006-C5SC00946D-s001.pdf]

Electronic Supplementary Material (ESI) for Chemical Science.
This journal is © The Royal Society of Chemistry 2015

Table of Contents

**1. General Information**	**S2**
**2. Synthesis and Characterization Data of Compounds**	**S2**
**3. ^{1}H and ^{13}C NMR Spectra**	**S8**
**4. Solid State NMR Analysis**	**S19**
**5. X-ray Crystallographic Analysis**	**S20**
**6. ESR Spectral Measurements**	**S20**
**7. UV-Vis-NIR Spectral Experiments**	**S21**
**8. Fluorescence Spectral Experiments**	**S24**
**9. Kinetic Experiments**	**S25**
**10. Theoretical Calculation**	**S29**
**11. Scheme and Figures**	**S31**
**11. References**	**S42**

1. General Information

Melting points were determined on a Stanford Research Systems MPA100 or a Yanaco micro melting point apparatus MP-J3 and were uncorrected. IR spectra were recorded on a JASCO FT IR-4100 spectrometer by KBr pellet or a JASCO FT/IR-460plus spectrometer equipped with ATR PRO 400-S in ATR mode using ZnSe crystal plate. UV-Vis-NIR spectra were recorded on a JASCO V-670 spectrometer or SHIMADZU UV-3600, UV-2550 spectrometers with 1 cm quartz cell unless otherwise noted. Fluorescence spectra were recorded on a JASCO FP6500 spectrometer or SHIMADZU RF-5300PC spectrofluorophotometer. Solution state ^{1}H and ^{13}C NMR spectra were measured on a JEOL JNM-ECS400 spectrometer at 23 °C at 400 and 100 MHz, respectively. $CDCl_3$ and CD_2Cl_2 were used as a solvent and the residual solvent peaks were used as an internal standard (^{1}H NMR: $CDCl_3$ 7.26 ppm, CD_2Cl_2 5.30 ppm; ^{13}C NMR: $CDCl_3$ 77.0 ppm, CD_2Cl_2 53.5 ppm). Solid state NMR spectra were carried out using a BrukerAvance 600 spectrometer equipped with 2.5 mm O.D. ^{1}H-^{13}C-^{15}N triple resonance MAS probe. High resolution (HR) and low resolution (LR) mass spectra (MS) were measured on a JEOL JMS-777V spectrometer using electron impact ionization method (EI) and a Applied Biosystems Voyager DE-STR spectrometer using matrix assisted laser desorption ionization time-of-flight method (MALDI-TOF). CW and pulse ESR spectra were measured on Bruker EMX plus, Bruker E500, and Bruker E680 spectrometers. Heating reactions were carried out by EYELA organic synthesizers. Medium pressure liquid chromatography (MPLC) was performed on Kanto 60N silica gel using a preparative medium pressure liquid chromatography system. TLC analysis was performed using Merck Silica gel 60 F_{254} and PTLC was conducted using Wako Wakogel B-5F. Gel permeation chromatography (GPC) was performed on JAIGEL 1H and 2H using JAI Recycling Preparative HPLC system and $CHCl_3$ as the solvent. Reagents and solvents for syntheses were commercially purchased and air and/or moisture sensitive reactions were carried out under an argon atmosphere. The solvents (CH_2Cl_2, CD_2Cl_2, benzene etc.) for the analyses of acid-sensitive BC and TBA were freshly distilled before use according to the standard methods.

2. Synthesis and Characterization Data of Compounds

1-Bromo-3,6-di-*tert*-butylcarbazole (**2**)[1] and 2,7-di-*tert*-butyl-9,10-dihydro-9,9-dimethylacridine (**5**)[2] were prepared from 3,6-di-*tert*-butylcarbazole (**1**) and 9,10-dihydro-9,9-dimethylacridine by the reported procedures, respectively.

1,1'-Dibromo-3,3',6,6'-tetra-*tert*-butyl-9,9'-bicarbazole (3)

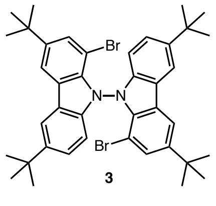

3

To a solution of 1-bromo-3,6-di-*tert*-butylcarbazole **2** (5.00 g, 14.0 mmol) in acetone (130 mL) was added $KMnO_4$ (5.50 g, 34.9 mmol) at room temperature. The reaction mixture was warmed up to 60 °C with a reflux condenser. The reaction was stirred for 4 h at 60 °C, and cooled down to room temperature. After evaporation of acetone under reduced pressure, the reaction mixture was suspended in $CHCl_3$, filtered through a glass sintered funnel, and washed by $CHCl_3$. The filtrate was washed by saturated aqueous $Na_2S_2O_3$ solution (30 mL×2), brine

(30 mL×1), dried over Na_2SO_4, filtered, and concentrated under reduced pressure. The residue was purified by recrystallization from hexane to give **3** (3.74 g, 75% yield).

Mp: 239 °C (decomp.). IR (KBr): ν 3053, 2958, 2867, 1742, 1605, 1555, 1486, 1460, 1445, 1388, 1361, 1293, 1259, 1224, 1194, 1106, 1087, 1050 cm^{-1}. ^1H NMR ($CDCl_3$): δ 8.11 (2H, d, J = 1.6 Hz), 8.10 (2H, d, J = 1.6 Hz), 7.54 (2H, d, J = 1.6 Hz), 7.34 (2H, dd, J = 8.8, 1.6Hz), 6.66 (2H, d, J = 8.8 Hz), 1.44 (18H, s), 1.41 (18H, s) ppm. ^{13}C NMR ($CDCl_3$): δ 145.4, 144.6, 140.3, 135.7, 128.8, 125.1, 124.6, 120.6, 116.3, 115.8, 108.8, 102.6, 34.8, 32.0, 31.9 ppm. HRMS (m/z) for $C_{40}H_{46}Br_2N_2$ (M^+): Calculated 712.2028, found 712.2035.

3,3',6,6'-Tetra-*tert*-butyl-1,1',9,9'-bicarbazole (BC) from 3

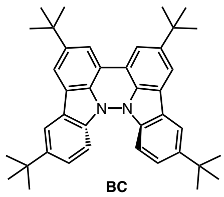

BC

A solution of 2,2'-bipyridyl (329 mg, 2.11 mmol), 1,5-cyclooctadiene (257 μL, 2.10 mmol), and bis(1,5-cyclooctadiene)nickel (0) (577 mg, 2.10 mmol) in degassed THF (15 mL) was heated under Ar atmosphere at 60 °C until the color became deep violet. Subsequently, a solution of **3** (1.00 g, 1.40 mmol) in degassed THF (10 mL) was added to the reaction mixture. The mixture was stirred at 45 °C for 6 h. The reaction mixture was cooled down to room temperature and THF was evaporated under reduced pressure. The residue was suspended in CH_2Cl_2, filtered through a silica pad, and washed by CH_2Cl_2. The filtrate was washed with brine, dried over Na_2SO_4, filtered, and evaporated under reduced pressure. The crude product was purified by MPLC (EtOAc:hexane = 5:95) to give BC (532 mg, 69% yield) as a yellow solid. Recrystallization from hexane gave yellow needles.

Mp: 236 °C (decomp.). IR (KBr): ν 2958, 2901, 2867, 1460, 1437, 1361, 1289, 1276, 1247, 1138 cm^{-1}. ^1H NMR (CD_2Cl_2 + NEt_3): δ 8.07 (2H, d, J = 1.6 Hz), 7.90 (2H, d, J = 8.8 Hz), 7.78 (2H, d, J = 1.2 Hz), 7.56 (2H, dd, J = 8.8, 1.6 Hz), 7.54 (2H, d, J = 1.2 Hz), 1.456 (18H, s), 1.450 (18H, s) ppm. ^{13}C NMR (CD_2Cl_2 + NEt_3): δ 145.6, 142.8, 137.9, 136.2, 123.3, 121.8, 120.7, 118.4, 117.1, 116.0, 114.5, 110.9, 34.9, 34.6, 31.7, 31.6 ppm. HRMS (m/z) for $C_{40}H_{46}N_2$ (M^+): Calculated 554.3661, found 554.3664.

3,3',6,6'-Tetra-*tert*-butyl-1,1'-bicarbazole (4) from 2

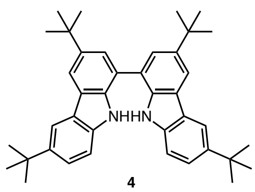

4

A solution of 2,2'-bipyridyl (131 mg, 0.839 mmol) and 1,5-cyclooctadiene (100 µL, 0.815 mmol), and bis(1,5-cyclooctadiene)nickel (0) (231 mg, 0.840mmol) in degassed THF (2 mL) was heated under Ar atmosphere at 60 °C until the color became deep violet. Subsequently, a solution of **2** (100 mg, 0.279 mmol) in degassed THF (2 mL) was added to the reaction mixture. The mixture was stirred at 80 °C for 6 h. The reaction mixture was cooled down to room temperature and THF was evaporated under reduced pressure. The residue was suspended in CH_2Cl_2, filtered through a silica pad, and washed by CH_2Cl_2. The filtrate was washed with brine, dried over Na_2SO_4, filtered, and evaporated under reduced pressure. The crude product was purified by PTLC (EtOAc:hexane = 10:90) to give **4** (68 mg, 87% yield) as a colorless solid.
Mp: 212 °C. IR (KBr): ν 3414, 2954, 2901, 2867, 1756, 1627, 1574, 1490, 1468, 1392, 1357, 1293, 1263, 1198, 1141, 1099 cm^{-1}. ^{1}H NMR ($CDCl_3$): δ 8.17 (2H, d, *J* = 2.0 Hz), 8.14 (2H, d, *J* = 1.2 Hz), 7.79 (2H, s), 7.64 (2H, d, *J* = 2.0 Hz), 7.45 (2H, dd, *J* = 8.8, 2.0 Hz), 7.24 (2H, d, *J* = 8.8 Hz), 1.52 (18H, s), 1.46 (18H, s) ppm. ^{13}C NMR ($CDCl_3$): δ 143.2, 142.7, 138.1, 136.0, 128.8, 124.5, 123.9, 123.6, 121.5, 116.5, 115.9, 110.4, 35.0, 34.8, 32.3, 32.2 ppm. HRMS (*m*/*z*) for $C_{40}H_{48}N_2$ (M^+): Calculated 556.3817, found 556.3820.

3,3',6,6'-Tetra-*tert*-butyl-1,1'-bicarbazole (4) from 1

To a solution of **1** (1.00 g, 3.58 mmol) in CH_2Cl_2 (100 mL) was added dropwise a solution of $FeCl_3$ (1.16 g, 7.16 mmol) in CH_2Cl_2 (50 mL) at room temperature. After stirring for 15 min, the reaction was quenched by addition of MeOH (20 mL). The reaction mixture was added to water (100 mL) and extracted with CH_2Cl_2 (50 mL×3). The organic layer was washed by aqueous Na_2HCO_3 solution (30 mL×1), dried over Na_2SO_4, filtered, and concentrated under reduced pressure. The residue was purified by MPLC (EtOAc:hexane = 12:88) to give **4** (408 mg, 41% yield) as a colorless solid.

3,3',6,6'-Tetra-*tert*-butyl-1,1',9,9'-bicarbazole (BC) from 4

A solution of **4** (50.0 mg, 0.0898 mmol) in pyridine (5 mL) was warmed up to 70 °C. A solution of Bu_4NMnO_4 (64.9 mg, 0.179 mmol) in pyridine (5 mL) was added dropwise to the reaction mixture slowly at 70 °C. The reaction mixture was stirred at 70 °C for 24 h. The reaction mixture was cooled down to room temperature and pyridine was evaporated under reduced pressure. EtOAc (20 mL) and water (30 mL) were added the residue, and the slurry was stirred at room temperature for 30 min. The precipitated brown solid was filtered out by glass-sintered funnel and washed by EtOAc. The filtrate was washed by water (10 mL×2) and brine (10 mL×1), dried over Na_2SO_4, filtered, and concentrated under reduced pressure. The crude product was purified by PTLC (EtOAc:hexane = 2:98) to give BC (32 mg, 65% yield) as a yellow solid.

4-Bromo-2,7-di-*tert*-butyl-9,10-dihydro -9,9-dimethylacridine (6)

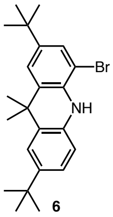

6

To a solution of 2,7-di-*tert*-butyl-9,10-dihydro-9,9-dimethylacridine **5** (1.31 g, 4.06 mmol) in $CHCl_3$ (50 mL) was added *N*-bromosuccinimide (745 mg, 4.18 mmol) at 60 °C. The reaction was stirred for 1 h at 60 °C, and quenched by addition of saturated aqueous $Na_2S_2O_3$ solution. The mixture was cooled down to room temperature, and extracted with CH_2Cl_2 (30 mL×3). The organic layer was washed with water (30 mL×1), dried over Na_2SO_4, filtered, and concentrated under reduced pressure. The residue was purified by MPLC (CH_2Cl_2:hexane = 10:90 to 12:88) to give amorphous solid (1.15 g). This amorphous solid was again purified by GPC to give **6** (922 mg, 57% yield) as white solid.

Mp: 95-98 °C (crystal from hexane). IR (ATR): ν 3416, 2960, 2925, 2899, 2863, 1608, 1571, 1499, 1461, 1407, 1382, 1361, 1319, 1265, 1204, 1152, 1131, 1094, 937, 924, 891, 870, 858, 813, 747, 736, 641 cm^{-1}. ^1H NMR ($CDCl_3$ + NEt_3): δ 7.38 (1H, d, *J* = 2.1 Hz), 7.34 (2H, s), 7.15 (1H, dd, *J* = 8.2, 2.2 Hz), 6.74 (1H, d, *J* = 8.6 Hz), 6.65 (1H, s), 1.58 (6H, s), 1.32 (9H, s), 1.30 (9H, s) ppm. ^{13}CNMR ($CDCl_3$ + NEt_3): δ 143.72, 143.68, 135.6, 133.9, 130.4, 128.3, 126.7, 123.7, 121.9, 121.5, 113.6, 108.2, 37.2, 34.35, 34.31, 31.5, 3 1.4, 30.5 ppm. HRMS (*m*/*z*) for $C_{23}H_{30}{}^{79}BrN$ (M^+): Calculated 399.1562, found 399.1562.

2,2',7,7'-Tetra-*tert*-butyl-9,9',10,10'-tetrahydro-9,9,9',9'-tetramethyl-4,4'-biacridine (7)

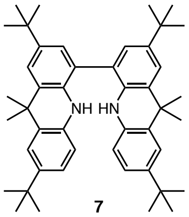

7

To a mixture of 2,2'- bipyridyl (1.08 g, 6.90 mmol), 1,5-cyclooctadiene (0.85 mL, 6.9 mmol), and bis(1,5-cyclooctadiene)nickel (0) (1.90 g, 6.90 mmol) in degassed tetrahydrofuran (30 mL)(deep violet color) was added a solution of **6** (922 mg, 2.30 mmol) in degassed tetrahydrofuran (30 mL), and the mixture was stirred at 80 °C for 6 h. The reaction mixture was cooled down to room temperature and tetrahydrofuran was evaporated under reduced pressure. The residue was suspended in CH_2Cl_2, filtered through a silica gel pad, and washed by CH_2Cl_2. The filtrate was evaporated under reduced pressure. The crude product was recrystallized from CH_2Cl_2/CH_3OH to give **7** (698 mg, 95% yield) as a white solid.

Mp: 209-211 °C (crystal from CH_2Cl_2/CH_3OH). IR (ATR): ν 3411, 2953, 2903, 2864, 1742, 1613, 1597, 1504, 1481, 1461, 1410, 1390, 1385, 1360, 1310, 1297, 1272, 1235, 1201, 1151, 1129, 1090, 1047, 881, 849, 822, 761, 749, 687, 669, 644 cm^{-1}. ^1H NMR ($CDCl_3$ + NEt_3): δ 7.49 (2H, d, J = 2.1 Hz), 7.40 (2H, d, J = 2.1 Hz), 7.13 (2H, d, J = 2.1 Hz), 7.01 (2H, dd, J = 8.2, 2.1 Hz), 6.39 (2H, d, J = 8.2 Hz), 6.14 (2H, s), 1.68 (6H, s), 1.66 (6H, s), 1.35 (18H, s), 1.30 (18H, s) ppm. ^{13}C NMR ($CDCl_3$ + NEt_3): δ 143.1, 142.9, 136.5, 134.6, 129.5, 128.4, 125.5, 123.4, 121.6, 121.5, 113.4, 37.1, 34.4, 34.3, 31.7, 31.6, 29.9, 29.8 ppm. HRMS (*m*/*z*) for $C_{40}H_{60}N_2$ (M^+): Calculated 640.4756, found 640.4747.

2,2',7,7'-Tetra-*tert*-butyl-9,9',10,10'-tetrahydro-9,9,9',9'-tetramethyl-4,4',10,10'-biacridine (TBA)

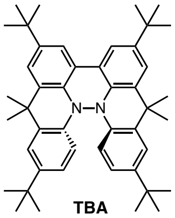

TBA

To a solution of **7** (698 mg, 1.09 mmol) in pyridine (30 mL) was added Bu_4NMnO_4 (987 mg, 2.73 mmol) at ambient temperature, and the reaction mixture was stirred at that temperature for 12 h. The reaction was quenched by addition of saturated aqueous $Na_2S_2O_3$ solution. The precipitated brown solid was filtered out by Celite pad and washed with CH_2Cl_2. The filtrate was extracted with CH_2Cl_2 (30 mL×3). The organic layer was washed with water (30 mL×1), dried over Na_2SO_4, filtered, and concentrated under reduced pressure. The residue dissolved in toluene was filtered through a silica gel pad and washed by toluene:hexane = 10:90. The filtrate was evaporated under reduced pressure, and recrystallized from CH_2Cl_2/CH_3OH to give TBA (598 mg, 86% yield) as a yellow solid.

Mp: 273-276 °C (crystal from CH_2Cl_2/CH_3OH)(decomp.). IR (ATR): ν 2959, 2904, 2866, 1742, 1601, 1496, 1459, 1419, 1379, 1362, 1278, 1265, 1255, 1239, 1227, 1127, 1097, 1081, 927, 895, 882, 871, 863, 849, 835, 811, 766, 747, 703, 686, 669, 651, 593 cm^{-1}. ^1H NMR (CD_2Cl_2 + NEt_3): δ 7.51 (2H, d, J = 2.1 Hz), 7.49 (2H, d, J = 2.1 Hz), 7.32 (2H, d, J = 1.8 Hz), 7.09 (2H, dd, J = 8.6, 2.2 Hz), 6.91 (2H, d, J = 8.2 Hz), 1.97 (6H, s), 1.50 (6H, s), 1.37 (18H, s), 1.29 (18H, s) ppm. ^{13}C NMR (CD_2Cl_2 + NEt_3): δ 145.7, 145.0, 141.0, 136.5, 132.2, 132.0, 123.4, 121.2, 120.7, 118.9, 116.3, 110.9, 37.6, 34.9, 34.7, 31.7, 31.6, 29.4, 24.3 ppm. HRMS (*m*/*z*) for $C_{46}H_{58}N_2$ (M^+): Calculated 638.4600, found 638.4597

Quenching experiment of disproportionation of BC by hydrazine or NEt_3 (Scheme 4)

To a solution of BC (11.1 mg, 0.0200 mmol) in CH_2Cl_2 (20 mL) was added CF_3CO_2H (0.031 mL, 0.40 mmol) at room temperature. The solution was stirred for 1 h until $BC^{\bullet+}CF_3CO_2^-$ and BCH_3^+ $CF_3CO_2^-$ were quantitatively

generated. The solution was quenched by addition of hydrazine monohydrate (0.097 mL, 2.00 mmol, 10000 mol%) at 30 °C. After stirring or 1 h at 30 °C, the mixture was cooled down to room temperature and the solvent was evaporated. The residue was diluted by CH_2Cl_2, washed by water, dried over Na_2SO_4, filtered, and concentrated under reduced pressure. The residue was purified by MPLC (EtOAc:hexane = 3:97) to give BC (7.5 mg, 68% yield) and BCH_2 (3.5 mg, 31% yield). It was also confirmed by UV-Vis measurements that only BC in CH_2Cl_2 was not reduced to BCH_2 by hydrazine. In the similar manner, the disproportionation mixture was quenched by NEt_3 (0.11 mL, 0.80 mmol, 4000 mol%) to afford BC (8.0 mg, 72% yield), BCH_2 (2.0 mg, 18% yield), and **8** (trace, 3% yield by NMR)

8: IR (ATR): ν 2952, 2922, 2853, 2360, 1602, 1488, 1462, 1442, 1381, 1361, 1291, 1269, 1244, 1224, 1200, 1138, 1042, 869, 835, 808, 759, 662, 620 cm^{-1}. ^1H NMR (CD_2Cl_2): δ 8.03 (2H, d, J = 2.0 Hz), 7.80 (2H, d, J = 1.2 Hz), 7.59 (2H, d, J = 1.6 Hz), 7.53 (2H, s), 7.49 (2H, d, J = 1.6 Hz), 7.41 (2H, d, J = 2.0 Hz), 7.35 (2H, dd, J = 8.8, 2.0 Hz), 7.19 (2H, d, J = 8.8 Hz), 7.08 (2H, dd, J = 5.6, 2.0 Hz), 6.20 (2H, dd, J = 8.8, 1.6 Hz), 6.16 (2H, d, J = 8.4 Hz), 1.46 (18H, s), 1.39 (18H, s), 0.90 (18H, s), 0.84 (18H, s) ppm. ^{13}C NMR (CD_2Cl_2): δ 143.2, 142.7, 141.4, 140.2, 137.9, 137.8, 137.1, 134.5, 126.9, 123.4, 123.3, 123.2, 123.1, 121.9, 121,8, 120.8, 120.0, 119.9, 116.7, 116.4, 115.7, 115.4, 109.7, 107.4, 34.7, 34.5, 34.0, 33.9, 31.8, 31.4, 31.3 ppm. LRMS (MALDI-TOF)(*m/z*) for $C_{80}H_{94}N_4$ (M^+): Calculated 1110.75, found 1110.26.

Quenching experiment of disproportionation of TBA by hydrazine (Scheme 5)

To a solution of TBA (51.0 mg, 0.0798 mmol) in CH_2Cl_2 (30 mL) was added CF_3CO_2H (0.12 mL, 1.6 mmol, 2000 mol%) at ambient temperature. The solution was stirred for 30 min until $TBA^{\bullet+}TFA^-$ and $TBH_4^{2+}2TFA^-$ were quantitatively generated. The mixture was warmed up to 30 °C and quenched by addition of hydrazine monohydrate (0.38 mL, 7.8 mmol, 10000 mol%). After stirring for 2 h at that temperature, the mixture was cooled down to room temperature, dried over Na_2SO_4, filtered, washed by ethyl acetate, and concentrated under reduced pressure. In the crude ^1H-NMR spectrum (CD_2Cl_2+NEt_3), the ratio of TBA and $TBAH_2$ was 2:1. The residue was purified by silica gel column chromatography (toluene:hexane=5:95 to 33:67) to give TBA (33.1 mg, 65% yield) and $TBAH_2$ (16.4 mg, 32% yield). It was also confirmed by UV-Vis measurements that only TBA in CH_2Cl_2 under neutral condition without CF_3CO_2H was not reduced to $TBAH_2$ by hydrazine.

3. ^{1}H and ^{13}C NMR Spectra

^{1}H NMR of 3

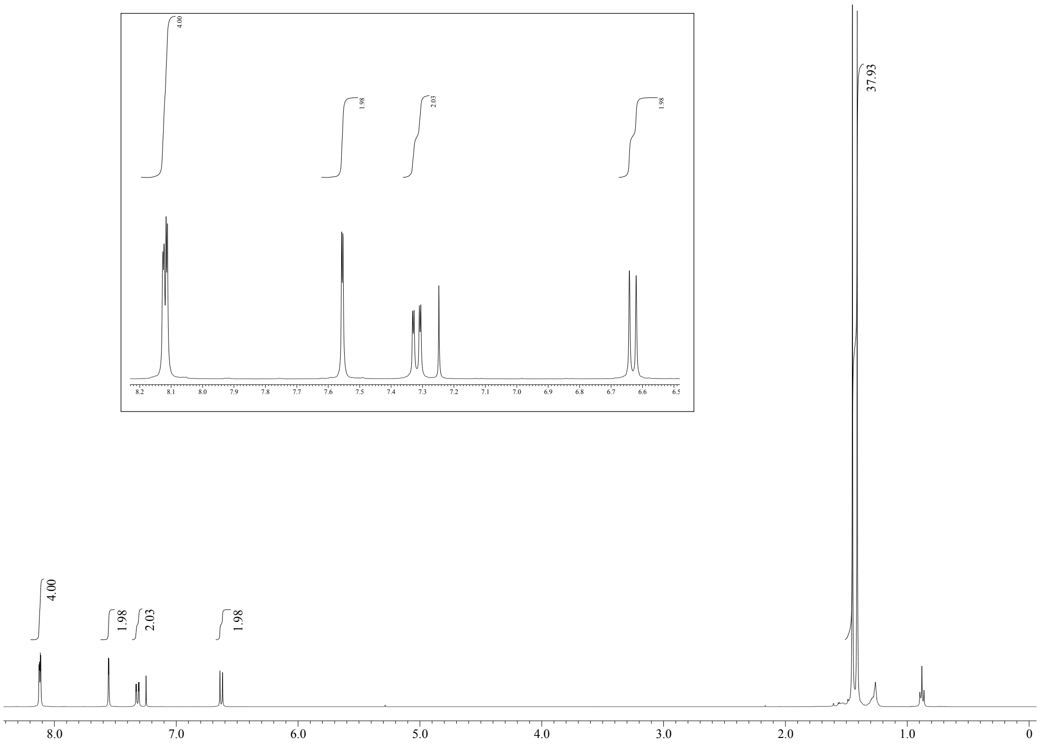

^{13}C NMR of 3

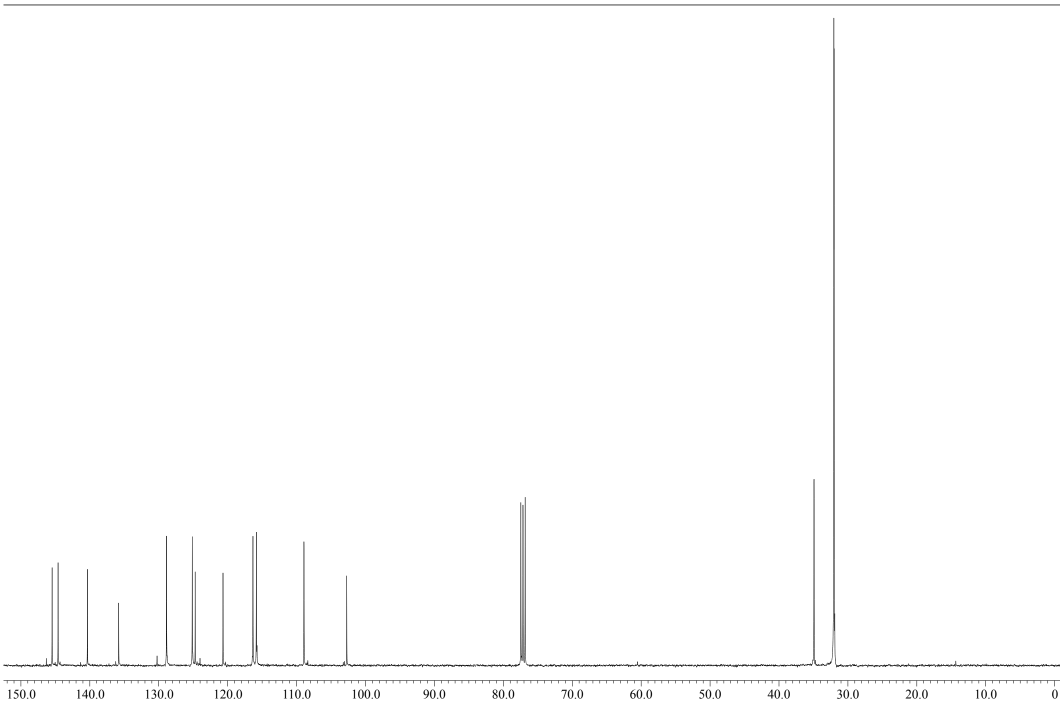

^{1}H NMR of BC in $CDCl_3$

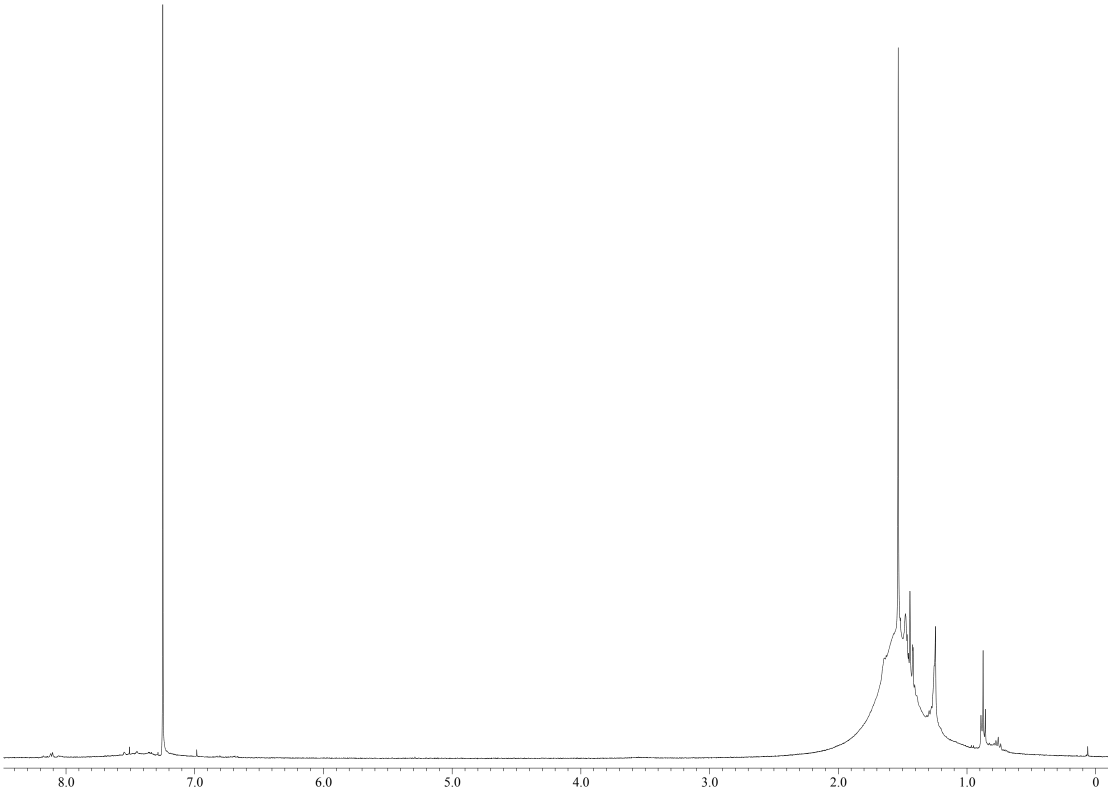

^{1}H NMR of BC in freshly distilled CD_2Cl_2

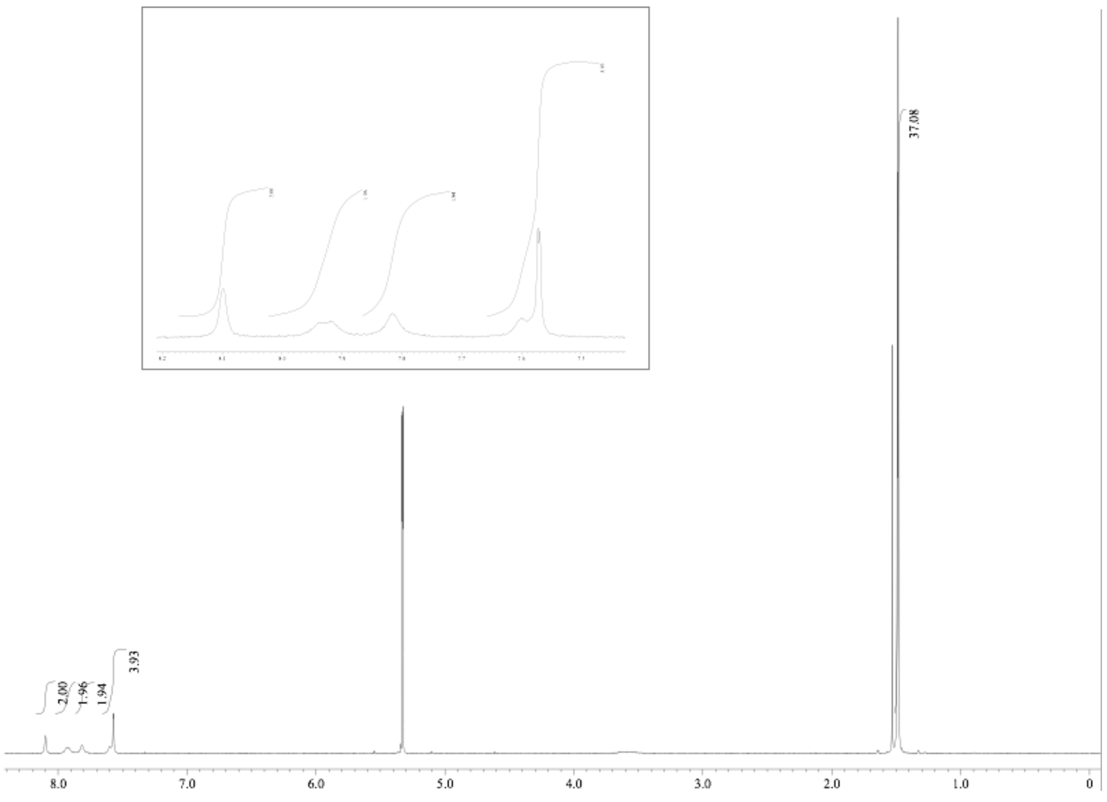

^{1}H NMR of BC with 200 mol% NEt_3 in CD_2Cl_2

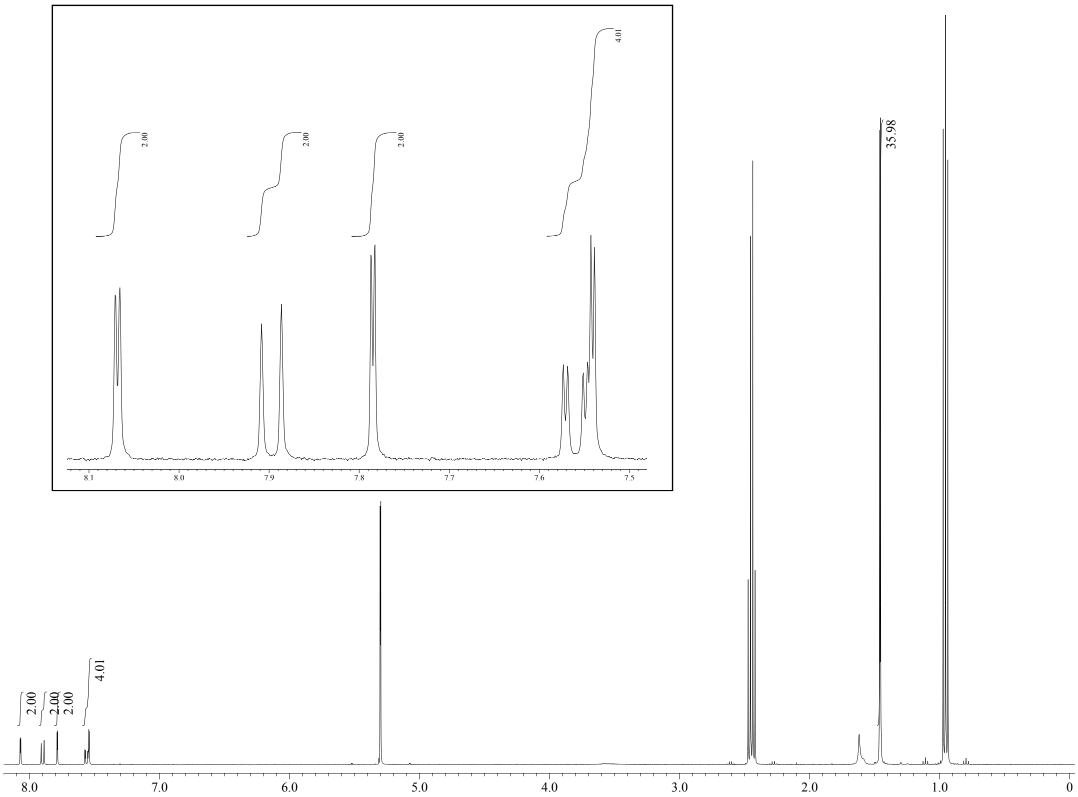

^{1}H NMR of BC with 200 mol% CF_3CO_2H in CD_2Cl_2

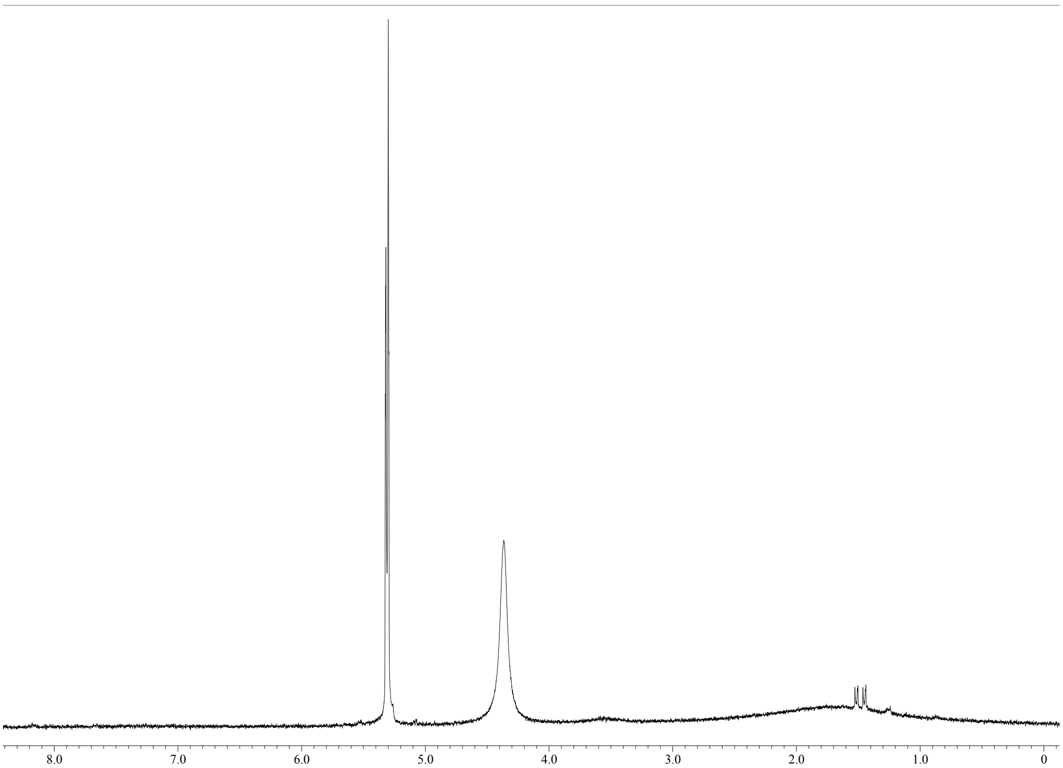

^{13}C NMR of BC with NEt_3 in CD_2Cl_2

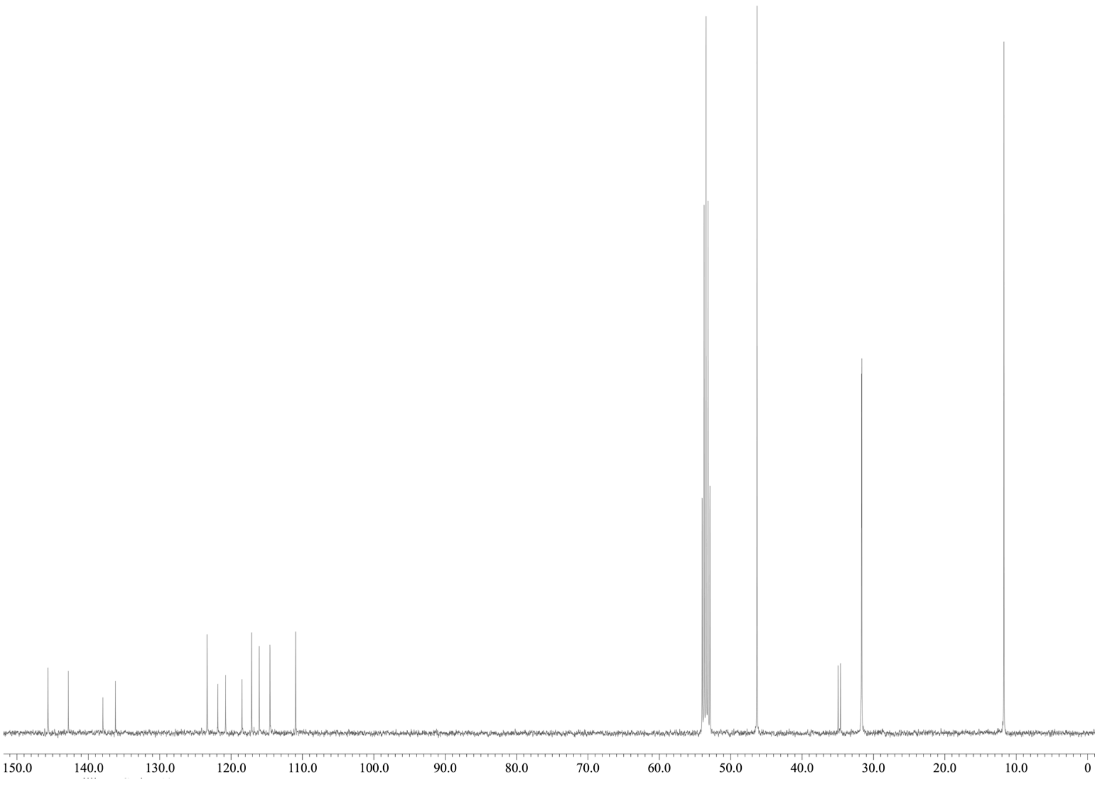

^{1}H NMR of 4

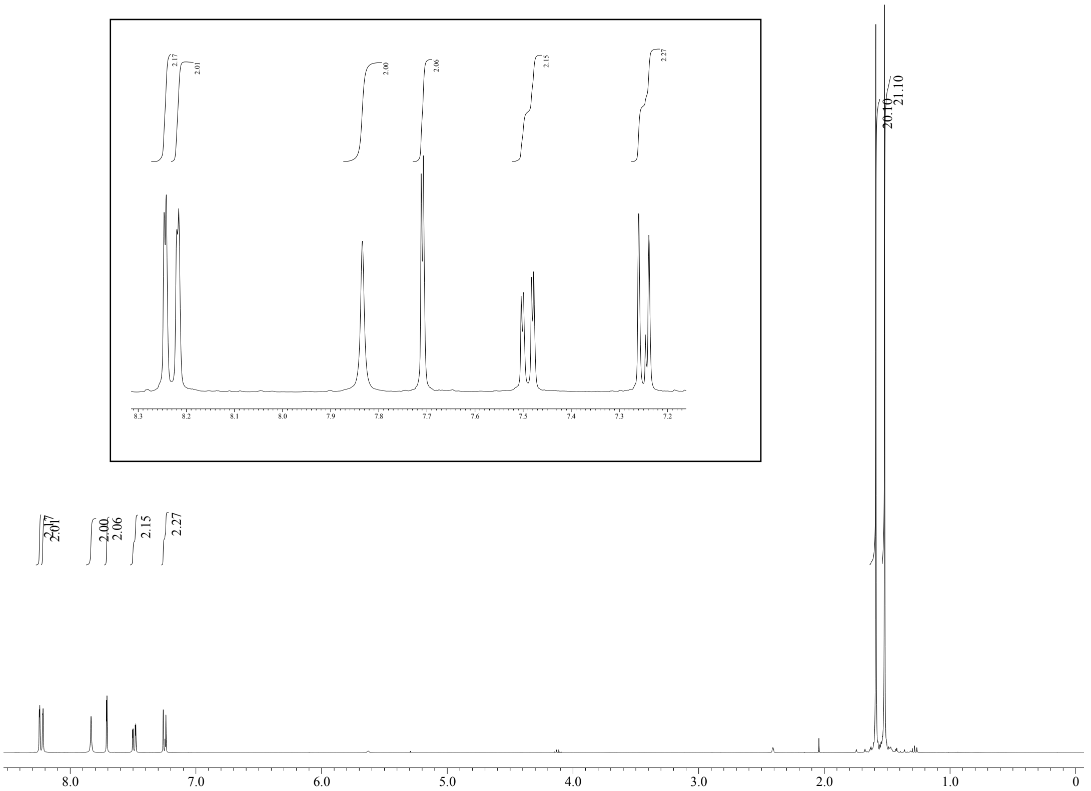

^{13}C NMR of 4

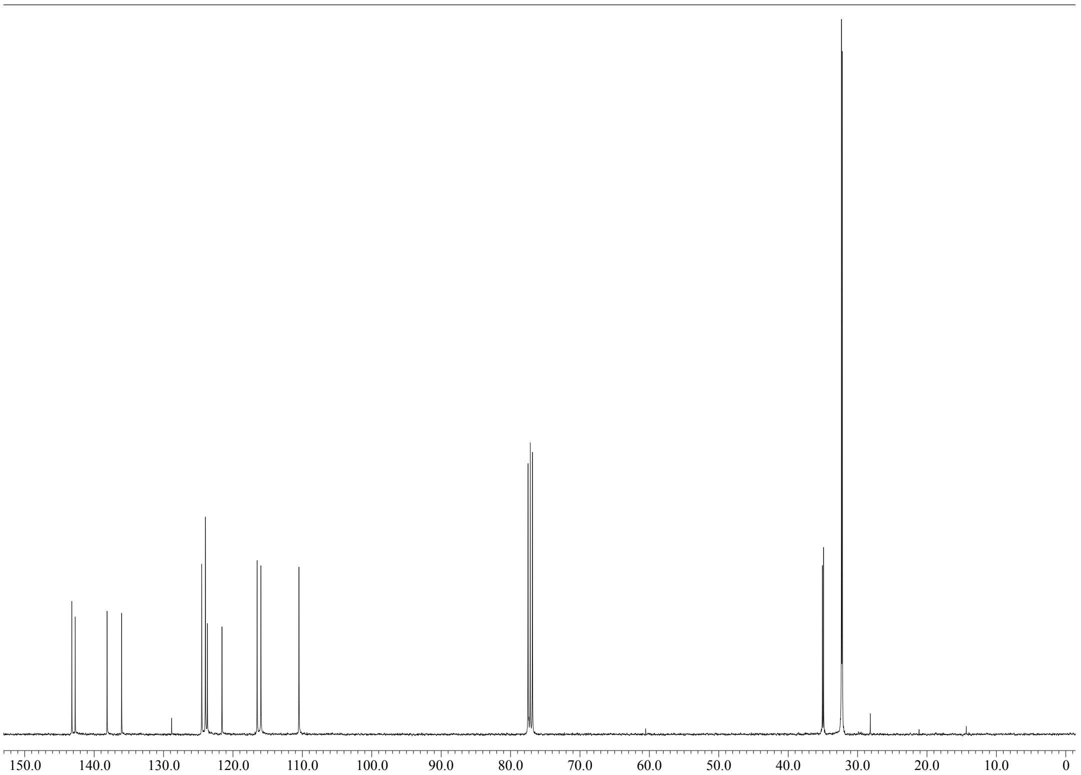

^{1}H NMR of 6

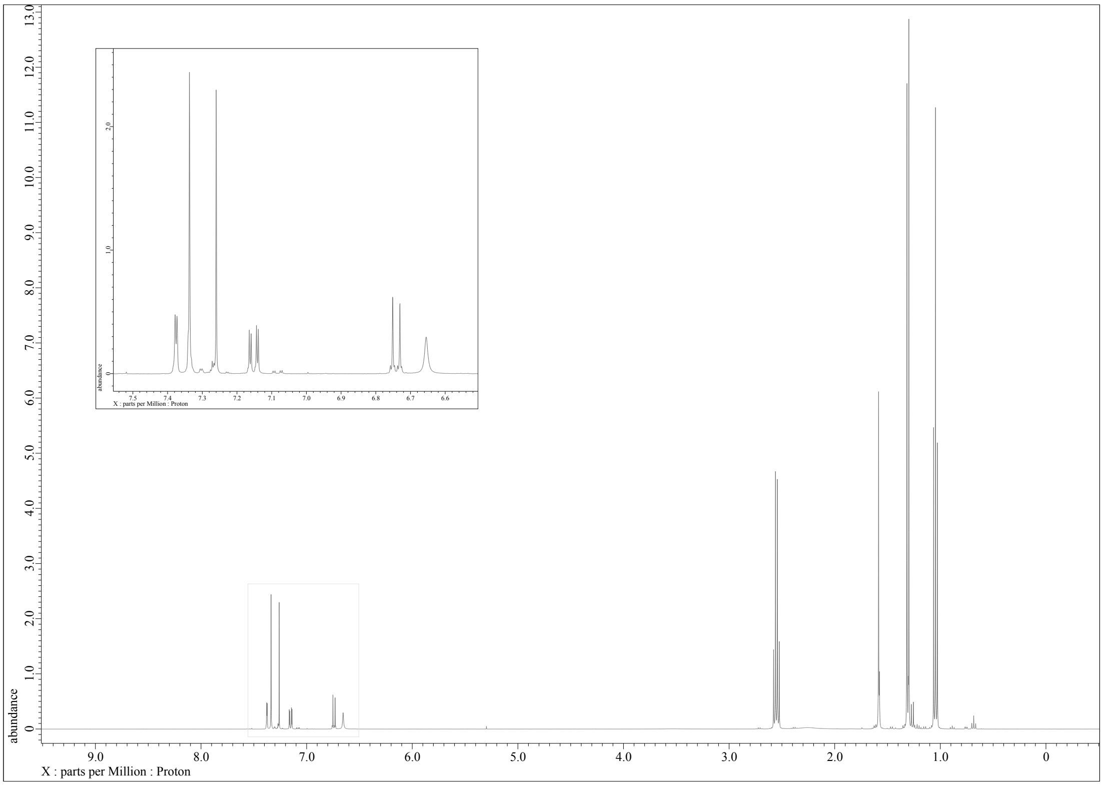

^{13}C NMR of 6

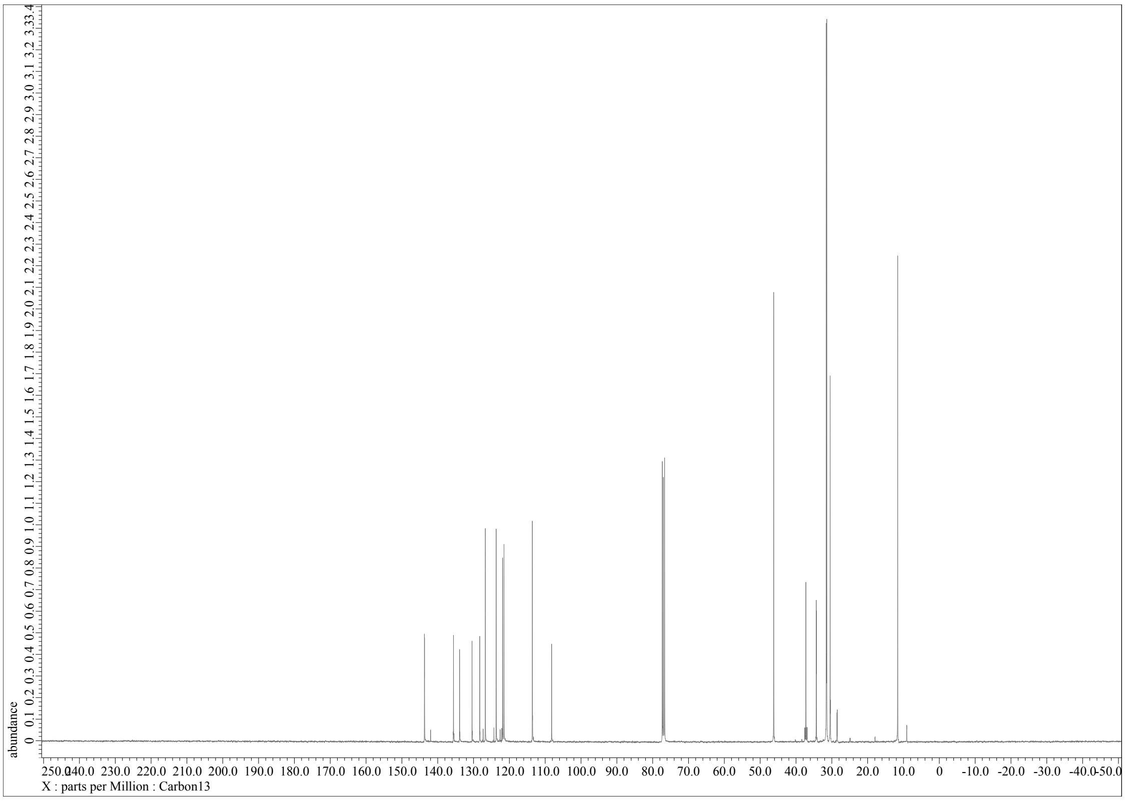

^{1}H NMR of 7

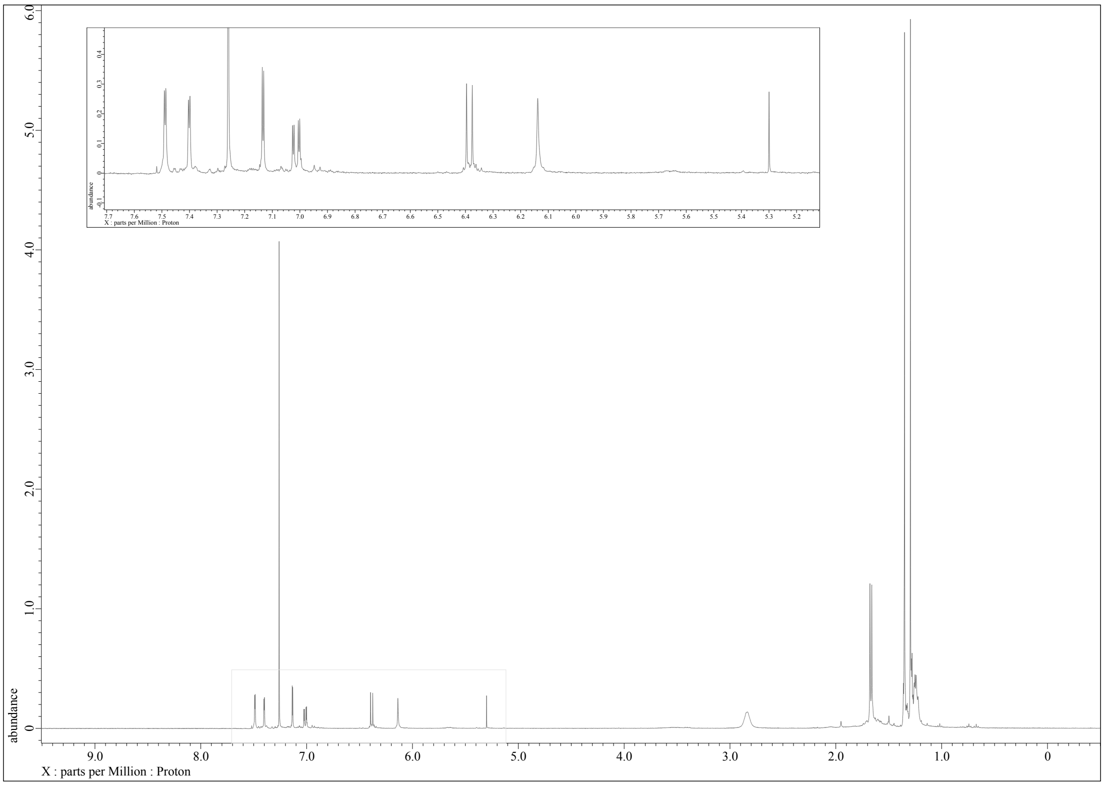

^{13}C NMR of 7

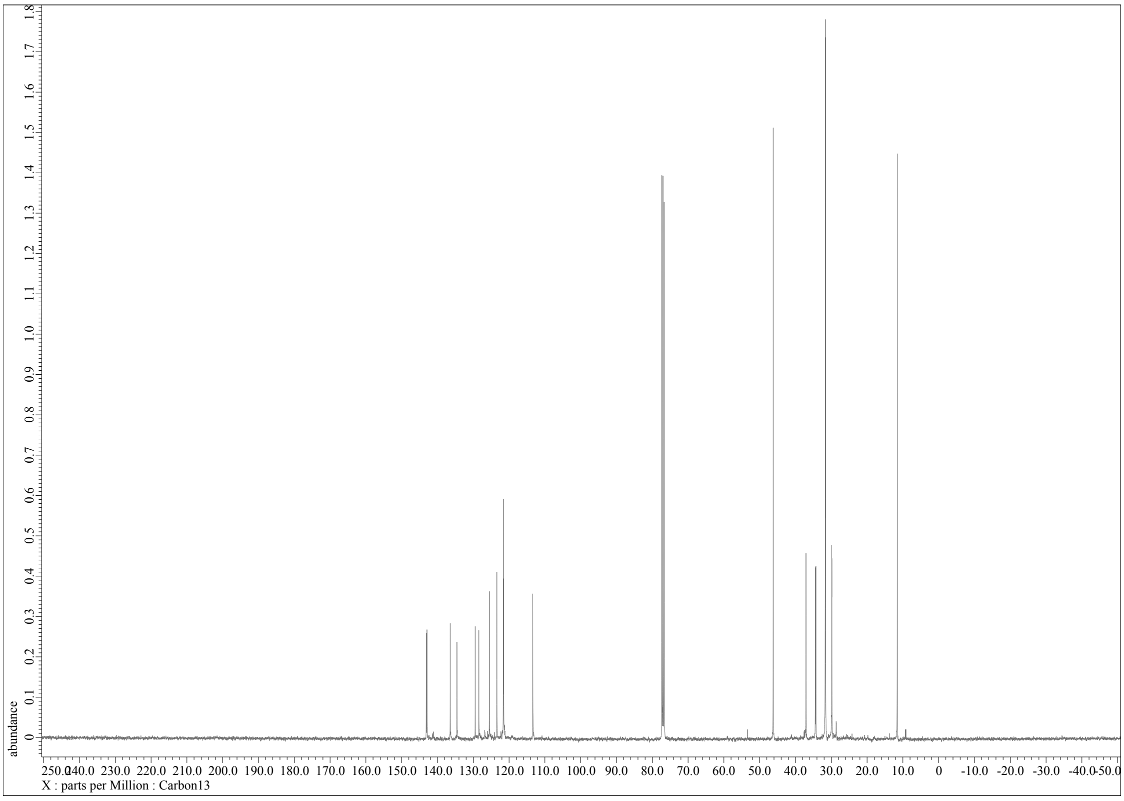

^{1}H NMR of TBA in freshly distilled CD_2Cl_2

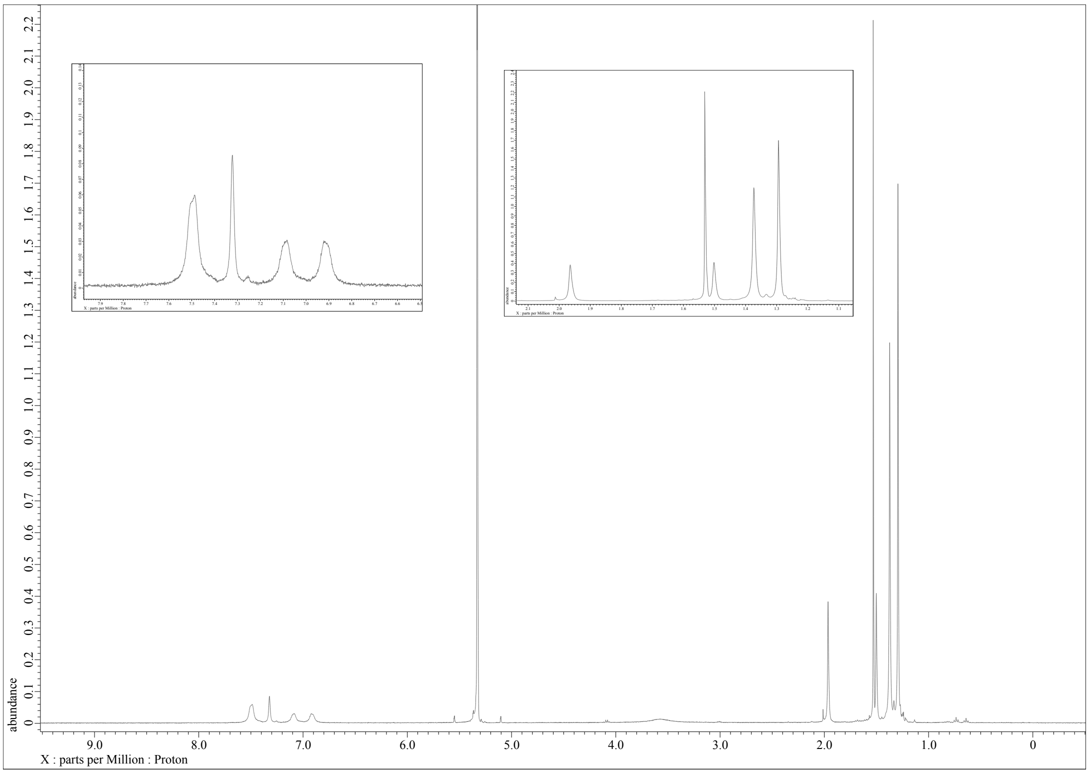

^{1}H NMR of TBA with 1000 mol% NEt_3 in CD_2Cl_2

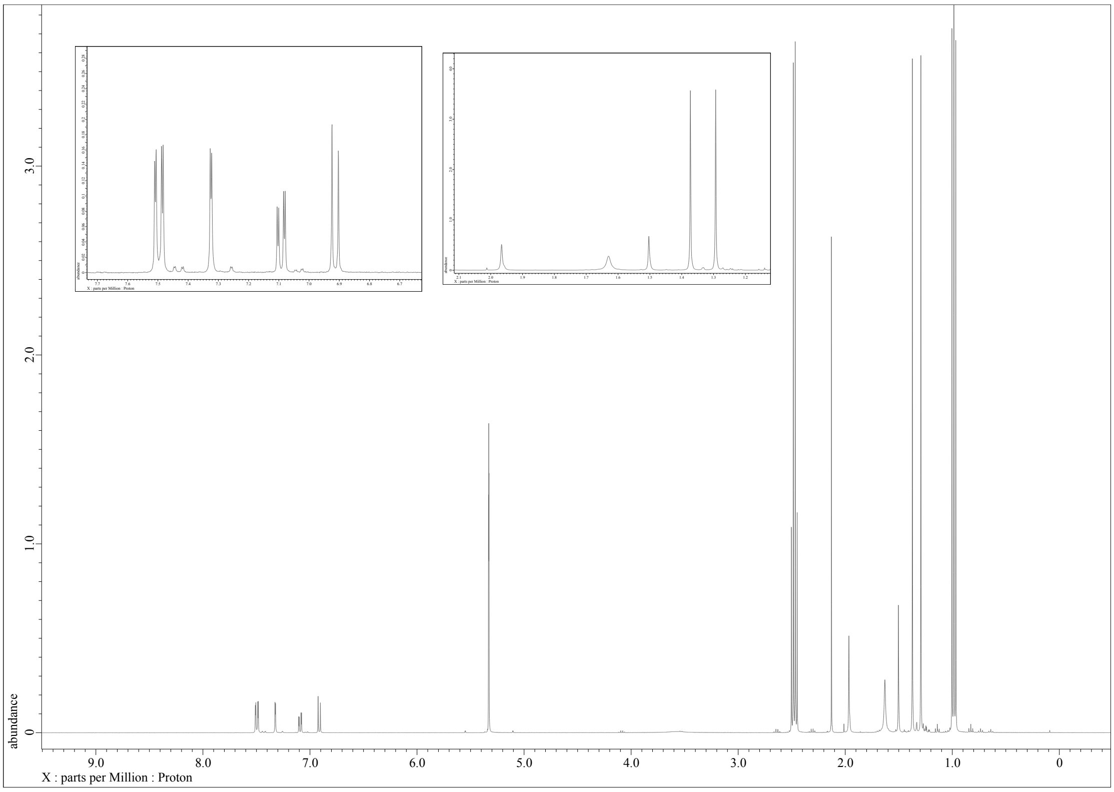

^{1}H NMR of TBA with 1000 mol% NEt_3 + 3000 mol% CF_3CO_2H in CD_2Cl_2

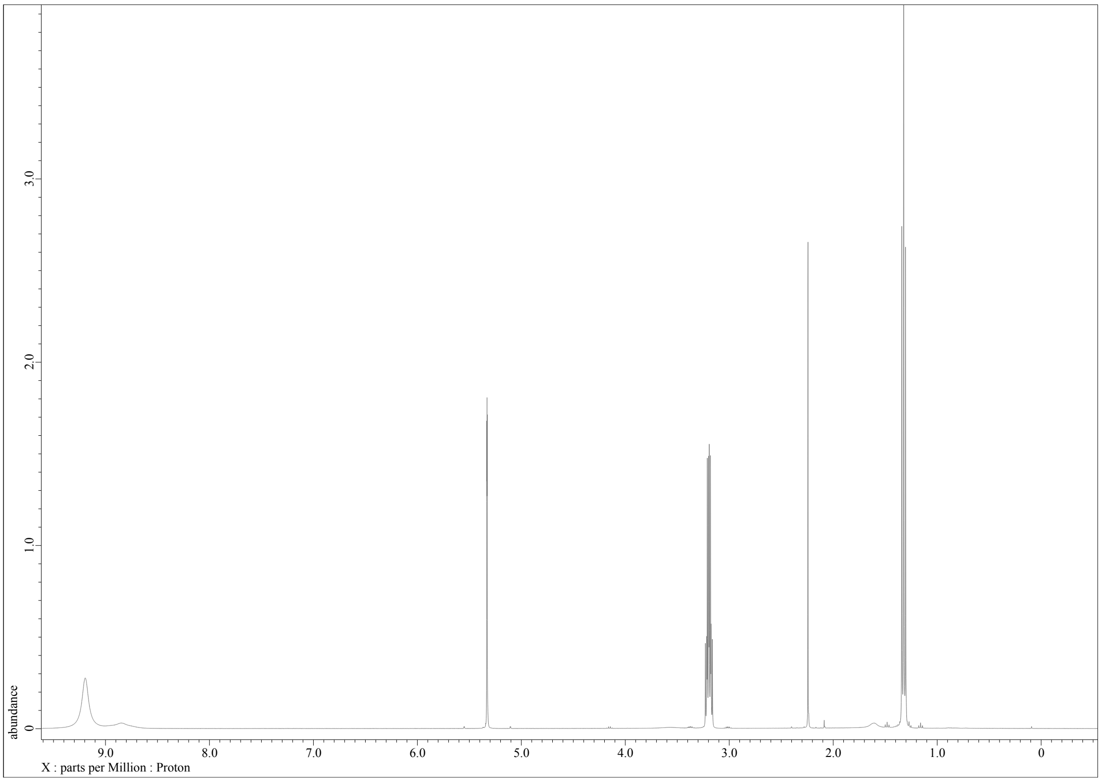

^{1}H NMR of TBA with 1000 mol% NEt_3 + 3000 mol% CF_3CO_2H +4000 mol% NEt_3 in CD_2Cl_2

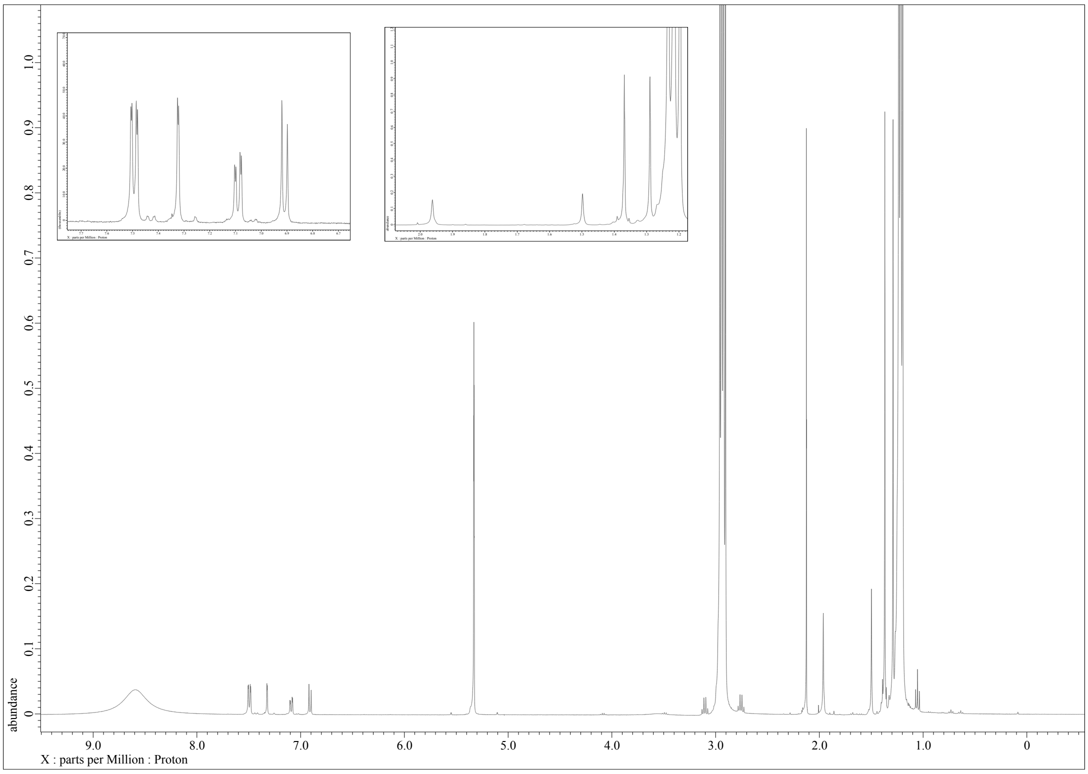

^{13}C NMR of TBA with NEt_3 in CD_2Cl_2

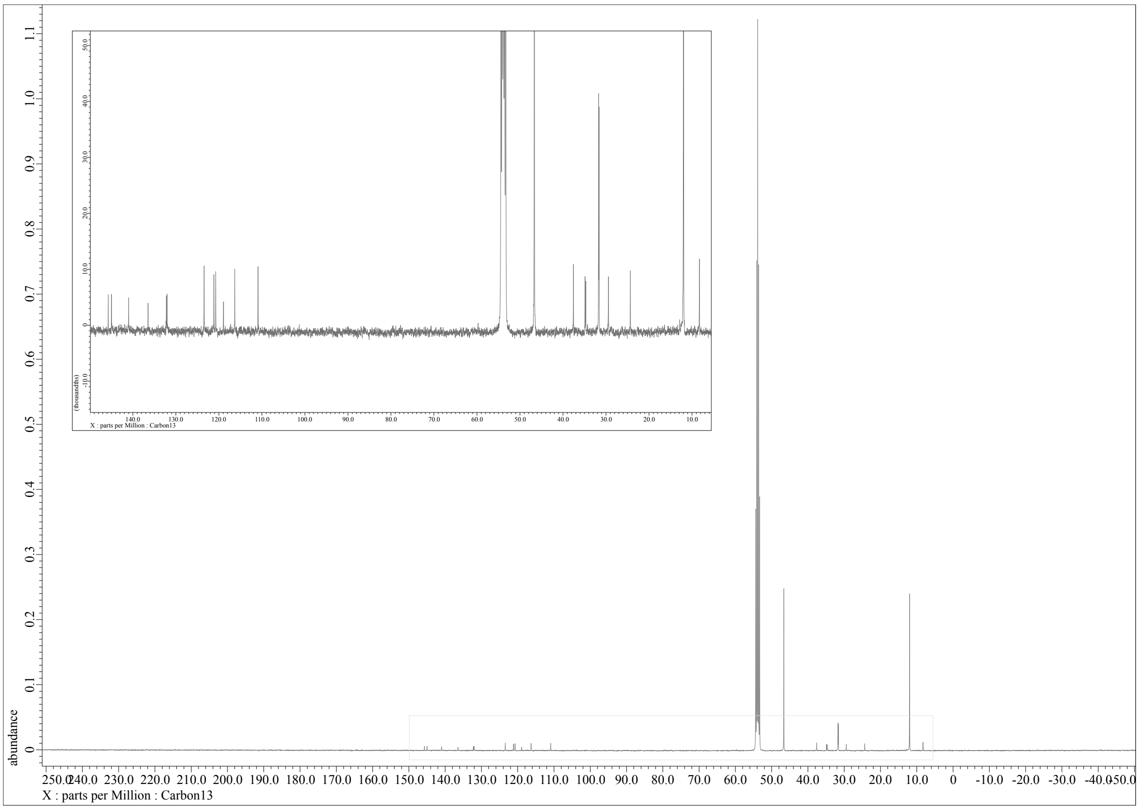

^{1}H NMR of 8

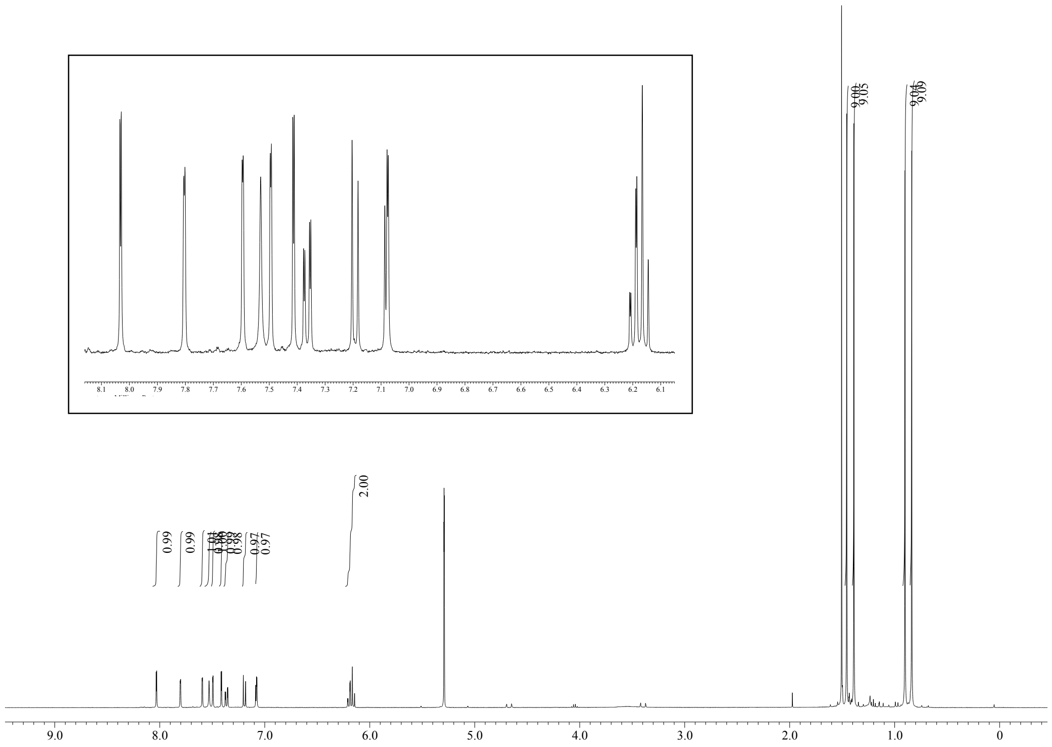

^{13}C NMR NMR of 8

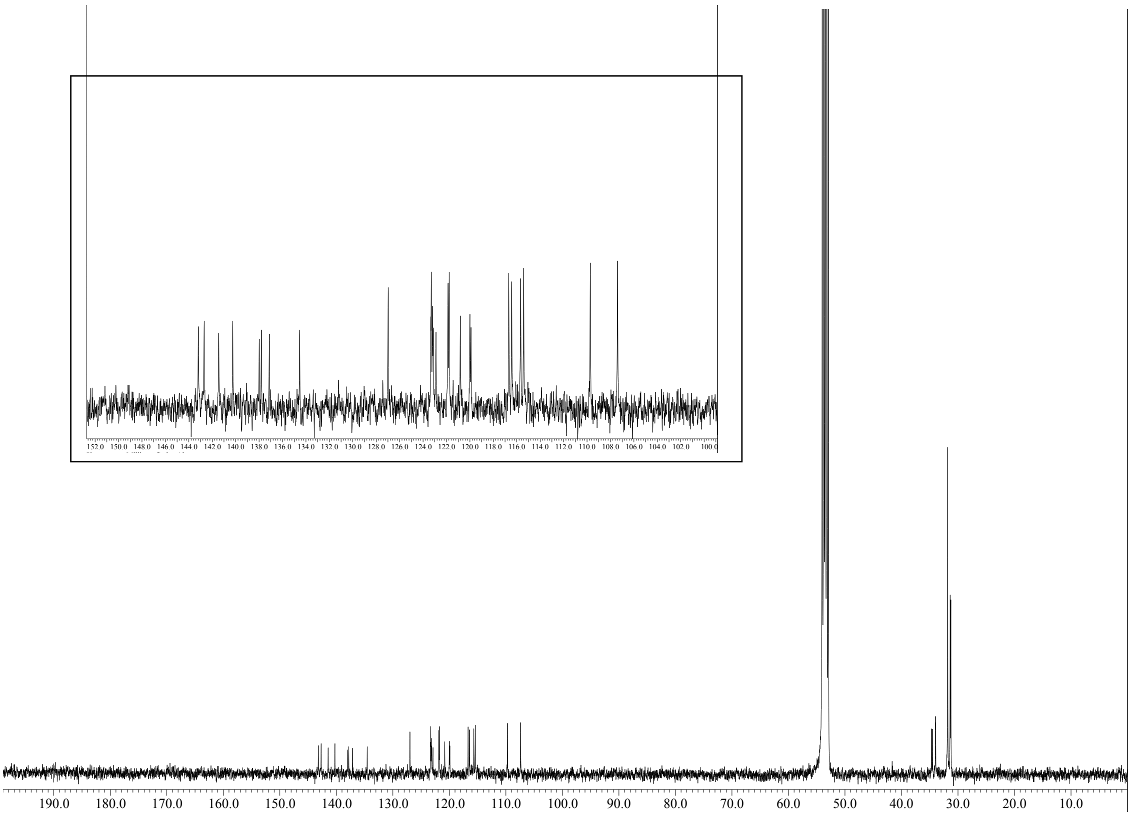

4. Solid State NMR Analysis

In order to confirm the state of solid BC, the solid state NMR spectra of ground crystals of BC were measured. The solid state NMR experiments were carried out using a Bruker Avance 600 spectrometer (proton resonance frequency of 600 MHz) equipped with a 2.5 mm O.D. ^{1}H-^{13}C-^{15}N triple resonance MAS probe. Temperature was controlled to 293 K using VT controller. MAS frequency was controlled to either 20 or 10 kHz. Typical ^{1}H, ^{13}C and ^{15}N pulses were 100, 88 and 66 kHz, respectively. ^{1}H heteronucleardipolar decoupling and FSLG homonuclear dipolar decoupling were carried out at rf fields of 100 kHz. ^{1}H heteronulcear dipolar decouplings were achieved by TPPM[3] and SPINAL64[4] under 20 and 10 kHz spinning speed, respectively. Repetition time was 2 s. ^{1}H and ^{13}C peaks were assigned from the contact time variation of LGCPMAS[5,6], ^{1}H-^{13}C dipolar dephased^{13}C-CPMAS spectra[7], ^{1}H-^{13}C FSLG-HETCOR[8] and ^{1}H-^{13}C MELODI-HETCOR[9] spectra. ^{1}H-^{13}C HETCOR spectra were recorded with a total 128 t_1 increments with 1000 scans each. Quadrature detection in F1 was achieved using States method. ^{1}H isotropic chemical shifts under FSLG were corrected by experimentally obtained scaling factorsof reference sample at same condition. Data processing was performed using Bruker TOPSPIN2.1, and TAKE NMR1.42[10]. Spectral analyses were performed using SPARKY3.114[11]. The summary of ^{1}H- and ^{13}C-isotropic chemical shifts and the spectra are shown in Table S1, Fig. S1-S4.

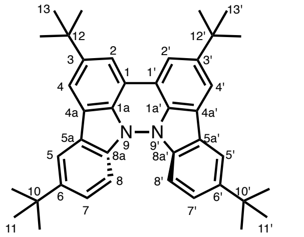

Table S1.

	Isotropic Chemical shifts (ppm) (tentative)			Isotropic Chemical shifts (ppm) (tentative)	
Sites	^{13}C	^{1}H	Sites	^{13}C	^{1}H
1a, 1a’	137.74	-	6,6’	141.39	-
	137.19	-		140.40	-
1,1’	120.92	-	7,7’	118.10	6.05
2,2’	115.17	6.99		116.40	6.24
	113.29	6.84	8,8’	111.60	6.73
3,3’	144.67	-		109.90	6.82
4,4’	115.17	6.99	8a,8a’	135.51	-
	113.29	6.84	10,10’	35.68	-
4a,4a’	120.92	-	11,11’	32.89	1.66
	119.65	-		31.84	0.35
5,5’	122.92	6.58	12,12’	34.02	-
	120.88	6.71	13,13	32.89	1.66
5a,5a’	120.92	-		31.84	0.35
	119.65	-			

5. X-ray Crystallographic Analysis

The single crystal of BC was obtained by the slow vaporization from the hexane solution. The single crystal of **8** was obtained by the slow evaporation from the $CHCl_3$/hexane solution. The analyses were performed on a Rigaku VariMax RAPID system (Mo-Kα, λ = 0.71073 Å, T = 123 K, 2θmax = 55.0°). The single crystal of TBA was obtained from CH_2Cl_2/C_2H_5OH. The analysis was performed on a Rigaku Saturn CCD system (Mo-Kα, λ = 0.71069 Å, T = 123 K, 2θmax = 55.0°). The structures were solved by direct method (SIR-2004 or -2008) and refined using SHELXL-97 or -2013. For the analysis on **8**, contributions from disordered solvent molecules were removed by the SQUEEZE program (PLATON). The single crystal of $BC^{\bullet+}I_5^-\cdot IC_6H_5$ was obtained as follows; To the solution of BC (2.5 mg, 3.9 μmol) in iodobenzene (1.0 ml) in a thin glass pipet tube was added MeOH (0.5 ml) and I_2 (10 mg, 39 μmol) in MeOH (0.5 ml) successively and the solution was kept in freezer. The analysis was performed on a Bruker APEX-II CCD (Mo-Kα, λ = 0.71073 Å, T = 100 K, 2θmax = 48.2°). The structure was solved by direct method (SHELXS-97) and refined using SHELXL-97. Crystallographic data have been deposited with Cambridge Crystallographic Data Centre: Deposition number CCDC-1025063 (BC), CCDC-1038914 ($BC^{\bullet+}I_5^-\cdot IC_6H_5$), CCDC-1049677 (**8**), and CCDC-1040722 (TBA). Copies of the data can be obtained free of charge via http://www.ccdc.cam.ac.uk/conts/retrieving.html (or from the Cambridge Crystallographic Data Centre, 12, Union Road, Cambridge, CB2 1EZ, UK; Fax: +44 1223 336033; e-mail: deposit@ccdc.cam.ac.uk).

Crystal data of BC: $C_{40}H_{46}N_2$, yellow, 0.5×0.2×0.02 mm^3, monoclinic, space group $P2_1/c$ (No. 14), a = 10.7010(2), b = 27.6694(5), c = 11.1215(3) Å, α = 90°, β = 98.764(1)°, γ = 90°, V = 3254.52(12) Å^3, ρ_{calcd} = 1.132 g/cm^3, Z = 4, 5933 reflections measured, R_1 = 0.0413 [$I > 2\sigma(I)$], and wR_2 = 0.1137 (all data), GOF=1.069.

Crystal data of $BC^{\bullet+}I_5^-\cdot IC_6H_5$: $C_{46}H_{51}I_6N_2$, dark red, 0.3×0.15×0.01 mm^3, monoclinic, space group $P2_1/c$ (No. 14), a = 15.047(3), b = 24.399(5), c = 13.754(3) Å, α = 90°, β = 108.90(3)°, γ = 90°, V = 4777.4(17) Å^3, ρ_{calcd} = 1.937 g/cm^3, Z = 4, 8306 reflections measured, R_1 = 0.0350 [$I > 2\sigma(I)$], and wR_2 = 0.0824 (all data), GOF=1.029.

Crystal data of **8**: $C_{80}H_{94}N_4$, orange-brown, 0.50×0.14×0.07 mm^3, monoclinic, space group $C2/c$ (No. 15), a = 30.6695(11), b = 12.1512(5), c = 23.0584(9) Å, α = 90°, β = 119.5715(9)°, γ = 90°, V = 7473.9(5) Å^3, ρ_{calcd} = 0.988 g/cm^3, Z = 4, 7079 reflections measured, R_1 = 0.0756 [$I > 2\sigma(I)$], and wR_2 = 0.2301 (all data), GOF=1.425.

Crystal data of TBA: $C_{46}H_{58}N_2$, yellow, 0.20×0.10×0.02 mm^3, monoclinic, space group $P2_1/c$ (No. 14), a = 13.312(3), b = 18.814(4), c = 15.401(4) Å, α= 90°, β= 97.762(5)°, γ= 90°, V = 3821.8(1) Å^3, ρ_{calcd} = 1.11 g/cm^3, Z = 4, 8664 reflections measured, R_1 = 0.0893 [$I > 2\sigma(I)$], and wR_2 = 0.2496 (all data), GOF=1.061.

6. ESR Spectral Measurements

Temperature dependent measurements (Fig. 8a, S6b, c)

A sample solution of BC, TBA, or 1-oxyl-2,2,6,6-tetramethyl-4-hydroxypiperidine (TEMPOL)(1.00 mM) in in a ESR tube was degassed by the freeze-pump-thaw cycles three times, and the ESR tube was sealed. ESR spectrum at different temperature was recorded in a same setting of parameters. The *IT* values (*I*=double integral of ESR signal, *T*=temperature) of BC and TBA were plotted versus temperature. They were slightly decreased by increasing the temperature (Fig. 8a, S6b). To confirm that this slight decrease comes from the machinery error, the temperature dependence of *IT* value of ESR signal of TEMPOL (*S*=1/2) was measured, showing the similar decrease of *IT* values by increasing the temperature (Fig. S6c). Thus, we concluded that the *IT* values are constant and the spin state is doublet.

7. UV-Vis-NIR Spectral Experiments

UV-Vis-NIR measurements were conducted with 1.0 cm quartz cell at 20 °C under air, unless otherwise noted.

7.1. UV-Vis-NIR spectral experiment to show the reaction of BC and acid under equilibrium (Fig. S7a)

UV-Vis-NIR spectrum of BC (1.00 mM) with 2000 mol% CF_3CO_2H in CH_2Cl_2 with 1 mm cell was measured (Fig. S7a, orange). The solvent and excess CF_3CO_2H of the solution was evaporated in vacuo. The residue was dissolved in CH_2Cl_2 (1.00 mM) and the spectrum was measured again after 5 h. The comparison of these two spectra indicated the shift of equilibrium reaction under acidic condition depending on the amount of acid.

7.2. UV-Vis-NIR spectra of $BC^{•+}$ and $TBA^{•+}$ by electrochemical oxidation of BC and TBA (Fig. 9a, 16h)

UV-Vis-NIR spectral measurements under the electrochemical oxidation of BC or TBA were carried out on a solution of BC or TBA (1.00 mM) in 1,2-dichloroethane containing 0.1 M Bu_4NClO_4 using a BAS 1 mm spectroelectrochemical quartz cell equipped with a Pt mesh working electrode, a Pt wire counter electrode, and a Ag/AgCl reference electrode (Fc/Fc^+ $E_{1/2}$ = 0.25 V). In each measurement, the indicated voltage in Fig. 9a and 16h was applied for 120 s, and the spectral measurement started at 90 s after application of voltage. The indicated voltages in Fig. 7a are given in vs. Ag/AgCl.

7.3. UV-Vis-NIR spectra of $BC^{•+}$ and $TBA^{•+}$by chemical oxidation

7.3.1. UV-Vis-NIR spectra of $BC^{•+}$ by chemical oxidation of BC with $NOPF_6$ (Fig. 9b)

To a solution of BC (4.00 mM) in CH_2Cl_2 (1 mL) in a glove box under N_2 atmosphere was added 50, 100, 150, 200, 250, 300, 400, 500, 1000, or 2000 mol% of $NOPF_6$ using the 300 mM solution in acetonitrile at ambient temperature, respectively. The solution was diluted by CH_2Cl_2 to 0.05 mM based on added BC and left over for 1 day at ambient temperature under N_2 atmosphere in a glove box before the spectral measurement. The sample solution was transferred into a 1 cm quartz cell in a grove box and the measurement was conducted under N_2 atmosphere. The absorption intensity at 635 nm associated with $BC^{•+}$ was 0.76 with 150 to 300 mol% $NOPF_6$

and was decreased according to the increase of oxidant more than 300 mol%. This result indicated that $BC^{•+}$ was quantitatively generated with 150~300 mol% $NOPF_6$ and $BC^{•+}$ was further oxidized to BC^{2+} with more than 300 mol% $NOPF_6$. The spectrum with 150 mol% $NOPF_6$ is shown in Fig. 9b. The molar absorption coefficient ε_{635}=1.5×10^4 L mol^{-1} cm^{-1} of $BC^{•+}$ at 635 nm was obtained from the spectrum with 150 mol% $NOPF_6$. The value was used for the kinetic experiments.

7.3.2. UV-Vis-NIR spectra of $BC^{•+}$ by chemical oxidation of BC with I_2 (Fig. S7a)

To a solution of BC (4.00 mM) in dichloroethane (2 mL) was added 100, 200, 500, 1000, 2000, 3000 mol% of a I_2 solution (20 mM) in dichloroethane at ambient temperature, respectively. The solution was diluted by dichloroethane to 0.100 mM based on added BC and left over for 1 day under dark at ambient temperature before the spectral measurement. The absorption associated with $BC^{•+}$ was increased according to the increase of oxidant up to 3000 mol% I_2. The spectrum with 3000 mol% I_2 are shown in Fig. S7a.

7.3.3. UV-Vis-NIR spectra of $TBA^{•+}$ by chemical oxidation of TBA with I_2 or DDQ (Fig. 16i)

To a solution of TBA (1.00 mM) in 1,2-dichloroethane (0.30 mL) was added 100, 200, 500, 1000, or 2000 mol% of a I_2 or DDQ solution (30 mM) in 1,2-dichloroethane at ambient temperature, respectively. The solution was diluted by 1,2-dichloroethane to be 3.0 mL with 0.100 mM based on added TBA and left over for 1 day at ambient temperature before the spectral measurement. The absorption associated with $TBA^{•+}$ was increased according to the increase of oxidant and saturated at 1000 mol% I_2 or 500 mol% DDQ. The absorption of $TBA^{•+}$ with 1000 mol% I_2 or 500 mol% DDQ was coincident with that with 2000 mol% I_2 or 1000 and 2000 mol% DDQ. The absorption associated with TBA^{2+} was not admitted in the spectra with both oxidants. These results demonstrate that $TBA^{•+}$ was quantitatively generated with 1000 mol% I_2 or 500 mol% DDQ, and $TBA^{•+}$ was not further oxidized to TBA^{2+} with more amount of these oxidants. The absorbance at 821 nm with 1000 mol% I_2 or 500 mol% DDQ was 0.78 in both cases, which is regarded as 100% $TBA^{•+}$, since both I_2 and DDQ do not have the absorption at 821 nm. The value was used for the determination of the yield of $TBA^{•+}$ generated under acidification of TBA. The spectra with 1000 mol% I_2 and 500 mol% DDQ are shown in Fig. 16i.

7.3.4. UV-Vis-NIR spectra of $TBA^{•+}$ by chemical oxidation of TBA with $NOPF_6$ (Fig. 16i)

To a solution of TBA (1.00 mM) in CH_2Cl_2 (0.30 mL) under N_2 atmosphere in a glove box was added 100, 200, 500, 1000, or 2000 mol% of $NOPF_6$ using the 300 mM solution in acetonitrile at ambient temperature, respectively. The solution was diluted by CH_2Cl_2 to be 3.0 mL with 0.100 mM based on added TBA and left over for 1 day at ambient temperature under N_2 atmosphere in a glove box before the spectral measurement. The sample solution was transferred into a 1 cm quartz cell in a grove box and the measurement was conducted under

N_2 atmosphere. The absorption intensity at 824 nm associated with $TBA^{•+}$ was 0.79 with 100 mol% $NOPF_6$ and was decreased according to the increase of oxidant more than 200 mol%. The absorption associated with TBA^{2+} was clearly admitted with 2000 mol% $NOPF_6$. This result indicated that $TBA^{•+}$ was quantitatively generated with 100~200 mol% $NOPF_6$ and $TBA^{•+}$ was further oxidized to TBA^{2+} with more than 200 mol% $NOPF_6$. The spectrum with 100 mol% $NOPF_6$ is shown in Fig. 16i. The molar absorption coefficient ε_{822}=7.9×10^3 L mol^{-1} cm^{-1} of $TBA^{•+}$ at 822 nm was obtained from the spectrum with 100 mol% $NOPF_6$. The value was used for the kinetic experiments.

7.4. Determination of the composition ratio of BC or TBA and CF_3CO_2H by Job's continuous variation plot

7.4.1. Determination of the ratio of BC and CF_3CO_2H by Job's continuous variation plot (Fig. 13)

The solutions of the mixture of BC and CF_3CO_2H were prepared in the ratio of 1:0, 4:1, 2:1, 3:2, 1:1, 2:3, 1:2, 1:4, 0:1 in CH_2Cl_2 with the constant concentration of BC+CF_3CO_2H = 2.00 mM. After storing at room temperature under dark for 10 days to reach the equilibrium, the UV-Vis-NIR spectra were measured with 1 mm cell. The absorbance at 635 nm of $BC^{•+}$was used for the continuous plot because BC and CF_3CO_2H do not have absorbance at 635 nm.

7.4.2. Determination of the ratio of TBA and CF_3CO_2H by Job's continuous variation plot (Fig. 17, S8h)

The solutions of TBA and CF_3CO_2H in the molar ratio of 1:0, 2:1, 1:1, 1:1.1, 1:1.2, 1:1.3, 3:4, 1:1.4, 1:1.5, 1:1.6, 1:1.7, 1:1.8, 1:1.9, 1:2, 1:3, 1:4, and 0:1 in CH_2Cl_2 were prepared by mixing TBA solution (4.00 mM) and CF_3CO_2H solution (4.00 mM) with the constant total concentration (TBA+CF_3CO_2H=4.00 mM). After storing at room temperature for 3 h to reach the equilibrium, the UV-Vis-NIR spectra were measured with a 1 mm quartz cell. The absorbance at 824 nm associated with $TBA^{•+}$ was used for the continuous plot, because TBA and CF_3CO_2H do not have the absorbance at 824 nm. Selected spectra are shown in Fig. 17 for clarity.

7.4.3. UV-Vis-NIR spectrum of TBA with CF_3CO_2H under N_2 atmosphere. (Fig. S8a)

CH_2Cl_2 with CaH_2 was degassed by the freeze-pump-thaw cycles three times and distilled by the valve-to-valve method. NEt_3 and CF_3CO_2H were degassed by the freeze-pump-thaw cycles three times. Preparation of a solution of TBA (0.100 mM) in CH_2Cl_2 with 2000 mol% of CF_3CO_2H was performed in a grove box with 2-5 ppm O_2 level during experiment. The sample solution was transferred into a 1 cm quartz cell in a grove box and the measurement was conducted under N_2 atmosphere.

7.4.4. UV-Vis-NIR spectral study on the reversibility of the reaction of TBA by acid/base treatment (Fig. 18a, Table S2)

Sample solution of TBA (0.100 mM, CH_2Cl_2, 3.0 mL) was transferred into a 1 cm quartz cell equipped with magnetic stirrer bar and the spectrum under neutral condition was measured. CF_3CO_2H (0.5 μL, 6 μmol, 2000 mol%) was then added to the neutral TBA solution at ambient temperature. After stirring for 30 min at that temperature, the spectrum under acidic condition was measured to confirm the conversion. Then, the acidic solution was neutralized by addition of NEt_3 (1.3 μL, 9 μmol, 3000 mol%) at ambient temperature, and the spectrum was measured after 10 min. Same experiments were conducted 5 times. The recovery yield of TBA was calculated from the absorption intensities at 412 nm (Table S2), and the average was determined to be 98.6% yield with the five experiments.

Table S2. Recovery yield of TBA by acidification/neutralization.

	Absorption at 412 nm in neutral condition	Absorption at 412 nm after neutralization	Recovery yield (%)
Run 1	0.394	0.394	100
Run 2	0.387	0.382	98.7
Run 3	0.384	0.375	97.7
Run 4	0.388	0.381	98.2
Run 5	0.390	0.384	98.5

7.4.5. UV-Vis-NIR spectral study on the reversibility of the reaction of TBA by repeating the addition of acid/base treatment (Fig. 18b, c, d)

Sample solution of TBA (0.100 mM, CH_2Cl_2, 3.0 mL) with 1000 mol% NEt_3 was transferred into a 1 cm quartz cell equipped with magnetic stirrer bar and the spectrum was measured. In each cycle, the spectra were measured at 10-60 min after addition of CF_3CO_2H or NEt_3 at ambient temperature, respectively. The added amounts of CF_3CO_2H and NEt_3 in each cycle are shown as follows;

1st cycle: CF_3CO_2H (0.7 μL, 9 μmol, 3000 mol%), NEt_3 (1.7 μL, 12 μmol, 4000 mol%)

2nd cycle: CF_3CO_2H (1.6 μL, 21 μmol, 7000 mol%), NEt_3 (3.0 μL, 21 μmol, 7000 mol%),

3rd cycle: CF_3CO_2H (2.8 μL, 36 μmol, 12000 mol%), NEt_3 (5.1 μL, 36 μmol, 12000 mol%),

4th cycle: CF_3CO_2H (5.6 μL, 72 μmol, 24000 mol%), NEt_3 (10.2 μL, 72 μmol, 24000 mol%),

5th cycle: CF_3CO_2H (11.2 μL, 144 μmol, 48000 mol%), NEt_3 (30.6 μL, 216 μmol, 72000 mol%)

The spectra were shown in Fig. 18b and the absorbance at 412 and 822 nm were plotted in Fig. 18c, d.

8. Fluorescence Spectral Experiments

8.1. Determination of quantum yield of BC

Quantum yield of the solution of BC (4.50×10^{-5} M) in CH_2Cl_2 was determined to be 0.6875 by the absolute

method using a JASCO FP6500 spectrometer equipped with integrating sphere JASCO ILF-533 using 1 mm cell. For reference, quantum yield of quinine sulfate (5.00×10^{-3} M) in 1 M aq. H_2SO_4 was determined by the same method to be 0.5644, which was comparable to the reported value 0.546.[12]

8.2. Determination of quantum yield of TBA

The quantum yield of TBA was determined by the compared method [equation (1)][13].

$$\Phi_{unk} = (A_{std}/A_{unk}) \text{ x } (F_{unk}/F_{std}) \text{ x } (I_{std}/I_{unk}) \text{ x } (n_{unk}^2/n_{std}^2) \text{ x } \Phi_{std} \quad (1)$$

where Φ, A, F, I, and n are the quantum yield, the absorption at the selected excitation wavelength, the integrated fluorescence signal in the emission region, the relative intensity of the exciting light, and the refractive index of solvent, respectively. Subscripts unk and std stand for unknown and standard sample, respectively. In this measurement, the fluorescence spectra of both unknown and standard samples were measured in benzene with the same excitation wavelength 366 nm. In this condition, the equation (1) is simplified to be equation (2).

$$\Phi_{unk} = (A_{std}/A_{unk}) \text{ x } (F_{unk}/F_{std}) \text{ x } \Phi_{std} \quad (2)$$

9,10-Diphenylanthracene (DPA)(Φ = 0.72, λ_{ex} =366 nm, benzene, 25 °C, under air)[14] was used as standard. Prior to use, DPA was recrystallized twice from CH_2Cl_2/ethanol. The absorption spectra of the sample solutions of TBA (0.100 mM) and DPA (0.100 mM) in benzene were measured under air with 1 cm quartz cell at 20 °C, giving the absorptions A_{TBA} =0.12 and A_{DPA}=0.60 at 366 nm, respectively (Table S3). For the fluorescence spectral measurements, these solutions were diluted by 1% to be 1.00 μM solutions in order to prevent reabsorption of fluorescence. The fluorescence spectra were measured under air with 1 cm quartz cell at 20 °C, giving F_{TBA}=8.2 × 10 and F_{DPA}=1.7 × 10^3, respectively (Table S3). From these values and equation (2), the quantum yield of TBA is determined to be 17% yield.

Table S3. Experimental parameters for determination of the quantum yield of TBA.

A_{TBA}	0.12 (366 nm, 0.100 mM, 1 cm quartz cell, 20 °C, under air)
A_{DPA}	0.60 (366 nm, 0.100 mM, 1 cm quartz cell, 20 °C, under air)
F_{TBA}	8.2 × 10 (λ_{ex} =366 nm, 1.00 μM, 1 cm quartz cell, 20 °C, under air)
F_{DPA}	1.7 × 10^3 (λ_{ex} =366 nm, 1.00 μM, 1 cm quartz cell, 20 °C, under air)

9. Kinetic Experiments

9.1. Determination of the reaction orders of the disproportionation reactions of BC and TBA by the initial rate method

The reaction orders of SM (BC or TBA) and CF_3CO_2H were determined by the initial rate method using the equations (1) and (2), where v_0, k, $[SM]_0$, n, $[TFA]_0$, and m are the initial rate of reaction, the rate constant, the initial concentration of SM, the reaction order of SM, the initial concentration of CF_3CO_2H, and the reaction

order of CF_3CO_2H, respectively.

$$v_0 = k[SM]_0^n[TFA]_0^m \quad (1)$$

It gives equation (2) to take the log of both sides of equation (1).

$$\ln v_0 = \ln k + n \times \ln[SM]_0 + m \times \ln[TFA]_0 \quad (2)$$

9.2. Determination of reaction orders of the disproportionation of BC

9.2.1. Reaction order in BC (Fig. 19a, b, S10a, b, Table 4)

Sample solution (2.00 mL) of CF_3CO_2H (5.00 mM) in CH_2Cl_2 or benzene in a 1 cm quartz cell was set up on a Peltier type constant-temperature unit (UNISOKU, CoolSpeK UV USP-203-B) at 20 °C under Ar atmosphere with magnetic stirring. BC (20.0 mM, 10.0, 20.0, or 30.0 μL) in CH_2Cl_2 or benzene was injected by syringe under magnetic stirring and the absorption of $BC^{•+}$ at 635 nm was monitored at 20 °C (Fig. 19a, S10a). Using the molar absorption coefficient ε_{635} = 1.5×10^4 (CH_2Cl_2) or 1.4×10^4 (benzene) L mol^{-1} cm^{-1} of $BC^{•+}$ and the absorption change (cm^{-1} s^{-1}) between 500~1500 s after well-mixing of the solution, the generation rate (mol L^{-1} s^{-1}) of $BC^{•+}$ at each concentration was determined (Table S4). In the condition using constant excess amount of CF_3CO_2H (5.00 mM), $\ln k + \ln[TFA]_0$ is regarded to be constant in the equation (2). Thus, the reaction order n of BC was determined from the slope of the plot of $\ln v_0$ against $\ln[BC]_0$ to be 0.60 (CH_2Cl_2) or 0.55 (benzene)(Fig. 19b, S10b), which means 1/2 order in BC.

Table S4. Determination of reaction order in BC and the activation barrier energy $\Delta G^‡$ of the disproportionation reaction of BC

$[BC]_0$ (mM)	v_0×10^{-9} (mol L^{-1} s^{-1}) in CH_2Cl_2^{a}	k×10^{-8} in CH_2Cl_2^{b}	$\Delta G^‡$ (kcal mol^{-1}) (293 K) in CH_2Cl_2	v_0×10^{-9} (mol L^{-1} s^{-1}) in benzene^a
0.10	2.3	1.8	27.5	0.84
0.20	3.4	1.9	27.5	1.3
0.30	4.4	2.0	27.5	1.6

[a]500~1500 s. [b]0~3500 s.

9.2.2. Reaction order in CF_3CO_2H (Fig. 19c, d, S10c, d, Table 5)

Sample solution (2.00 mL) of BC (5.00 mM) in CH_2Cl_2 or benzene in a 1 cm quartz cell was set up on a Peltier type constant-temperature unit (UNISOKU, CoolSpeK UV USP-203-B) at 20 °C under Ar atmosphere with magnetic stirring. CF_3CO_2H solution (20.0 mM, 10.0, 20.0, or 30.0 μL) in CH_2Cl_2 or benzene was injected by syringe under magnetic stirring and the absorption of $BC^{•+}$ at 635 nm was monitored at 20 °C (Fig. 19c, S10c). Using the molar absorption coefficient ε_{635} = 1.5×10^4 (CH_2Cl_2) or 1.4×10^4 (benzene) L mol^{-1} cm^{-1} of $BC^{•+}$ and the absorption change (cm^{-1} s^{-1}) between 500~3500 s after well-mixing of the solution, the generation rate (mol L^{-1} s^{-1}) of $BC^{•+}$ at each concentration was determined (Table S5). In the condition using constant excess

amount of CF_3CO_2H (5.00 mM), ln$k$ + ln$[BC]_0$ is regarded to be constant in the equation (2). Thus, the reaction order m of TFA was determined from the slope of the plot of lnv_0 against ln$[TFA]_0$ to be 0.44 (CH_2Cl_2) or 0.44 (benzene)(Fig. 19d, S10d), which means 1/2 order in TFA.

Table S5. Determination of reaction order in CF_3CO_2H and the activation barrier energy $\Delta G^{\ddagger}$ of the disproportionation reaction of BC

$[TFA]_0$ (mM)	$v_0 \times 10^{-9}$ (mol L^{-1} s^{-1}) in CH_2Cl_2[a]	$k \times 10^{-9}$ in CH_2Cl_2[b]	$\Delta G^{\ddagger}$ (kcal mol^{-1}) (293 K) in CH_2Cl_2	$v_0 \times 10^{-10}$ (mol L^{-1} s^{-1}) in benzene[a]
0.10	0.71	4.9	28.3	2.2
0.20	0.87	4.4	28.3	2.8
0.30	1.2	4.6	28.3	3.4

[a]500~3500 s. [b]0~3500 s.

9.3. Determination of the kinetic constant and activation barrier energy of disproportionation of BC

From the experiments on determination of reaction order, the reaction rate equation is expressed to be equation (3), where v, x, k, [BC], [TFA], a, and b are the reaction rate of $BC^{\bullet+}$, the concentration of $BC^{\bullet+}$, the rate constant, the concentration of BC, the concentration of CF_3CO_2H, the initial concentration of BC, and the initial concentration of CF_3CO_2H, respectively.

$$v = dx/dt = k[BC]^{1/2}[TFA]^{1/2} = k[a\text{-}3/2x]^{1/2}[b\text{-}3/2x]^{1/2} \quad (3)$$

In the condition using excess amount of BC or CF_3CO_2H, a-3/2x or b-3/2x is regarded to be the initial concentrations a or b. Thus, the equation (3) becomes (4) or (5).

$$v = dx/dt = k[a]^{1/2}[b\text{-}3/2x]^{1/2} \quad (4)$$

$$v = dx/dt = k[a\text{-}3/2x]^{1/2}[b]^{1/2} \quad (5)$$

The definite integral of the equations give the equation (6) and (7).

$$-4/3(a\text{-}3/2x)^{1/2}b^{-1/2} = kt+C \quad (6)$$

$$-4/3a^{-1/2}(b\text{-}3/2x)^{1/2} = kt+C \quad (7)$$

By fitting the data of reactions between BC and CF_3CO_2H (a = 0.10~0.30 mM and b = 5.00 mM or a = 5.00 mM and b = 0.10~0.30 mM) in CH_2Cl_2 above to the equations (6) or (7), the rate constants k were obtained (Table S4, 5). With the rate constants k and Eyring equation $\Delta G^{\ddagger} = RT \times \ln(k_B T/kh)$, $\Delta G^{\ddagger}$ (293 K) in CH_2Cl_2 was determined to be 28 kcal/mol in average (Table S4, 5).

9.4. Determination of reaction orders of the disproportionation of TBA

9.4.1. Reaction order in TBA (Fig. 20a, b, Table S6)

Sample solution (2.00 mL) of TBA in CH_2Cl_2 with the concentration 0.050, 0.060, 0.080, or 0.10 mM in a 1 cm quartz cell was set up on a Peltier type constant-temperature unit (UNISOKU, CoolSpeK UV USP-203-B)

precooled at -70 °C under Ar atmosphere, and cooled to -70 °C with magnetic stirring. CF_3CO_2H solution (400 mM, 10.0 μL) in CH_2Cl_2 was injected by syringe under magnetic stirring and the absorption of $TBA^{\bullet+}$ at 822 nm was monitored for more than 600 s at -70 °C. Using the molar absorption coefficient ε_{822}=7.9×10^3 L mol^{-1} cm^{-1} of $TBA^{\bullet+}$ and the absorption change (cm^{-1} s^{-1}) between 290~510 s after well-mixing of the solution, the generation rate (mol L^{-1} s^{-1}) of $TBA^{\bullet+}$ at each concentration was determined (Table S6). In the condition using constant excess amount of CF_3CO_2H, ln$k$ + ln[TFA]$_0$ is regarded to be constant in the equation (2). Thus, the reaction order n of TBA was determined from the slope of the plot of lnv_0 against ln[TBA]$_0$ to be 1.2, which means 1st order to TBA.

Table S6. Concentration of TBA and initial rate v_0 (mol L^{-1} s^{-1}) of $TBA^{\bullet+}$

[TBA]$_0$ (mM)	v_0×10^{-9} (mol L^{-1} s^{-1})
0.10	13
0.080	11
0.060	7.6
0.050	6.3

9.4.2. Reaction order in CF_3CO_2H (Fig. 20c ,d, Table S7)

Sample solution (2.00 mL) of TBA (5.00 mM) in CH_2Cl_2 in a 1 cm quartz cell was set up on a Peltier type constant-temperature unit (UNISOKU, CoolSpeK UV USP-203-B) precooled at -60 °C under Ar atmosphere, and cooled to -60 °C with magnetic stirring. CF_3CO_2H solution (25 mM, 5.0, 8.0, 10.0, 12.0, or 14.0 μL) in CH_2Cl_2 was injected by syringe under magnetic stirring and the absorption of $TBA^{\bullet+}$ at 822 nm was monitored for more than 600 s at -60 °C. Using the molar absorption coefficient ε_{822}=7.9×10^3 L mol^{-1} cm^{-1} of $TBA^{\bullet+}$ and the absorption change (cm^{-1} s^{-1}) between 290~510 s after well-mixing of the solution, the generation rate (mol L^{-1} s^{-1}) of $TBA^{\bullet+}$ at each concentration was determined (Table S7). In the condition using constant excess amount of CF_3CO_2H, ln$k$ + ln[TBA]$_0$ is regarded to be constant in the equation (2). Thus, the reaction order m of TFA was determined from the slope of the plot of lnv_0 against ln[TFA]$_0$ to be 2.0, which means 2nd order to TFA.

Table S7. Concentration of TFA and initial rate v_0 (mol L^{-1} s^{-1}) of $TBA^{\bullet+}$

[TFA]$_0$ (mM)	v_0×10^{-9} (mol L^{-1} s^{-1})
0.18	21
0.15	16
0.13	9.8
0.10	10
0.063	2.4

9.5. Determination of the kinetic constant and activation barrier energy of disproportionation of TBA (Fig. S11, Table S8)

From the experiments on determination of reaction order, the reaction rate equation is expressed to be equation (8), where v, x, *k*, [TBA], and [TFA] are the reaction rate of $TBA^{•+}$, the concentration of $TBA^{•+}$, the rate constant, the concentration of TBA, and CF_3CO_2H, respectively.

$$v = dx/dt = k[TBA][TFA]^2 \quad (8)$$

Eq. (8) is rewritten as equation (9).

$$v = dx/dt = k([TBA]_0 - 3/2x)([TFA]_0 -2x)^2 \quad (9)$$

The definite integral of equation (9) gives equation (10);

$$kt + C = \{(2(-3(b - 2x)\ln(3x - 2a) + 4a + 3(b - 2x)\ln(2x - b) - 3b))/((4a - 3b)^2(b -2x))\} \quad (10)$$

where a, b, and C are the initial concentrations of TBA and CF_3CO_2H, and intercept, respectively.

Sample solution (2.00 mL) of TBA (0.200 mM) in CH_2Cl_2 in a 1 cm quartz cell under Ar atmosphere was set up on a Peltier type constant-temperature unit (UNISOKU, CoolSpeK UV USP-203-B) precooled at the target temperature (-45, -50, -53, -56, or -59 °C) and cooled with magnetic stirring. CF_3CO_2H solution (80 mM, 20.0 μL) in CH_2Cl_2 was injected by syringe under magnetic stirring and the absorption of $TBA^{•+}$ at 822 nm was monitored at each temperature for more than 600 sec. The concentration of $TBA^{•+}$ was calculated from the observed absorbance and the molar absorption coefficient ε_{822}=7.9×10^3 L mol^{-1} cm^{-1}. Rate constant *k* at each temperature was determined by the fitting line using the equation (10) and the data between 200 and 500 sec (Table S8).

The Eyring plot in equation (11) gave the enthalpy of activation $\Delta H^‡$ and the entropy of activation $\Delta S^‡$ (Table S8).

$$\ln k/T=-\Delta H^‡/R \cdot 1/T+\ln k_B/h +\Delta S^‡/R \quad (11)$$

From $\Delta H^‡$ and $\Delta S^‡$, the Gibbs free energy of activation $\Delta G^‡$ (293K) was obtained (Table S8). The same experiments were conducted in three times to check reproducibility (Table S8) and the average values were calculated to be $\Delta H^‡$=8.9±0.4 kcal/mol, $\Delta S^‡$=-7.1±1.6 cal/mol·K, and $\Delta G^‡$=11.0±0.1 kcal/mol (293 K). Data from Run 1 are shown in Fig. S11 and the average $\Delta H^‡$ and $\Delta S^‡$ of three runs are shown in Fig. S11c.

Table S8. Summary of rate constant and activation energies

	temp (K)	k (mol^{-2} L^{2} s^{-1})	$\Delta H^{\ddagger}$ (kcal/mol)	$\Delta S^{\ddagger}$ (cal/mol•K)	$\Delta G^{\ddagger}$ (kcal/mol)(293 K)
Run 1	214	110	9.2	-5.9	10.9
	217	130			
	220	156			
	223	264			
	228	391			
Run 2	214	95.2	8.4	-9.4	11.2
	217	126			
	220	165			
	223	215			
Run 3	214	78.0	9.2	-6.0	11.0
	217	129			
	220	158			
	223	195			

10. Theoretical Calculation

All the theoretical calculations were performed at UωB97XD/6-31G(d) level of theory by using the Gaussian 09 program package.[15] The structures of BC, TS-BC, $BC^{\bullet\bullet}$, BCH^{+}, TS-BCH^{+}, $BCH^{\bullet\bullet+}$, BCH_3^{+}, BCH_2, $BC^{\bullet+}$, TBA, TS-TBA, $TBA^{\bullet\bullet}$, $TBAH^{+}$, TS-$TBAH^{+}$, $TBAH^{\bullet\bullet+}$, $TBAH_2^{2+}$, TS-$TBAH_2^{2+}$, $TBAH_2^{\bullet\bullet2+}$, $TBAH_4^{2+}$, $TBAH_2$, $TBA^{\bullet+}$ were optimized and the vibrational frequency analyses were conducted on the optimized structures. The given energies are zero-point corrected. For $BC^{\bullet\bullet}$, $BCH^{\bullet\bullet+}$, BCH_3^{+}, BCH_2, $TBA^{\bullet\bullet}$, $TBAH^{\bullet\bullet+}$, $TBAH_2^{\bullet\bullet2+}$, $TBAH_4^{2+}$, $TBAH_2$, both extended (dihedral angle of two carbazoles or acridines θ>90°) and folded (θ<90°) conformations were calculated and the conformers with lower energies are illustrated in Scheme 6, 7, Fig. 21, 22. For $BC^{\bullet\bullet}$, $BCH^{\bullet\bullet+}$, $TBA^{\bullet\bullet}$, $TBAH^{\bullet\bullet+}$, and $TBAH_2^{\bullet\bullet2+}$, the closed-shell singlet, the triplet, and the open-shell singlet states were calculated, giving the lowest energies for the open-shell singlet states. The energies in Fig. 21a, b and Fig. 22a, b, c are given versus BC, BCH_3^{+}, TBA, $TBAH^{+}$, $TBAH_2^{2+}$, respectively. Calculated spin density distribution of $BC^{\bullet+}$, $TBA^{\bullet+}$ and the open-shell singlet biradical states of $BCH^{\bullet\bullet+}$, $BC^{\bullet\bullet}$, $TBAH_2^{\bullet\bullet2+}$, $TBA^{\bullet\bullet}$ are shown in Fig. 11 and S12.

11. Scheme and Figures

Scheme S1 Reported acid-responsive electron transfer disproportionation. (a) The reaction of tetrathiafulvalene (TTF) with HBF_4. (b) The reaction of 2,2,6,6-tetramethyl-1-piperidinyloxyl (TEMPO) with conc. H_2SO_4.

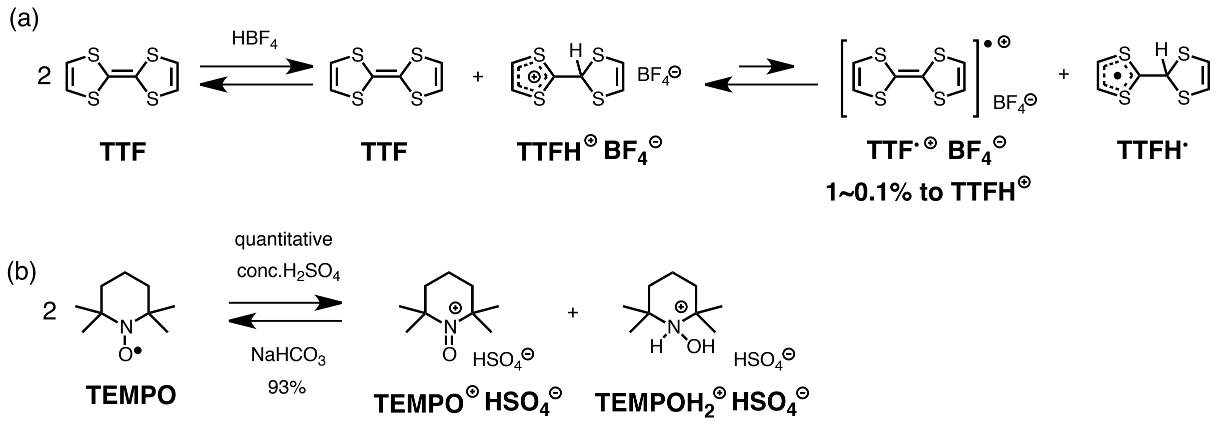

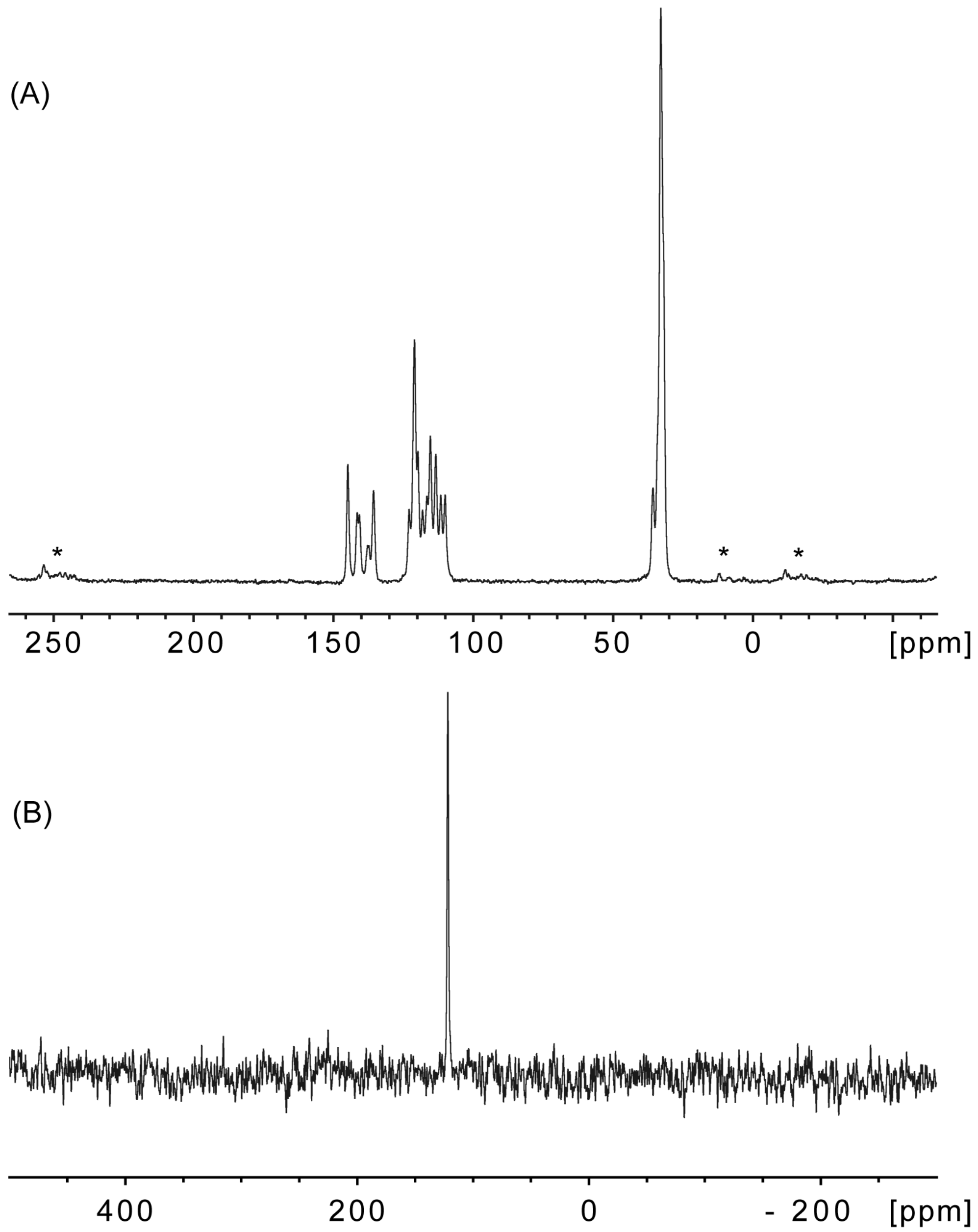

Fig. S1 (A) ^{13}C- and (B) ^{15}N-CPMAS solid state NMR spectra of BC. Contact time was 3 ms. MAS frequencies for ^{13}C- and ^{15}N CPMAS were 20 and 10 kHz, respectively. Asterisks indicate spinning side bands of aromatic backbone signals. Number of scans were 8000 and 220000 for ^{13}C- and ^{15}N CPMAS, respectively.

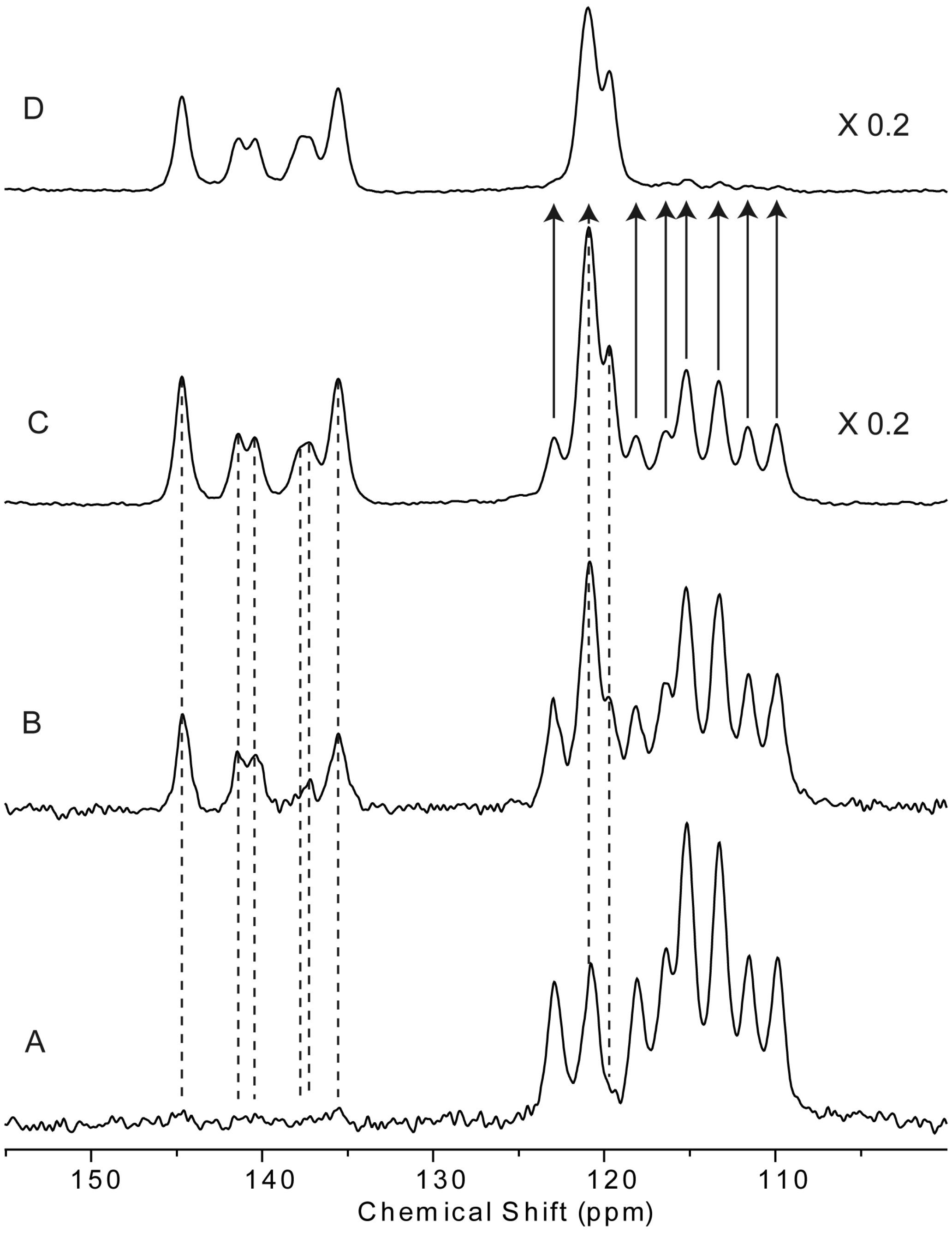

Fig. S2 ^{13}C-CP- and LGCPMAS solid state NMR spectra for aromatic regions of BC for various contact times. MAS frequency was 10 kHz. (A), (B) ^{13}C-LGCP MAS at contact times of 60 and 200 μs, respectively. (C), (D) ^{13}C-CPMAS at contact time of 3 ms without and with dipolar dephasing. Dotted lines indicate protonated carbon sites. Arrows indicate intensity reduced peaks due to recoupled ^{1}H-^{13}C heteronuclear dipolar interactions between ^{13}C and attached ^{1}H. Numbers of scans were 5000 for all experiments.

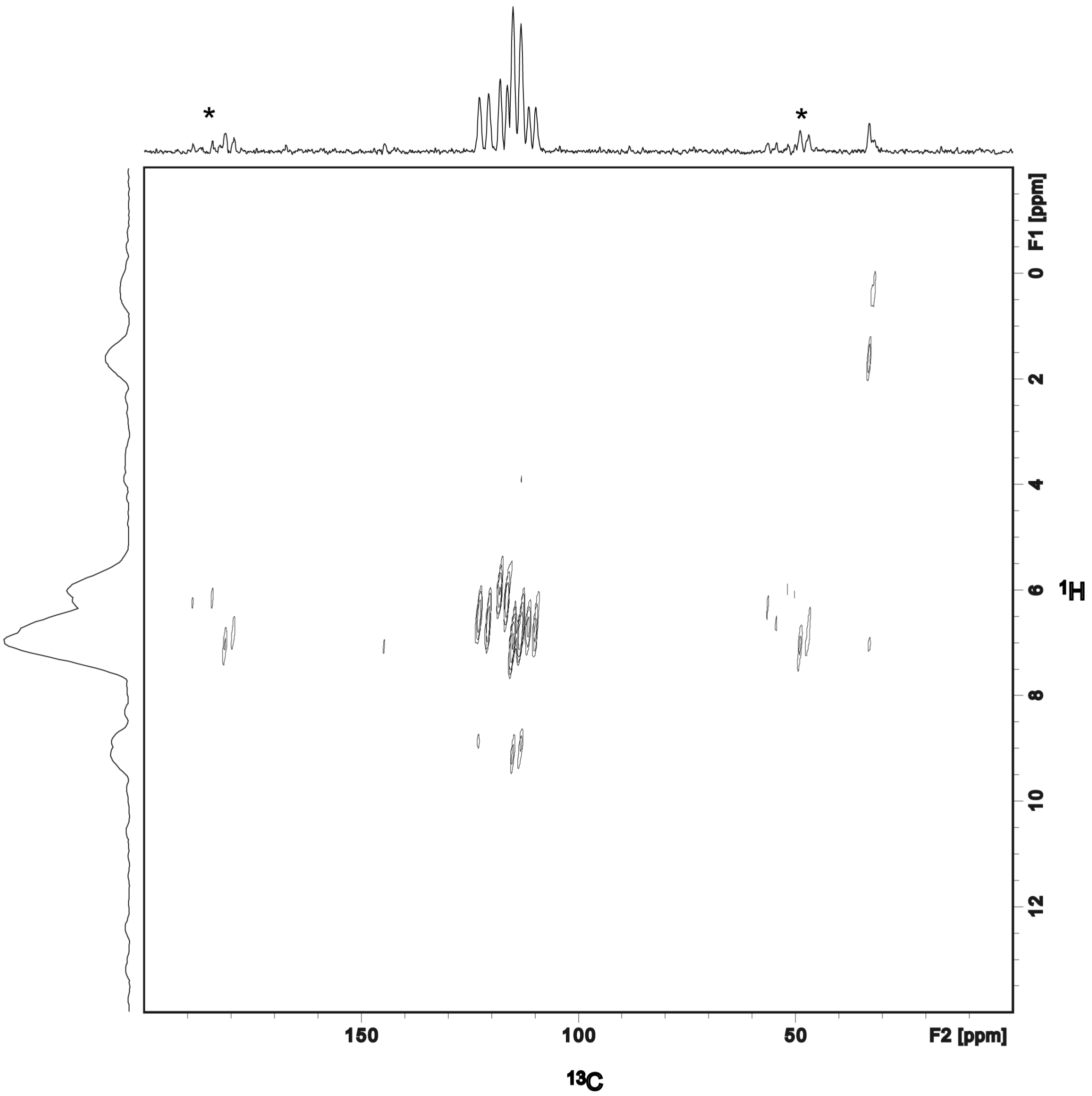

Fig. S3 ^{1}H-^{13}C FSLG heteronuclear correlation spectrum of BC. MAS frequency was 10 kHz. Contact time of LGCPMAS was set to 60 μs to correlate ^{13}C and attached ^{1}H. Isotropic chemical shifts of ^{1}H attached to ^{13}C were obtained for individual sites as summarized in Table S1. Asterisks indicate spinning side bands of aromatic backbone signals.

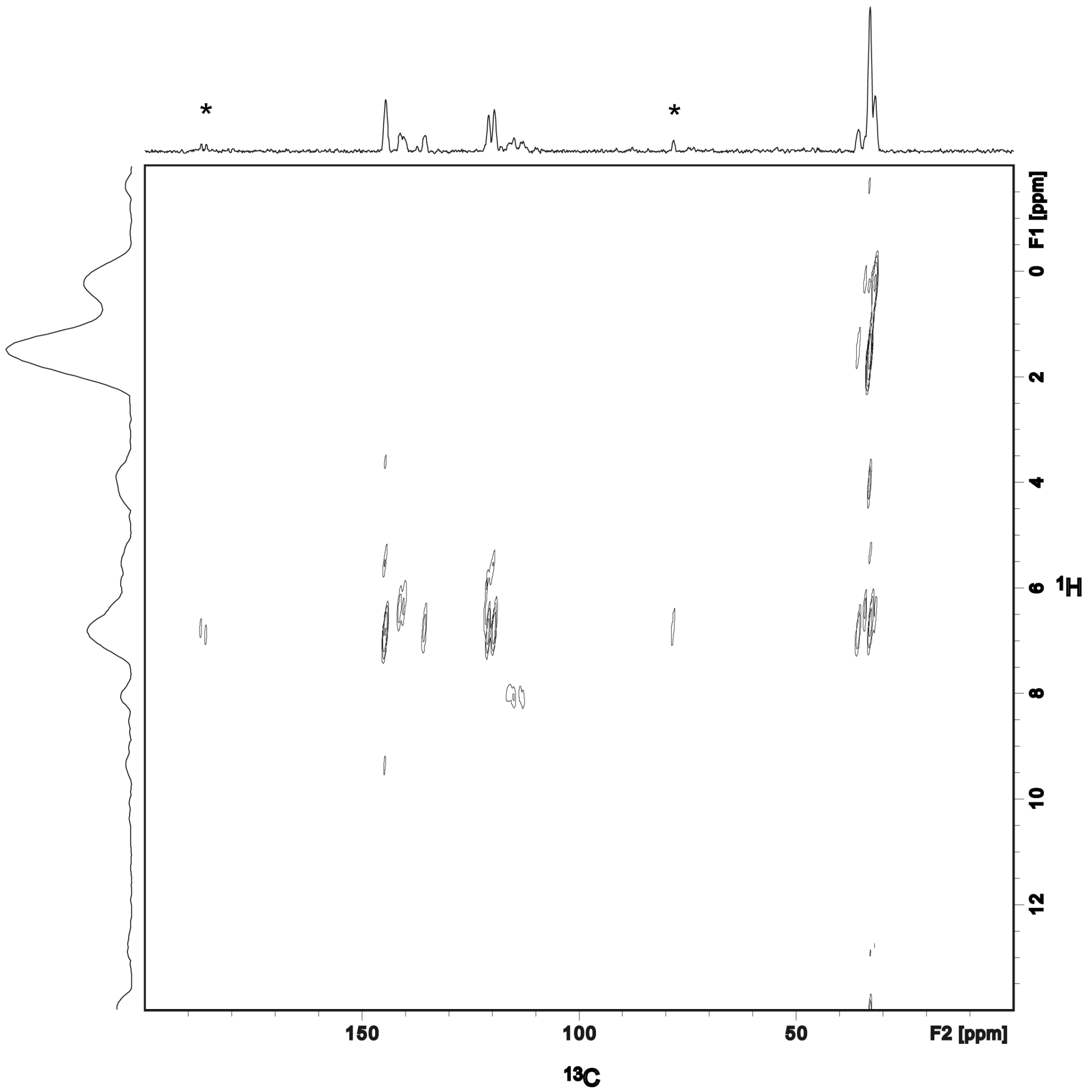

Fig. S4 ^{1}H-^{13}C MELODI heteronuclear correlation spectrum of BC. MAS frequency was 10 kHz. Contact time of LGCPMAS was set to 240 μs to correlate $^{13}C$and adjacent^{1}H. Asterisks indicate spinning side bands of aromatic backbone signals.

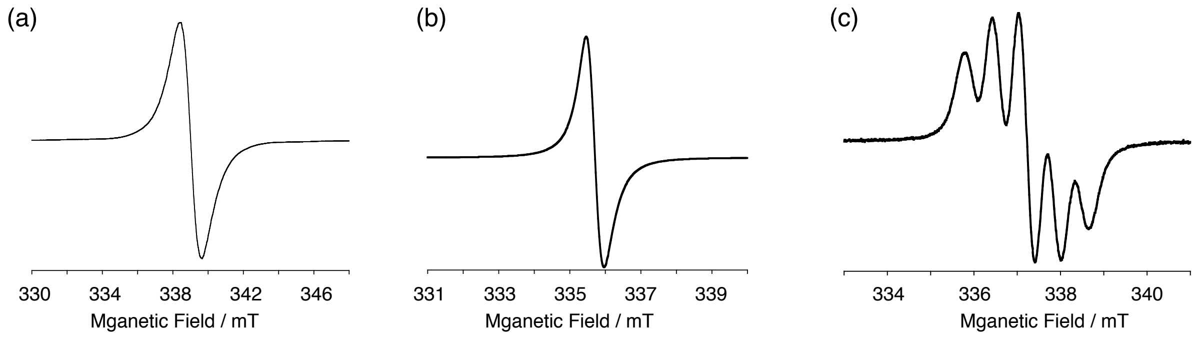

Fig. S5 (a) ESR spectrum of BC (1.02 mM) with 2000 mol% CF_3CO_2H in frozen CH_2Cl_2 at 5 K (X-band, ν = 9.507335 GHz, *g* = 2.0036). (b) ESR spectrum of $BC^{\bullet+}I_5^-\cdot IC_6H_5$ crystal at room temperature (X-band, ν = 9.426200 GHz). (c) ESR spectrum of $BC^{\bullet+}I_5^-\cdot IC_6H_5$ crystal dissolved in MeOH at room temperature (X-band, ν = 9.457425 GHz).

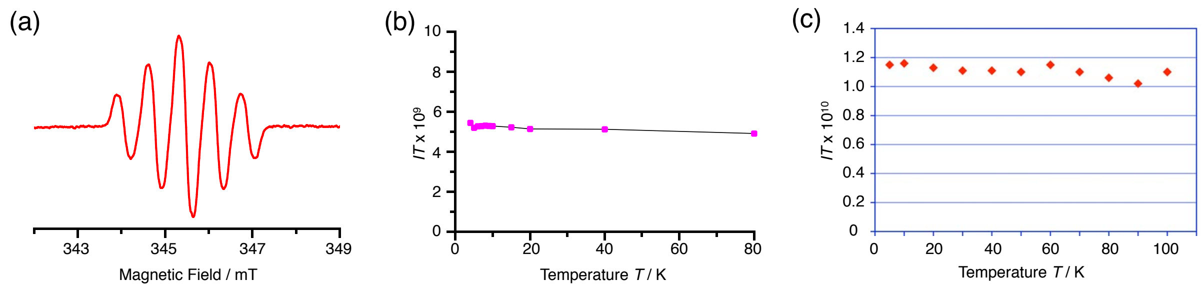

Fig. S6 (a) ESR spectrum of TBA (0.100 mM) with 2000 mol% CF_3CO_2D in CH_2Cl_2 at room temperature (X-band, *v* = 9.688112 GHz, *g* = 2.0036). (b) Temperature dependence of *IT* value of TBA with 2000 mol% CF_3CO_2H in frozen CH_2Cl_2 at 5-80 K. (c) Temperature dependence of *IT* value of TEMPOL in frozen CH_2Cl_2 at 5-100 K.

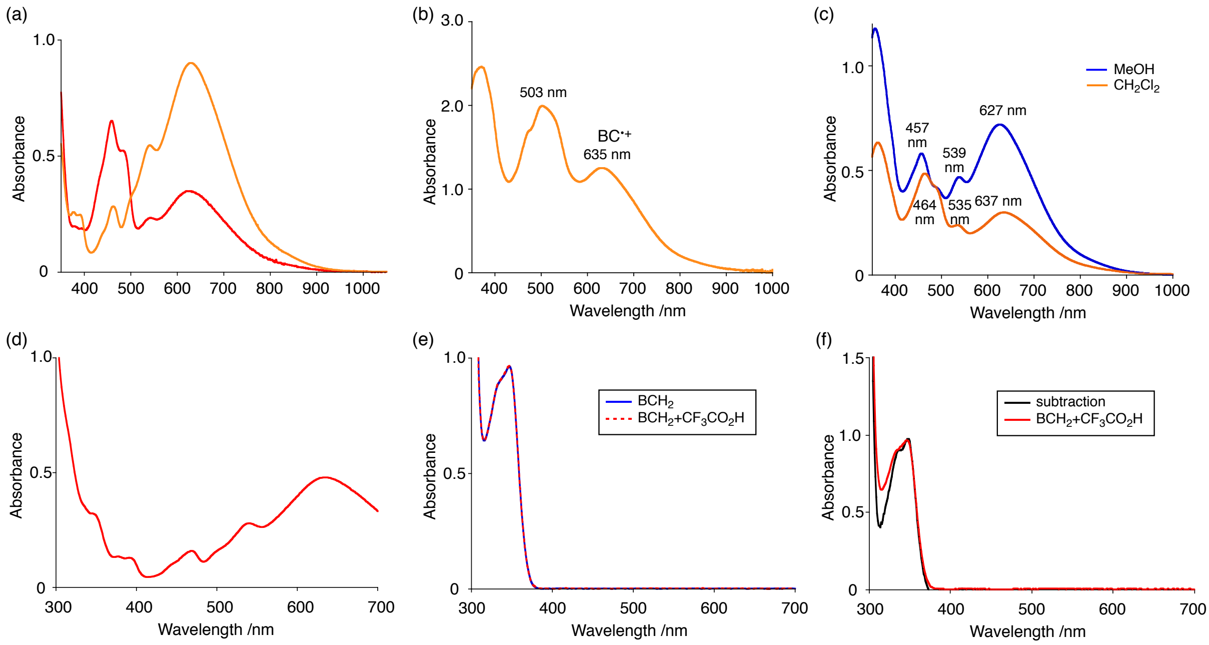

Fig. S7 (a) UV-Vis-NIR spectrum of BC (1.00 mM) with 2000 mol% CF_3CO_2H in CH_2Cl_2 (orange) and the spectra of the re-prepared solution after evaporation of excess CF_3CO_2H in CH_2Cl_2 (red) with 1 mm cell (see 7.1). (b) UV-Vis-NIR spectra of $BC^{\bullet+}$ by the chemical oxidation of BC (0.100 mM) with I_2 (3000 mol%) in CH_2Cl_2. (c) UV-Vis-NIR spectra of $BC^{\bullet+}I_5^{-}\cdot IC_6H_5$ crystal dissolved in MeOH (0.090 mM) or CH_2Cl_2 (0.056 mM). (d) UV-Vis-NIR spectrum of BC (0.05 mM) with 2000 mol% CF_3CO_2H in CH_2Cl_2. (e) UV-Vis-NIR spectra of BCH_2 (0.100 mM) and BCH_2 (0.100 mM) with CF_3CO_2H (2000 mol%) in CH_2Cl_2. (f) Subtracted spectrum (black) between the spectrum of $BC^{\bullet+}$ + BCH_3^{+} (Fig. S7d) and that of $BC^{\bullet+}$ (Fig. 9b) and the spectrum (red) of BCH_3^{+} (Fig. S7e). The subtracted spectrum was obtained from the equation [(Fig. S7d) - (Fig. 9b)×2/3] ×6.

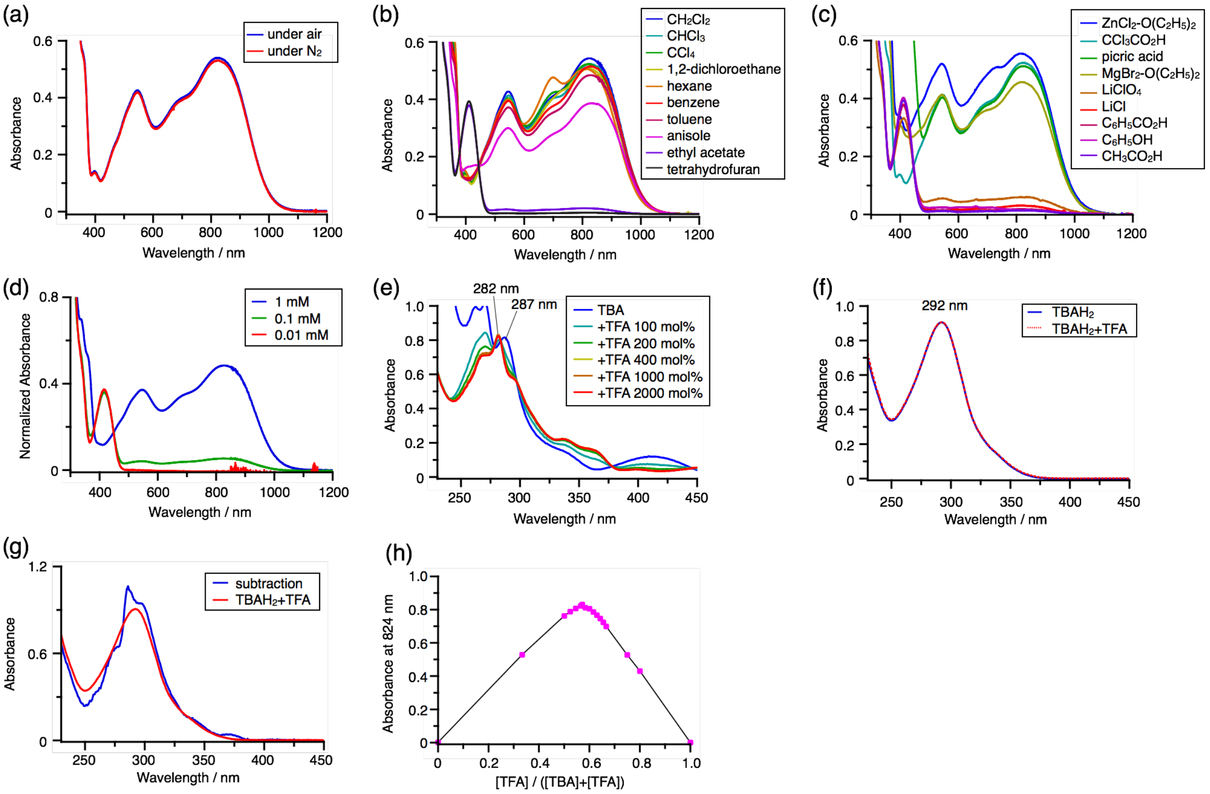

Fig. S8 (a) UV-Vis-NIR spectra of TBA (0.100 mM) with 2000 mol% CF_3CO_2H in CH_2Cl_2 under air and under N_2 atmosphere. (b) UV-Vis-NIR spectra of TBA (1.00 mM) with 2000 mol% CF_3CO_2H in various organic solvents. (c) UV-Vis-NIR spectra of TBA (0.100 mM) with 2000 mol% Brønsted acids and Lewis acids (10000 mol% for LiCl, CH_3CO_2H), in CH_2Cl_2. (d) UV-Vis-NIR spectrum of TBA (1.00 mM) with 2000 mol% CF_3CO_2H in toluene with 1 mm cell, that of TBA with 2000 mol% CF_3CO_2H diluted to 0.1 mM in toluene with 1 cm cell, and that of TBA with 2000 mol% CF_3CO_2H diluted to 0.01 mM in toluene with 1 cm cell. The spectrum of 0.01 mM was normalized to 10 times. (e) The short-wavelength region of UV-Vis-NIR spectra of TBA (0.030 mM) with 0-2000 mol% CF_3CO_2H in CH_2Cl_2. (f) UV-Vis-NIR spectra of $TBAH_2$ (0.030 mM) and $TBAH_2$ (0.030 mM) with 2000 mol% CF_3CO_2H in CH_2Cl_2. (g) Subtracted spectrum (blue) between the spectrum of $TBA^{\bullet+}$ + $TBAH_4^{2+}$ (Fig. S8e, TFA 2000 mol%) and that of $TBA^{\bullet+}$ (Fig. 16i, $NOPF_6$) and the spectrum (red) of $TBAH_4^{+}$ (Fig. S8f). The subtracted spectrum was obtained from the equation [(Fig. S8e) - (Fig. 16i)×3/10×2/3]×3. (h) Continuous variation plots of the absorbance of TBA with CF_3CO_2H at 824 nm in CH_2Cl_2 versus the ratio of $[CF_3CO_2H]/[TBA]+[CF_3CO_2H]$.

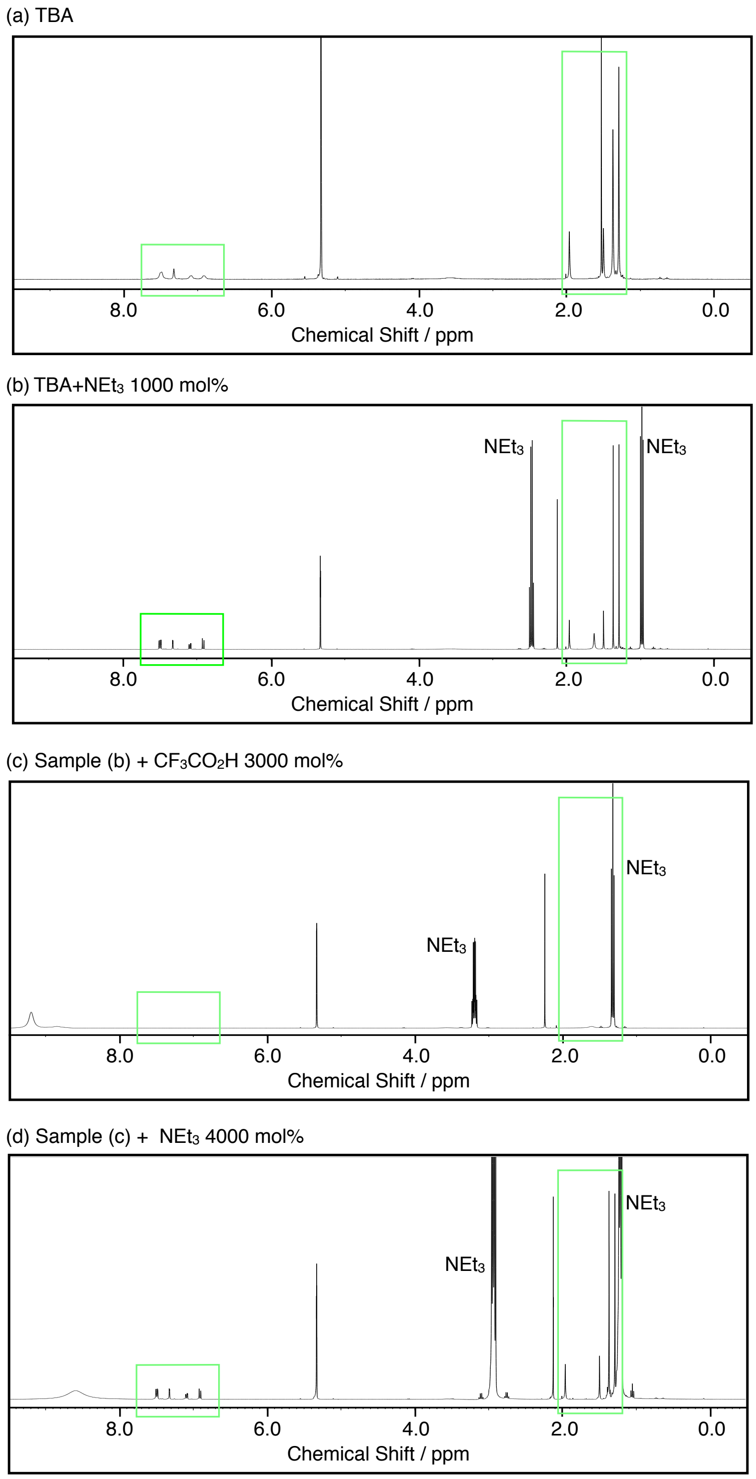

Fig. S9 (a) ^{1}H NMR spectrum of TBA (2.00 mM) in freshly distilled CD_2Cl_2, (b) ^{1}H NMR spectrum of TBA (2.00 mM) with 1000 mol% NEt_3 in CD_2Cl_2, (c) ^{1}H NMR spectrum of TBA with 1000 mol% NEt_3 [sample (b)] followed by acidification with 3000 mol% CF_3CO_2H in CD_2Cl_2, (d) ^{1}H NMR spectrum of recovered TBA with 1000 mol% NEt_3 and 3000 mol% CF_3CO_2H [sample (d)] followed by neutralization with 4000 mol% NEt_3 in CD_2Cl_2.

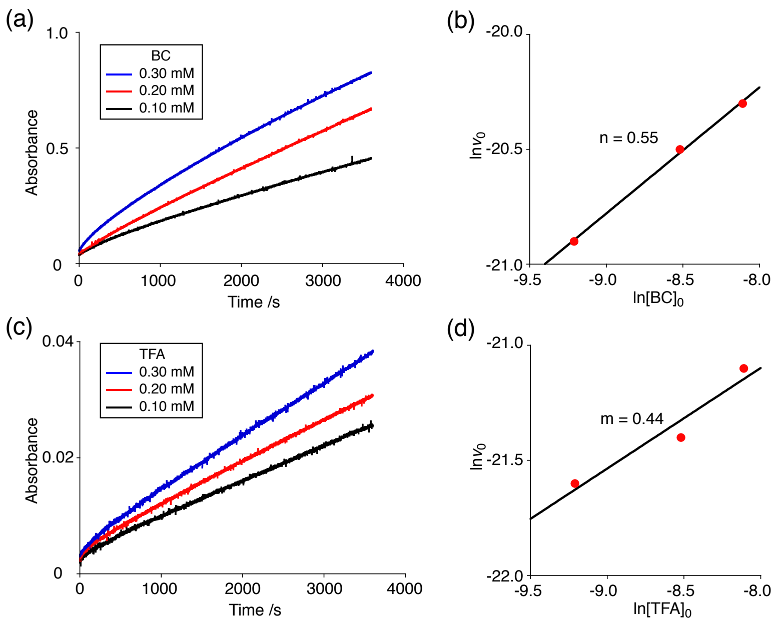

Fig S10. Determination of reaction orders in BC and TFA by initial rate method in benzene. (a) Time-dependent change of the absorbance of BC$^{\bullet+}$at 635 nm by mixing BC (0.10, 0.20, 0.30 mM) and TFA (5.00 mM) in benzene at 20 °C. (b) The reaction order in BC in benzene from the plot of ln[BC]$_0$ versus lnv_0 using the data in Fig. S10a. (c) Time-dependent change of the absorbance of BC$^{\bullet+}$at 635 nm by mixing BC (5.00 mM) and TFA (0.10, 0.20, 0.30 mM) in benzene at 20 °C. (d) The reaction order in CF_3CO_2H in benzene from the plot of ln[TFA]$_0$ versus lnv_0 using the data in Fig. S10c.

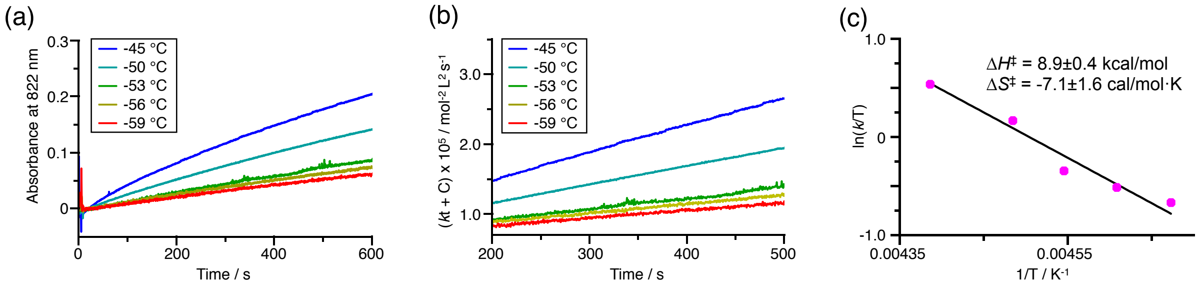

Fig S11. (a) Time-dependent change of absorbance at 822 nm by mixing 0.200 mM TBA and 0.800 mM CF_3CO_2H in CH_2Cl_2 at -45 to -59 °C for the determination of the kinetic constant *k*. (b) Determination of the kinetic constant *k* by fitting line using the data in Fig. S11. (c) Eyring plot using the data in Fig. S11a, b and the determined average enthalpy ($\Delta H^{\ddagger}$) and entropy ($\Delta S^{\ddagger}$) of activation.

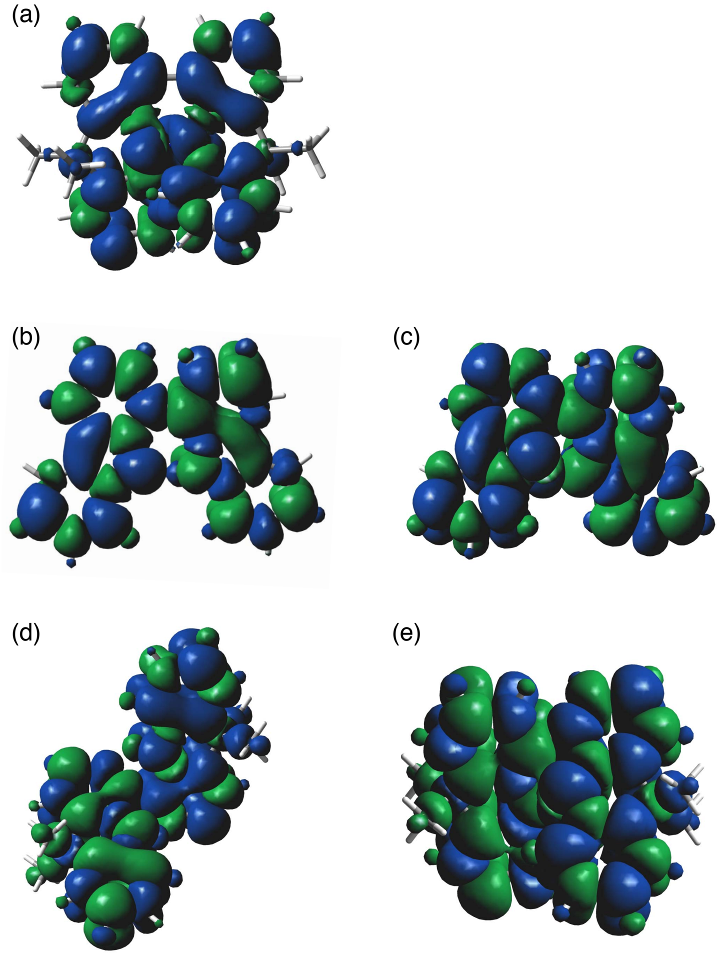

Fig. S12 (a) Calculated spin density of $TBA^{\bullet+}$. (b) Calculated spin density of $BCH^{\bullet\bullet+}$. (c) Calculated spin density of $BCH^{\bullet\bullet}$. (d) Calculated spin density of $TBAH_2^{\bullet\bullet 2+}$. (e) Calculated spin density of $TBA^{\bullet\bullet}$. Blue and green colors indicate the positive and negative spin density, respectively.

12. References

1. J. F. Araneda, W. E. Piers, B. Heyne, M. Parvez and R. McDonald, *Angew. Chem. Int. Ed.* 2011, **50**, 12214.
2. WO 2012/032990. PCT/JP2011/69849.
3. A. E. Bennett, C. M. Rienstra, M. Auger, K. V. Lakshmi and R. G. Griffin, *J. Chem. Phys.* 1995, **103**, 6951.
4. B. M. Fung, A. K. Khitrin and K. Ermolaev, *J. Magn. Reson.* 2000, **142**, 97.
5. M. Lee and W. I. Goldburg, *Phys. Rev.* 1965, **140**, A1261.
6. R. K. Hester, J. L. Ackerman, V. R. Cross and J. S.Waugh, *Phys. Rev. Lett.* 1975, **34**, 993.
7. S. J. Opella and M. H. Frey, *J. Am. Chem. Soc.* 1979, **101**, 5854.
8. B.-J. van Rossum, C. P. de Groot, V. Ladizhansky, S. Vega and H. J. M. de Groot, *J. Am. Chem. Soc.* 2000, **122**, 3465.
9. X. L. Yao, K. Schmidt-Rohr and M. Hong, *J. Magn. Reson.* 2001, **149**, 139.
10. K. Takegoshi, Take NMR 1.42., Kyoto University, Japan.
11. T. D. Goddard and D. G. Kneller, SPARKY 3, University of California, San Francisco, http://www.cgl.ucsf.edu/home/sparky/.
12. W. H. Melhuish, *J. Phys. Chem*. 1961, **65**, 229.
13. A. T. R. Williams, S. A. Winfield and J. N. Miller, *Analyst* 1983, **108**, 1067.
14. G. Heinrich, S. Schoof and H. Gusten, *J. Photochem*. 1974/75, **3**, 315.
15. Gaussian 09, Revision D.01., M. J. Frisch, G. W. Trucks, H. B. Schlegel, G. E. Scuseria, M. A. Robb, J. R. Cheeseman, G. Scalmani, V. Barone, B. Mennucci, G. A. Petersson, H. Nakatsuji, M. Caricato, X. Li, H. P. Hratchian, A. F. Izmaylov, J. Bloino, G. Zheng, J. L. Sonnenberg, M. Hada, M. Ehara, K. Toyota, R. Fukuda, J. Hasegawa, M. Ishida, T. Nakajima, Y. Honda, O. Kitao, H. Nakai, T. Vreven, J. A. Montgomery, Jr., J. E. Peralta, F. Ogliaro, M. Bearpark, J. J. Heyd, E. Brothers, K. N. Kudin, V. N. Staroverov, T. Keith, R. Kobayashi, J. Normand, K. Raghavachari, A. Rendell, J. C. Burant, S. S. Iyengar, J. Tomasi, M. Cossi, N. Rega, J. M. Millam, M. Klene, J. E. Knox, J. B. Cross, V. Bakken, C. Adamo, J. Jaramillo, R. Gomperts, R. E. Stratmann, O. Yazyev, A. J. Austin, R. Cammi, C. Pomelli, J. W. Ochterski, R. L. Martin, K. Morokuma, V. G. Zakrzewski, G. A. Voth, P. Salvador, J. J. Dannenberg, S. Dapprich, A. D. Daniels, O. Farkas, J. B. Foresman, J. V. Ortiz, J. Cioslowski, D. J. Fox,Gaussian, Inc., Wallingford CT, 2013.
